# Polarized Helical Coumarins: [1,5] Sigmatropic Rearrangement and Excited-State Intramolecular Proton Transfer

**DOI:** 10.1021/acs.joc.0c02978

**Published:** 2021-04-08

**Authors:** Łukasz Kielesiński, Olaf W. Morawski, Cristina A. Barboza, Daniel T. Gryko

**Affiliations:** † Institute of Organic Chemistry of Polish Academy of Sciences, Kasprzaka 44/52, 01-224 Warsaw, Poland; ‡ Institute of Physics of Polish Academy of Sciences, Al. Lotników 32/46, 02-668 Warsaw, Poland

## Abstract

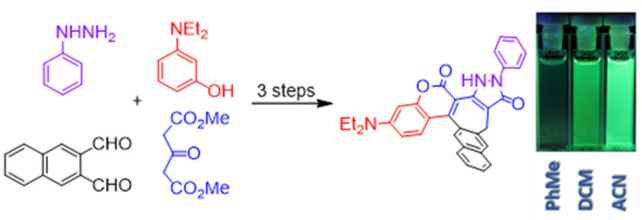

The tandem process of phenol addition to a cyclic α,β-unsaturated ester followed by intramolecular transesterification and [1,5] sigmatropic rearrangement affords a series of helical coumarins based upon a previously unknown 3-amino-7-hydroxybenzo[3,4]cyclohepta[1,2-*c*]chromen-6-one core. These novel polarized coumarins, possessing a β-ketoester moiety, have been employed to synthesize more rigid and helical coumarin–pyrazolones, which display green fluorescence. The enhanced emission of coumarin–pyrazolones in polar solvents depends on the nature of the *S*_1_ state. The coumarin–pyrazolones are predicted to have two vertical states close in energy: a weakly absorbing *S*_1_ (^1^LE) followed by a bright *S*_2_ state (^1^CT). In polar solvents, the ^1^CT can be stabilized below the ^1^LE and may become the fluorescent state. Solvatochromism of the fluorescence spectra confirms this theoretical prediction. The presence of an N—H···O=C intramolecular hydrogen bond in these coumarin–pyrazolone hybrids facilitates excited-state intramolecular proton transfer (ESIPT). This process leads to a barrierless conical intersection with the ground electronic state and opens a radiationless deactivation channel effectively competing with fluorescence. Solvent stabilization of the CT state increases the barrier for ESIPT and decreases the efficiency of the nonradiative channel. This results in the observed correlation between solvatochromism and an increase of fluorescence intensity in polar solvents.

## Introduction

**Scheme 1:**
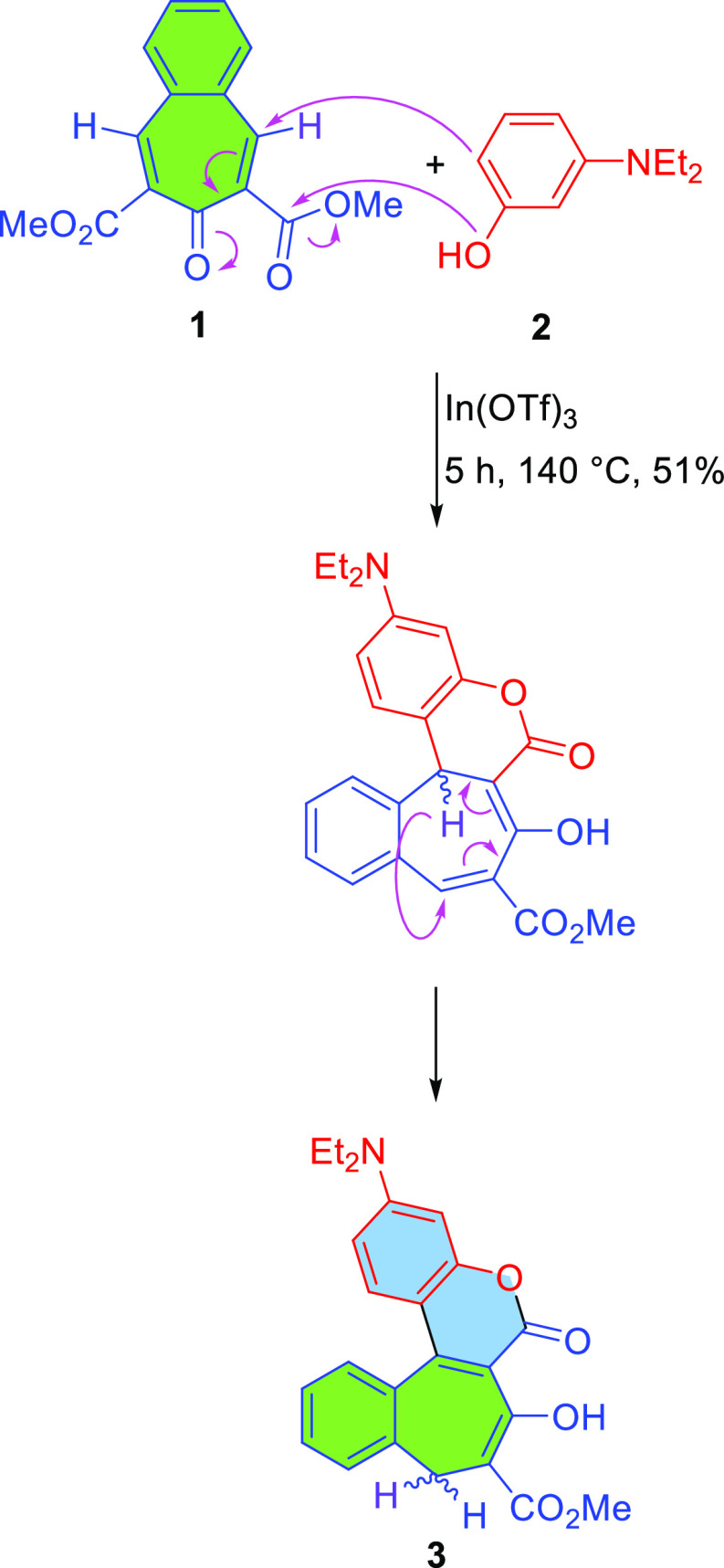
Synthesis of Dye **3** Having a Coumarin Motif Fused to a Seven-Membered Ring and the Proposed Mechanism of This Transformation

The rapid progress of modern technologies has relied on the design, creation, and implementation of new materials. Particular attention has been focused on novel organic chromophores as, unlike their inorganic counterparts, they do not contain rare, expensive, or unethically mined elements. Coumarins are opportune fluorophores for many applications as the introduction and modification of substituents on this scaffold has been shown to enable comprehensive control of their photophysical properties.^1^ Their special features, such as large fluorescence quantum yield, large Stokes shift, high absorption coefficient, and photostability, make them very desirable and allow them to be used as fluorescent probes,^2^ as dyes for two-photon fluorescence microscopy,^3^ in OLEDs,^4^ and as constituents in energy and electron transfer systems.^5^ Another important modification that allows for coumarins’ bandgap tuning is π-electron core expansion.^6^ This allows the absorption and fluorescence to be red-shifted, a feature that is particularly important for fluorescence imaging.^7^ Multiple examples of coumarin chromophore modification have been published recently.^8^ Among many scaffolds, the V-shaped bis-coumarins, which can be synthesized by the Michael reaction of electron-rich phenols with esters of coumarin-3-carboxylic acids,^9^ have attracted broader attention.^10^ Independently, it was reported that coumarins possessing an electron-withdrawing group at position 3 undergo Michael addition with other nucleophiles to form dihydrocoumarins^11^ or coumarins.^12^ In both the latter case and in the case of phenol addition,^9^ a second molecule of coumarin ester acts as an oxidant. In light of these results, we have designed an intramolecular version of such a process. The hypothesis was that, if two α,β-unsaturated ester groups are present within a molecule, one will act as an electrophilic coupling partner and the second will take the role of an oxidant. We envisioned that the replacement of coumarin with ester **1** (Scheme 1) would lead to not only promising dyes containing a coumarin moiety fused with a seven-membered ring but also core-modified [4]helicenes.

## Results and Discussion

### Synthesis

Guided by the above-described hypothesis, we designed a reaction between dimethyl 7-oxo-7*H*-benzo[7]annulene-6,8-dicarboxylate (**1**)^13^ and 3-diethylaminophenol (**2**) as our model system (Scheme 1). Encouraged by our recent discovery of the reaction of electron-rich phenols with esters of coumarin-3-carboxylic acids, leading to conjoined coumarins,^9^ we used the same conditions herein; i.e., the reaction was performed neat and with indium triflate as a catalyst. These conditions allowed product **3** to be obtained in a satisfactory 51% yield. Replacing In(OTf)_3_ with other Lewis acids and adding various solvents did not lead to an increase in yield. The potential product from the addition of two molecules of phenol to ester **1** was not detected in the crude reaction mixture as judged by ESI-MS. Attempts to oxidize dye **3** to the corresponding dye bearing a fully conjugated structure with numerous oxidizing agents failed.

The structure of compound **3** was unambiguously confirmed by ^1^H and ^13^C NMR experiments, which proved that the C–C double bond is located at the place of the Michael reaction resulting in the formation of the coumarin chromophore. In-depth analysis of the transformation of diester **1** into coumarin **3** led us to the conclusion that the plausible mechanism consists of Michael addition followed by [1,5] sigmatropic rearrangement (Scheme 1). [1,5] Sigmatropic rearrangement is a reaction in which a hydrogen atom migrates from one end of a system of π bonds to the other.^14^ An *S-cis* conformation has to be adopted by the molecule, since it has been established that typically a pericyclic mechanism is involved and the hydrogen atom has to, in the transition state, be in contact with both ends of the chain at the same time.^15^ In our case, the reaction must occur, based on orbital symmetry rules,^16^ in a suprafacial geometrical pathway since it is a thermal process.^17^ The driving force for the [1,5] sigmatropic rearrangement ([1,5] hydrogen shift) observed here is plausibly due to a different character of the initially intact olefinic bond versus the coumarin C–C double bond resulting from the presence of the Et_2_N group.

From the formal point of view, the discovered process is an example of an intramolecular redox process.^18^ It is noteworthy that coumarins have been previously synthesized by the condensation of alkyl cinnamates with phenols in the presence of transition metal salts.^19^ Molecule **3** has a [4]helicene architecture, hence axial chirality, which manifests itself through the methylene bridge hydrogen atoms in the seven-membered ring not being equivalent (deduced from the presence of two characteristic doublets in the ^1^H NMR spectrum, Figure S1a). The presence of the seven-membered ring makes the helical structure more curved.

**Figure 1:**
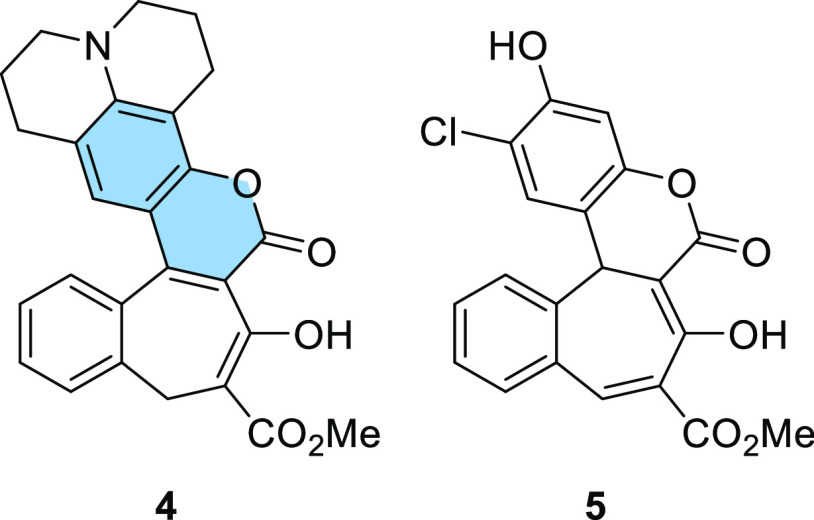
Structures of dyes **4** and **5**.

With this viable methodology, our attention turned to other electron-rich phenols. Using the same reaction conditions, we synthesized derivative **4** bearing a hydroxyjulolidine moiety (Figure 1). The yield of this product was good (77%), but during purification, we noticed that the compound was unstable and decomposed to byproducts observable in the ^1^H NMR spectrum. Among other starting materials tested, we also used 4-chlororesorcinol (**10**), which led to compound **5** in 54% yield (Figure 1). The main difference between hydroxy-substituted compound **5** and amino-substituted coumarins **3** and **4** is the fact that the [1,5] sigmatropic rearrangement does not occur at the reaction temperature (140 °C). As a result molecule **5** does not possess a coumarin scaffold (Figure 1). The analysis of the ^1^H NMR spectrum confirmed this difference. The spectrum for compound **5** showed two single signals at 4.34 and 8.41 ppm, which were assigned to the pyran ring and a double bond in the seven-membered ring, respectively (Figure S1b). This is in contrast to **3** which has two doublets present in the aliphatic region of the spectrum (Figure S1a).

**Scheme 2:**
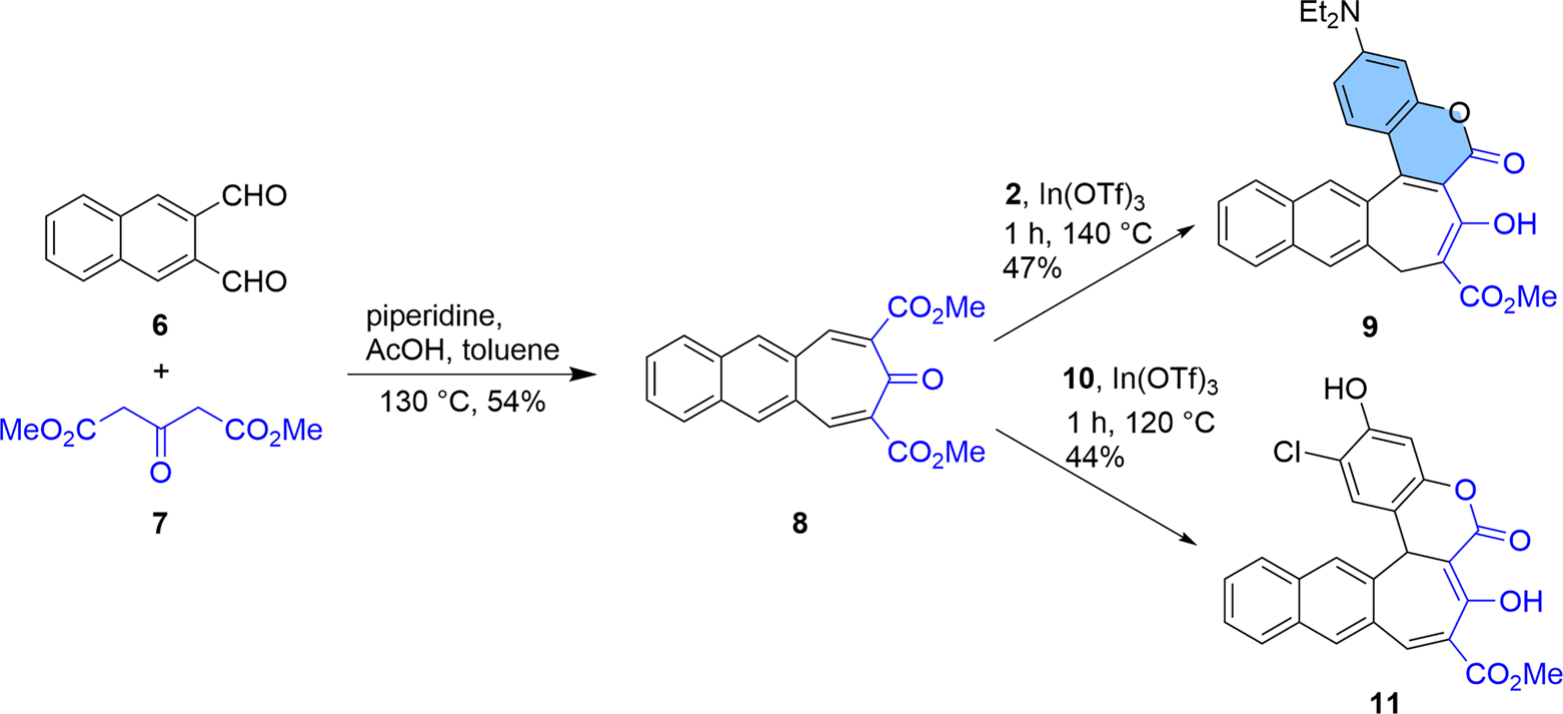
Synthesis of Dyes **9** and **11**

Using the above-discussed methodology, we decided to extend our studies to molecules possessing a similar structure but with one additional fused benzene ring. Initially, we synthesized the appropriate naphthalene-2,3-dicarbaldehyde (**6**), starting from phthalaldehyde and dimethoxytetrahydrofuran.^20^ The next step was a double Knoevenagel condensation of dialdehyde **6** with acetone-1,3-dicarboxylate, which allowed us to obtain compound **8** in 54% yield (Scheme 2). Subsequently, we carried out reactions with 3-diethylaminophenol (**2**) and 4-chlororesorcinol (**10**) in the presence of indium triflate, which led to compounds **9** and **11** in 47% and 44% yields, respectively.

Once we ascertained that cyclic, double α,β-unsaturated esters could be successfully engaged in a coumarin-forming reaction, we decided to capitalize on the fact that the structure of dyes **3**, **4**, **5**, **9**, and **11** gives rise to many synthetic opportunities due to their β-ketoester nature. Consequently, we attempted the synthesis of dyes with an additional pyrazolone ring, expecting the appropriate products to possess more rigid geometry. There are many examples described in the literature of pyrazolone ring formation by heating a hydrazine derivative with an appropriate 1,3-dicarbonyl substrate in acetic acid^21^ or ethanol.^22^ In order to test the suitability of our dyes for this purpose, we performed the reaction of coumarin **3** with phenylhydrazine (**12**) by heating the substrates in acetic acid. We obtained the expected product **13**, albeit in a very low yield (5%, Table 1). When the reaction was conducted in ethanol, we observed only traces of product. To facilitate the formation of dye **13**, we diverged from these traditional conditions, performing a short optimization study using a selection of catalysts (Table 1).

**Table 1:**
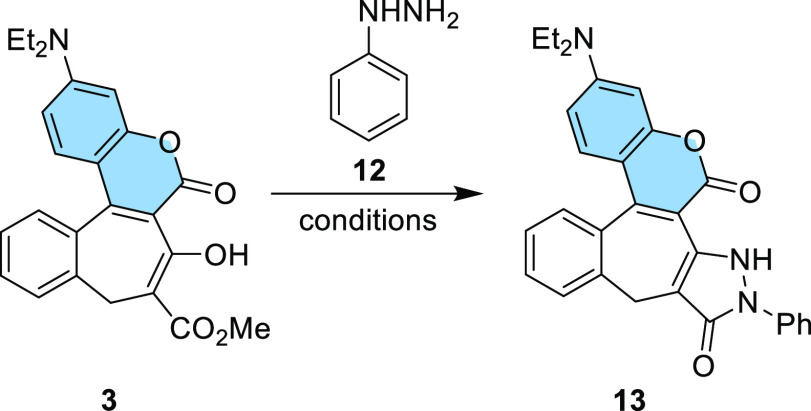
Optimization of the Synthesis of Pyrazolone **13**

entry	catalyst	solvent	time [h]	yield^a^ [%]
**1**		AcOH	12^b^	5
**2**		EtOH	24^b^	trace
**3**	Zn(NTf_2_)_2_	toluene	24^b^	0
**4**	DMAP	toluene	24^b^	17
**5**	*p*-TsOH	toluene	24^b^	59
**6**	AgOTf	toluene	24^b^	43
**7**	T_3_P in DCM	toluene	24^b^	72

a Isolated yields.

b Reflux.

**Scheme 3:**
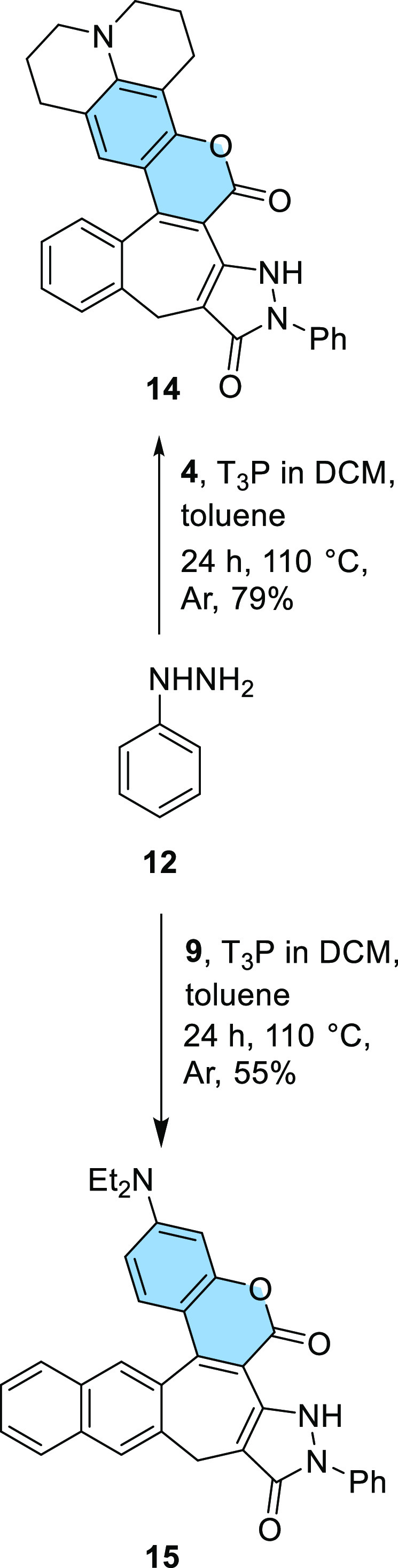
Synthesis of Coumarins **14** and **15**

The reaction in the presence of zinc di[bis(trifluoromethylsulfonyl)imide] failed. Using 4-dimethylaminopyridine allowed the final product to be obtained in the still low 17% yield. We obtained a better result when silver triflate^23^ was used (entry 6). In the presence of *p*-toluenesulfonic acid,^24^ we synthesized the product with a moderate to good yield (59%). The best result, however, was obtained when we used propylphosphonic acid cyclic anhydride in dichloromethane^25^ (T_3_P in DCM), a well-known reagent widely used in peptide chemistry. The reaction was conducted in toluene under an argon atmosphere, giving product **13** in 72% yield. With the optimized reaction conditions in hand, we decided to study the scope of this methodology. Starting from **4** and **9**, we obtained the new dyes containing an additional pyrazolone ring **14** and **15** in the good yields of 79% and 55%, respectively (Scheme 3).

**Scheme 4:**
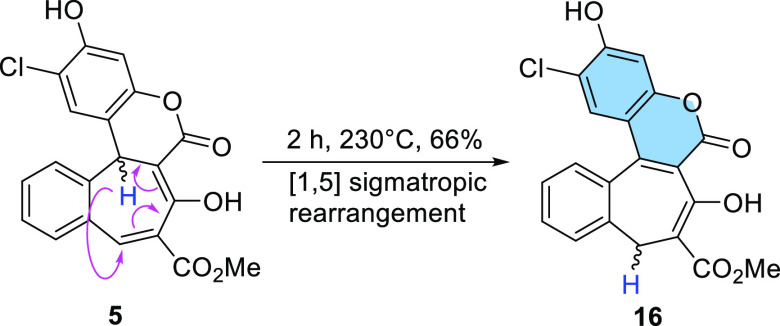
Synthesis of Coumarin **16**

We also investigated whether these conditions were suitable for molecules with substituents other than an amino group such as **5**. It turned out that, in this case, the conditions were ineffective. Only unreacted substrates were observed. The different outcomes of condensations of ester **1** with 3-dialkylaminophenols and with 4-chlororesorcinol prompted us to attempt to enforce the [1,5] sigmatropic rearrangement reaction in the case of heterocycle **5** by increasing the temperature. We heated compound **5** for 2 h under a vacuum without solvent (Scheme 4). It turned out that, under these conditions, the [1,5] hydrogen shift does occur, and coumarin **16** was obtained in 66% yield. The structure of dye **16** was confirmed by the ^1^H NMR spectrum.

### Single-Crystal X-ray Diffraction Studies

**Figure 2:**
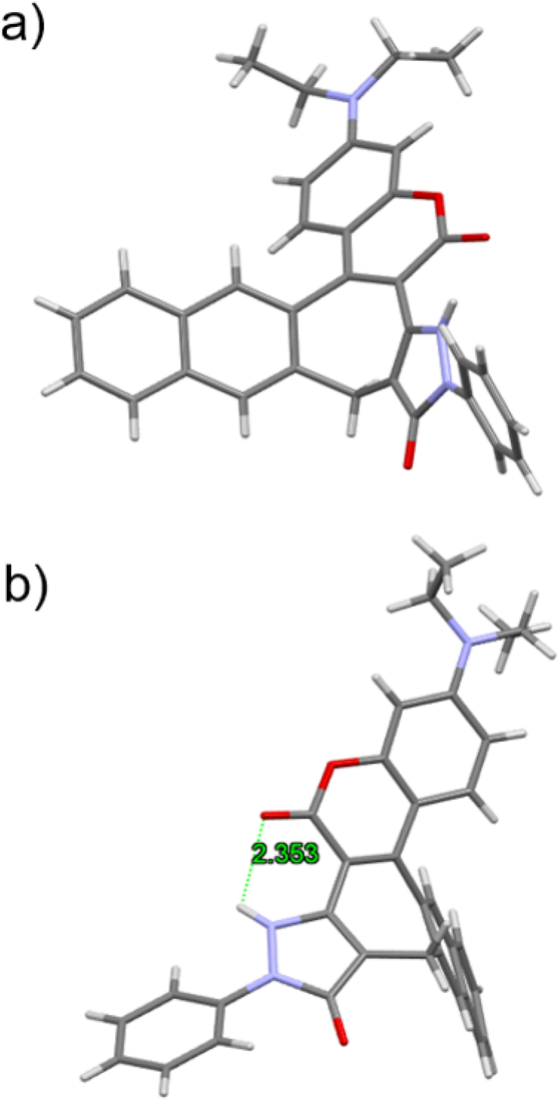
X-ray structure of compound **15** with the marked hydrogen bond: (a) front view and (b) side view.

Compound **15** crystallizes in yellow plates appropriate for X-ray analysis by slow diffusion of hexane into a dichloromethane solution. The crystallographic structure is presented in Figure 2. It belongs to the *P*2_1_/*c* space group. This molecule possesses an intramolecular hydrogen bond between the carbonyl group of the pyran-2-one motif and proton from the five-membered pyrazolone ring with a length of 2.353 Å (Figure 2b). The presence of the highly twisted seven-membered ring in compound **15** is the major contributing factor to its lack of planarity. The twist angle between the six-membered aromatic rings on either side of the seven-membered ring is ca. 61 degrees (Figure S4b). The dihedral angle between coumarin moiety and plane of pyrazolone ring is around 31 degrees (Figure S4a). The crystal packing in the unit cell is an antiparallel sandwich-type arrangement (Figure S3).

### Photophysical Results

**Figure 3:**
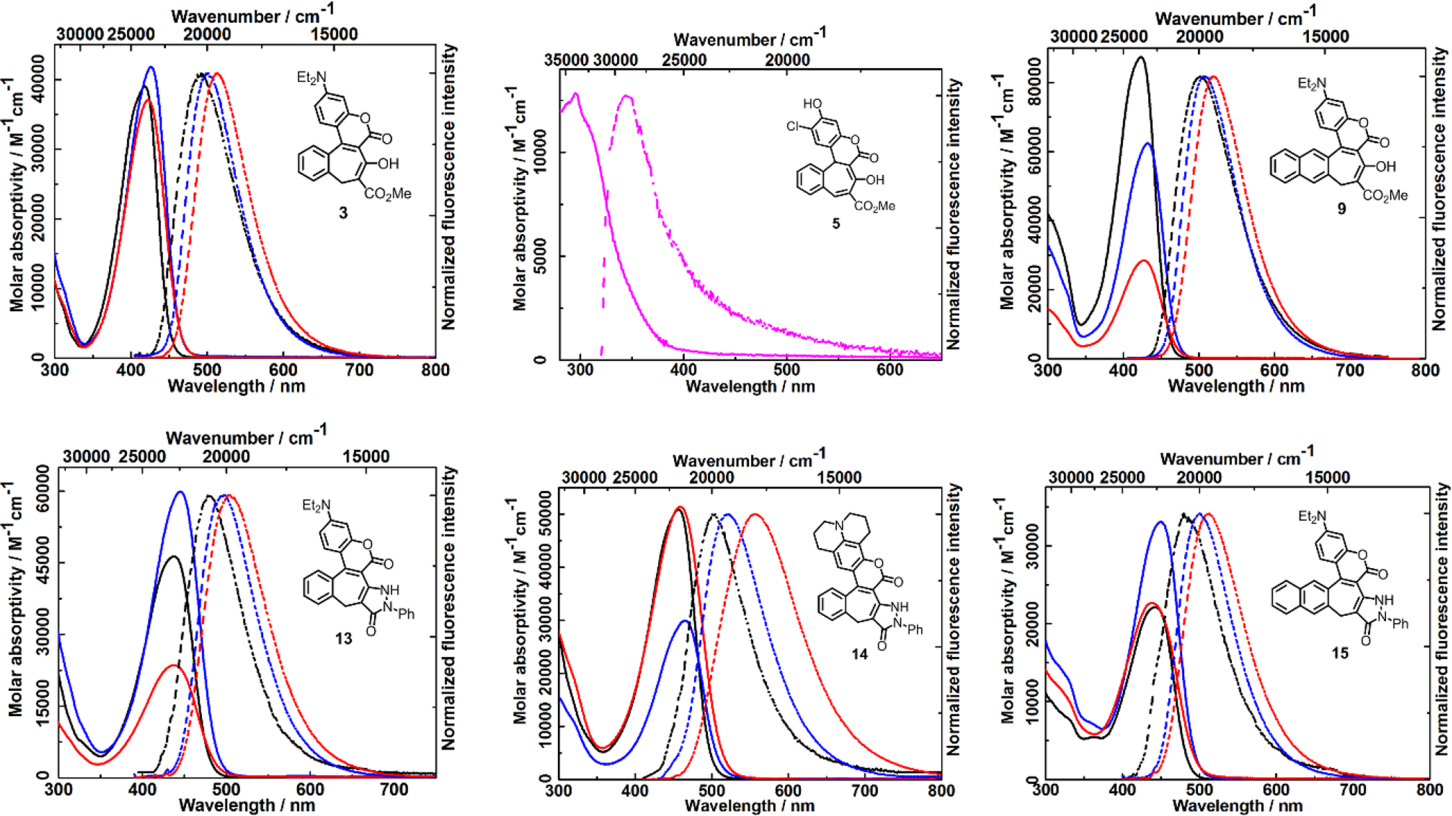
Absorption (solid line) and emission (dashed line) spectra of compounds **3** (excited at 395 nm), **5** (excited at 290 nm), **9** (excited at 395 nm), **13** (excited at 380 nm), **14** (excited at 400 nm), and **15** (excited at 390 nm) in toluene (black), DCM (blue), ACN (red), and dioxane (magenta).

Optical studies were performed for dyes **3**, **9**, **14**, **13**, and **15** in three solvents: nonpolar toluene (dielectric constant ε = 2.379), dichloromethane (DCM, ε = 8.93), and very polar acetonitrile (ACN, ε = 38.8). Compounds containing a chlorine atom (**5** and **11**) have a very low solubility in these solvents and were, therefore, only studied in 1,4-dioxane (ε = 2.25). Absorption and fluorescence spectra of all compounds are presented in Figure 3 and Figure S5b, and spectroscopic properties are summarized in Table 2.

**Table 2:** Spectroscopic Properties of Compounds **3**, **5**, **9**, **11**, **13**, **14**, **15**, and **16** Obtained in Toluene, DCM, ACN, and Dioxane

compound	solvent	λ_abs_^max^ [nm]	λ_em_^max^ [nm]	ε [M^–1^ cm^–1^]	Stokes shift [cm^–1^]	Φ_F_	τ_avr_ [ns]	*k*_r_^d^ [ns]	*k*_nr_^e^ [ns]
**3**	toluene	418	492	39000	3600	0.02^a^	0.076	0.26	12.9
	DCM	426	501	42000	3500	0.09^a^	0.217	0.42	4.19
	ACN	423	513	37100	4100	0.15^a^	0.95	0.16	0.89
**5**	dioxane	295	344	12900	4800	0.004^b^			
**9**	toluene	423	501	87400	3700	0.07^a^	0.144	0.49	6.46
	DCM	432	508	62400	3500	0.22^a^	0.365	0.60	2.14
	ACN	428	519	28500	4100	0.32^a^	0.645	0.50	1.05
**11**	dioxane	320	345	17300	2300	0.002^b^			
**13**	toluene	438	478	46300	1900	0.006^a^	1.23	0.005	0.81
	DCM	445	499	59900	2400	0.23^a^	1.79	0.13	0.43
	ACN	438	505	23600	3000	0.15^a^	1.73	0.087	0.49
**14**	toluene	457	504	50900	2000	0.01^a^	0.85	0.012	1.17
	DCM	465	522	30000	2300	0.20^a^	3.56	0.056	0.23
	ACN	458	556	51400	3800	0.19^a^	1.14	0.17	0.71
**15**	toluene	442	480	22100	1800	0.003^a^	0.94	0.003	1.06
	DCM	450	500	33100	2200	0.17^a^	3.52	0.048	0.24
	ACN	437	513	22700	3400	0.09^a^	3.1	0.029	0.29
**16**	toluene	357	489	21300	7600	0.0015^c^	0.012	0.125	83.2
	DCM	357	483	22200	7300	0.0013^c^	0.018	0.072	55.4

a Fluorescence quantum yield measured with perylene in cyclohexane (Φ_F_ = 0.94).

b Quinine sulfate in 0.5 M aqueous solution of H_2_SO_4_ (Φ_F_ = 0.54) as references.

c Using an integrating sphere.

d *k*_r_ = Φ_F_/τ_avr_.

e *k*_nr_ = 1/τ_avr_ – *k*_r_.

Compounds **3** and **9** exhibit maximum absorption and emission in the range of 418–432 nm and 492–519 nm, respectively (Table 2 and Figure 3). These values are similar to the structurally analogous coumarin possessing a diethylamino substituent at position 7 and COCH_2_CO_2_Me substituent at position 3 (**Cum1**) (Figure S2).^26^ Compounds **5** and **11** possess significantly blue-shifted absorption and emission spectra (Figure 3 and Figure S5b).^27^ Dyes **13**, **14**, and **15** containing a pyrazolone ring exhibit bathochromically shifted (only by a few nm) absorption and emission spectra (Table 2 and Figure 3) compared to the model compound (**Cum2**) (Figure S2).^28^**Cum2** exhibits a strong hypsochromic shift of the absorption band with increasing solvent polarity and slightly weaker shift in the emission spectra. The extension of the π-framework negligibly redshifts absorption/emission bands of **9** with respect to **3**. Stopping the rotation of the dialkylamino group (**13** → **14**) leads to a significant bathochromic shift of both absorption and emission maxima (ca. 20 nm). Interestingly, transforming the β-ketoesters into pyrazol-2-ones with concomitant rigidification of the dye’s architecture (**3** → **13**, **9** → **15**) results in a hypsochromic shift in emission. In our helical coumarins **13**, **14**, and **15**, the solvatochromism in absorption spectra is negligible, while, in the emission spectra, the maxima were bathochromically shifted with increasing solvent polarity, proving that the small ground state dipole moment increases upon optical excitation (Table 2 and Figure 3). Absorption spectra of compounds **3**, **9**, **13**, **14**, and **15** are dominated by a strongly allowed band with a maximum at 420–450 nm (2.95–2.75 eV) with molar absorptivity ranging from 30000 to 80000 M^–1^ cm^–1^ and exhibiting small solvatochromism (Table 2). Regarding their fluorescence, the solvent polarity dependence increases in the order **3** < **9** < **13** < **15** < **14**. These observations are directly correlated to the computed dipole moments for these molecules and explored in the theoretical session. The dyes containing a 1-phenylpyrazol-5-one moiety are significantly more polarized, and consequently, their solvatochromism is significantly more pronounced. The overlap of fluorescence and absorption spectra (Stokes shift ranges from 3000 to 4000 cm^–1^) visible in Figure 3 confirms that the emission originates from the *S*_1_ state. Indeed, the fluorescence quantum yield of all discussed compounds, about 0.01 to 0.2 (Φ_F_ in Table 2), indicates the operation of efficient radiationless processes for this series of compounds. For each given compound, the Φ_F_ is higher in polar solvents than it is in nonpolar toluene. This finding suggests that *S*_1_ is significantly stabilized in polar microenvironments. Apparently, this stabilization increases the barrier for the nonradiative process and slows down *k*_nr_. Inspection of nonradiative rates presented in Table 2 confirms this supposition.

This type of photophysical behavior, i.e., an increase in the fluorescent quantum yield in polar solvents, has been already reported, although only for a rather limited number of dyes such as acridine, pyrene-3-carboxaldehyde, 7-methoxy-4-methylcoumarin, and its π-expanded analogues.^29^

Compounds **5** and **11**, which lack the coumarin chromophore, have much lower molar absorptivity (below 20000 M^–1^ cm^–1^, Table 2). Absorption spectra of both molecules are blue-shifted to the UV region, and the maximum of the main spectral band is observed at 295 nm (4.2 eV) and 320 nm (3.87 eV) for **5** and **11**, respectively. The observed substantial increase in the excited state energy suggests smaller electronic coupling in these molecular systems. The low-energy shoulder in the absorption spectrum of **11** and low-energy tail in the spectrum of **5** (Figure 3 and Figure S5b) suggest the presence of more than one absorbing species. The fluorescence spectra are also shifted toward higher energy. Two bands are clearly visible in the fluorescence spectrum of **11**, which may suggest the presence of two isomers. Fluorescence quantum yields, 0.004 and 0.002 for **5** and **11**, respectively, prove that the de-excitation paths in these molecules are dominated by radiationless transitions.

**Figure 4:**
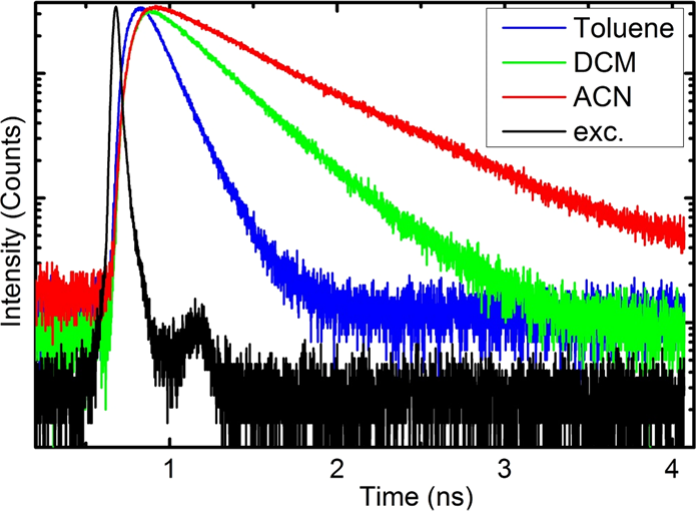
Semilogarithmic plot of fluorescence decays of compound **9** recorded at 21 °C. Excitation pulse 394 nm (black line) and fluorescence decays observed at 500 nm in toluene (blue), DCM (green), and ACN (red). Temporal resolution = 1.02 ps/channel.

In Figure 4, fluorescence decay traces of **9** recorded at room temperature are depicted along with the excitation pulse curve. Decays in polar solvents are monoexponential. In toluene, the dominant component has a short decay time, and the second component of a much longer decay time is visible. Single exponential decays are also observed in polar solvents for other compounds, with the exception of **13** and **15**, for which two exponential decay curves in toluene are also observed (Table 3, Figure S5a). Fast fluorescence decays in toluene and slow decay traces in polar solvents provide more experimental evidence for suppression of the radiationless processes discussed earlier. Nonradiative rate constants, *k*_nr_, calculated from fluorescence quantum yields (Φ_F_) and an averaged decay time, τ_avr_, are the highest in toluene for all molecules studied (Table 2). Thus, the supposition that interaction with a polar environment stabilizes the emitting charge transfer excited (^1^CT) state and inhibits the radiationless process is well-grounded.

**Table 3:** Fluorescence Decay Times (τ_1_, τ_2_) and the Amplitude of Decay Components (*A*_1_, *A*_2_) of Dyes **3**, **9**, **13**, **14**, **15**, and **16** in Toluene, DCM, and ACN

compound	solvent	τ_1_ [ns]	τ_2_ [ns]	*A*_1_	*A*_2_	τ_aver_ [ns]^a^
**3**	toluene	0.076				0.076
	DCM	0.195	1.46	57.2	1.0	0.217
	ACN	0.565	4.0	11.31	1.42	0.95
**9**	toluene	0.144				0.144
	DCM	0.365				0.365
	ACN	0.645				0.645
**13**	toluene	0.271	2.34	1.4	1.2	1.23
	DCM	1.790				1.79
	ACN	1.730				1.73
**14**	toluene	0.208	2.5	1.02	0.4	0.85
	DCM	3.56				3.56
	ACN	0.686	3.38	0.94	0.19	1.14
**15**	toluene	0.335	2.55	1.16	0.436	0.94
	DCM	3.52				3.52
	ACN	0.322	4.06	0.32	0.92	3.1
**16**	toluene	0.012				0.012
	DCM	0.018				0.018

a τ_aver_ = (*A*_1_τ_1_ + *A*_2_τ_2_)/(*A*_1_ + *A*_2_)

To explain the mechanism of this process, however, insight from theoretical calculations is required. Additionally, the absorption and emission spectra were measured for compound **16** in toluene and DCM (Figure S5b). In both cases, we observed significantly red-shifted bands compared to molecule **5** (Figure S5b and Figure 3). Compound **16** exhibited a larger Stokes shift too, which suggests an increase in dipole moment in the excited state as well as higher molar absorptivity resulting from the presence of coumarin chromophore (Table 2 and Scheme 4). Fluorescence quantum yields were also very low: only 0.0015 and 0.0013 for toluene and DCM, respectively. This proves that the relaxation of compound **16** is dominated by radiationless transitions (Table 2).

### Computational Results

Molecular model structures of **3**, **9**, **13**, and **15** were constructed with truncated alkyl chains (replacing the amine ethyl groups with methyl groups) to save computational time without significant effect on the electronic properties. The equilibrium geometries of all studied coumarin derivatives in their neutral or ionic closed-shell ground state (*S*_0_) were determined with the MP2 method^30^ without imposing any symmetry constrains. Excitation energies, equilibrium geometries, and corresponding properties of the lowest excited singlet states were calculated using the second-order algebraic diagrammatic construction ADC(2) method.^31−33^ The correlation–consistent valence double-ζ basis set with polarization functions on all atoms (cc-pVDZ) was used.^34^ For comparison, the vertical absorption and emission of few selected systems were also computed with the aid of the CC2 method, a simplified version of the coupled-clusters theory with single and double replacements, using the cc-pVDZ basis set.^31,33^ Additional single-point calculations using the more accurate scaled opposite spin (SOS-CC2) method were also performed.^35,36^

**Figure 5:**
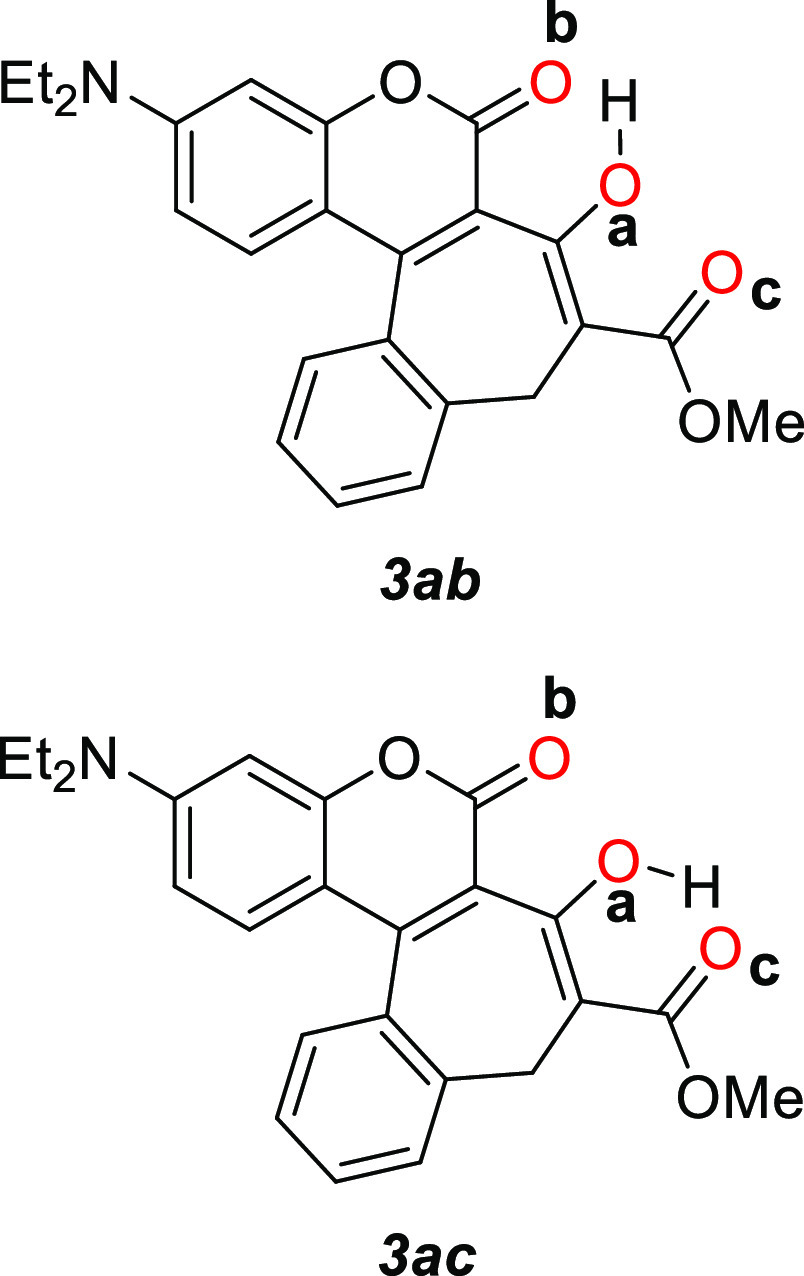
Chemical structure of the rotamers **3*****ab*** and **3*****ac*** showing the labels used for the oxygen atoms.

Coumarins **3**, **4**, **9**, **13**, **14**, and **15** are predicted to be out of the plane, with a torsion angle between coumarin and benzene ring around 50°. ^1^H NMR studies have proven that they exist exclusively in the enol forms. For dyes **3**, **4**, and **9**, two rotational isomers are expected to be thermally populated in the ground state (Table S2). Both rotamers are expected to have the labile proton attached to the ***a*** oxygen (Figure 5), with the ***ac*** structure being more stable and less polarized than ***ab*** by ∼0.3 eV and 2 D.

Excitation energies for these derivatives were evaluated at the ADC(2)/cc-pVDZ level of theory using the ground-state equilibrium geometry obtained at the MP2 level with the cc-pVDZ basis set. Inspection of these explorations presented in Table S3 of the ESI leads to the conclusion that the excitation energies computed for ***ab*** are around 0.3 eV lower than the respective ***ac*** isomers for **3**, **4**, and **9**. All derivatives exhibit strong dipole allowed absorption transitions to the lowest excited singlet state of the vertical excitation energy close to 3 eV. These electronic transitions are of ππ* nature, with transition dipole moments ranging from 13 to 17 D, involving molecular orbitals delocalized over the whole coumarin derivative structure.

Electronic transitions computed at the same level for compounds **13**–**15** exhibit two vertical excited states in close energy, a weakly absorbing ^1^LE state of ππ* character around 3 eV, followed by a significantly more polarized and strongly absorbing bright ^1^CT state (also ππ*), located vertically around 3.2 eV for **13**. Thus, we expect that a larger stabilization of the ^1^CT state in polar solvents would result in enhancement of both the fluorescence and solvatochromism for these compounds. This feature was observed experimentally. Excitation energies of **14** were also computed within CC2 and SOS-CC2 approximations. The latter suggests a significant blueshift of the 0–0 transition energy, as previously observed by Hellweg and co-workers for a series of organic molecules.^37^

Geometry optimization of the *S*_1_ state of **3**, **4**, and **9** resulted in stable ***ac*** structures, which are predicted to fluoresce at about 1.5 eV at the ADC(2) level of theory. As previously observed for related chromophores,^36,38−40^ ADC(2) tends to underestimate the transition energies below 2 eV; thus, to obtain more accurate emission energies, additional calculations were performed within the scaled opposite spin (SOS-CC2) method (Table S4). Within this approximation, emission energies are predicted to be around 2.3 eV.

To investigate the occurrence of excited-state intramolecular proton transfer^41^ in this series of compounds, potential energy (PE) profiles along the proton transfer reaction coordinate of the respective ***ac*** isomer were performed for selected compounds (**3**, **13**, and **14**). The results are presented in Figures S7 and S8. PE profiles were optimized in the excited singlet state (*S*_1_), and the vertical energy of the ground state was computed along such determined minimum energy reaction paths. It can be noticed that, upon inspection of Figure S7, for **3**, the minimum energy profile of the singlet locally excited (^1^LE) state, which involves a local excitation within the coumarin core, is an increasing function of the O–H bond distance, with a barrier less than 0.3 eV. However, for the ***ac*** isomer, the proton transfer is a nonadiabatic process, most probably induced by charge transfer between the cycloheptatrienol and carbonyl moieties. The electronic structures of the ^1^LE and ^1^CT states determined at the same value of the proton transfer coordinate (O–H = 1.3 Å) are shown in Table S5. The proton transfer barrier was also estimated for the coumarin moiety (***ab*** isomer) of compound **3** (Figure S8). In this case, the conical intersection to the ground state would be reached in a barrierless fashion. Summarizing, **3*****ab*** would be nonemissive, with ESIPT predicted to be its main deactivation channel, while the emission observed experimentally can be assigned to the population of the isomer **3*****ac*** in the excited state.

The possibility of the formation of ring-opened photoproducts for 3-amino-7-hydroxybenzo[3,4]cyclohepta[1,2-*c*]chromen-6-ones (Figure S4) was also considered (Figure S8). The computed PE profile, however, predicts a barrier of 0.4 eV hindering access to ring-open isomers of **3***ac* species.

**Figure 6:**
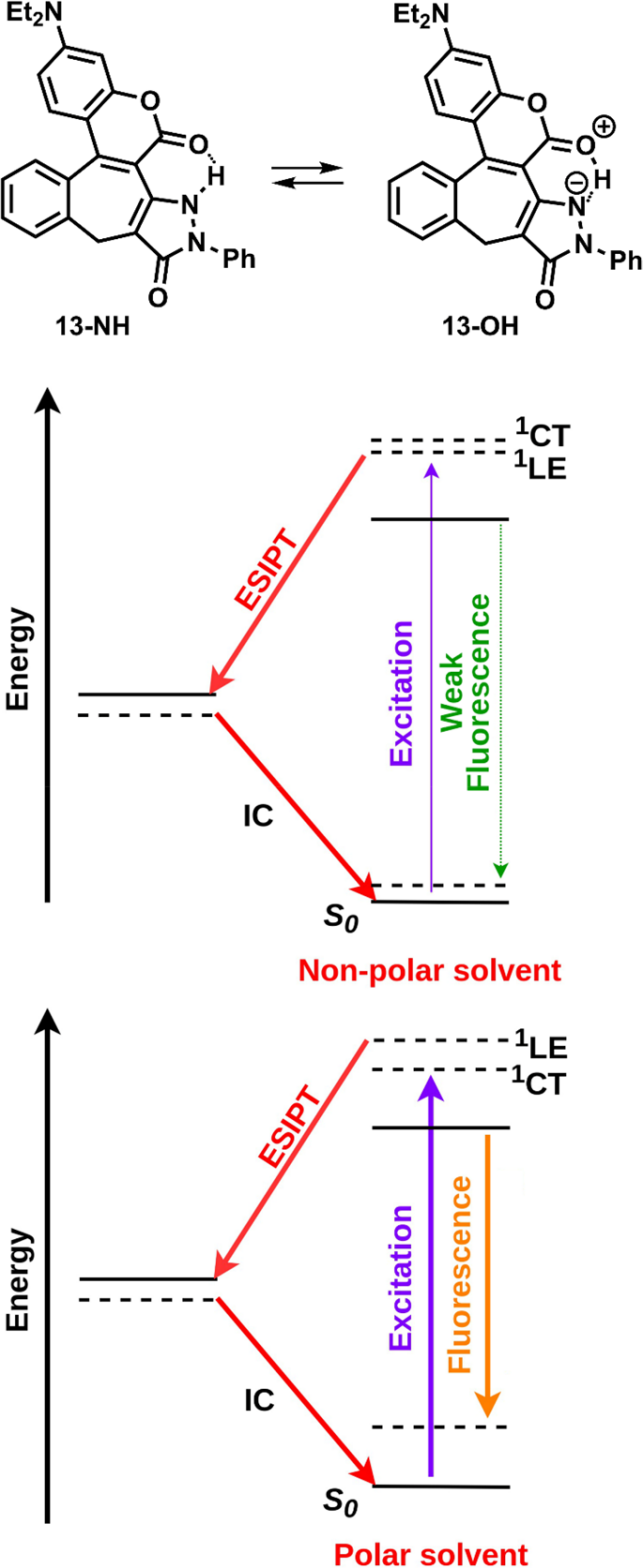
Summary of active electronic states and photophysical processes for compounds **13**, **14**, and **15** in polar and nonpolar solvents obtained at the MP2/ADC(2)/cc-pVDZ level of theory. Solid levels denote the adiabatic (optimized) energy of a given state, and dashed levels denote the vertical energy of the absorbing/fluorescing state computed at the optimized geometry of the respective states.

Excited-state geometry optimization of **13**–**15** resulted in a spontaneous ESIPT reaction involving proton transfer from the N–H of the pyrazolone moiety to the carbonyl group of the coumarin (Figure S7 and Figure 6). As a result, the *S*_1_–*S*_0_ energy gap is drastically reduced, suggesting that the formed **13-OH** species would be nonemissive (Figure S6). However, the emission observed experimentally for these compounds is most probably due to the significant stabilization of the *S*_2_ (^1^CT) state (Figure S10) in the presence of polar/polarizable solvents (acetonitrile and dichloromethane/toluene). The effect of solvent on the emission from the ^1^CT state was evaluated considering two explicit acetonitrile molecules in proximity to the proton transfer site of the compound **14**. The resultant microsolvated structure is predicted to emit at lower energy (2.4 eV) than the nonsolvated species (2.9 eV). This trend is consistent with the solvatochromism observed experimentally.

In this contribution, we have made three sets of dyes with remarkably different photophysical properties. For compounds **3** and **9**, the isomer called ***ac*** is predicted to be more stable than ***ab***; however, this energy difference is rather small. Thus, the fully allowed optical transitions of both species are expected. Only one emissive species was found, ***ac***, which was predicted to fluoresce from the lowest excited ππ* state. The ESIPT reaction path computed for **3*****ab*** suggests that the conical intersection would be reached in a barrierless fashion, while the ESIPT of **3*****ac*** is predicted to be nonadiabatic. In compounds **13**–**15**, there are two vertical states to be considered, the weakly absorbing ^1^LE state followed by a bright ^1^CT state, which strongly absorbs at slightly (0.2 eV) higher energy. The order of these states strongly depends on the polarity of the solvent since ^1^CT is significantly more polarized than ^1^LE. These observations are summarized in Figure 6.

Due to solubility issues, only dioxane was used as a solvent for **5** and **11**. For these molecules, the absorption is significantly lower than the others. Since a long tail and two distinguished bands can be observed for the absorption and emission spectra of these compounds, respectively, two possible structures were considered, namely, ***ab*** and ***openab***, the latter being a rotamer by way of the chlorophenyl OH moiety. In both compounds, the phenyl and naphthyl groups bonded to the cycloheptatrienol moieties are 90° twisted with respect to the coumarin rings. This leads to weak coupling between the moieties, clearly visible in the molecular orbitals of Table S3b, and results in very low oscillator strength (Table S3a), typical for nonemissive compounds. The complexation with two solvent molecules, a polarizable 1,4-dioxane, leads to electronic density delocalization over the whole compound and affects the optical transition moment by a small amount. Indeed, both compounds are weakly absorbing and the lowest molar absorptivity among all molecules studied in this work (Table 2). The twist around the C=C double bond provides an efficient channel for radiationless deactivation, which dominates the radiative transition. In fact, the lowest fluorescence quantum yields are observed for compounds **5** and **11** (Table 2).

## Conclusion

Our experiments provide a proof-of-concept demonstration that highly curved, helical coumarins are formed via the Michael addition of aminophenols to cyclic α,β-unsaturated esters. The mechanism consists of the Michael addition, followed by transesterification and [1,5] sigmatropic rearrangement. The net result is that the initially formed C–C single bond becomes a C–C double bond, which leads to the formation of a coumarin ring system. Depending on the strength of the electron-donating group present on the phenol, [1,5] sigmatropic rearrangement occurs either spontaneously at 140 °C (under reaction conditions) or at 220 °C if a dialkylamino group is not present. The addition of phenols to α,β-unsaturated esters leading to coumarins catalyzed by Lewis acids seems to be a general process. The helical coumarins display moderate green emission with a moderate solvatofluorochromic effect and a small effect in absorption spectra. We report de-excitation pathways and mechanisms operating in the electronically excited states, relating the photophysical properties to the molecular structure of the compounds studied. The highly twisted structure of these helical coumarins results in weak electronic coupling that leads to low molar absorptivity in the UV. The less twisted structures of other compounds result in electronic coupling of moieties and strong absorption at a lower energy in the visible blue region. Efficient ESIPT in the case of pyrazolone–coumarins and double bond twist leads to internal conversion to the ground state, providing a nonradiative channel that competes with the radiative transitions in all compounds studied. Our findings hold the potential for an immediate impact on organic synthesis and construction of fluorescent probes.

## Experimental Section

### General Information

Dry dichloromethane was used for all syntheses. All reported NMR spectra (^1^H NMR and ^13^C NMR) were recorded using a Varian 500 or Bruker 500 spectrometer. Chemical shifts (δ ppm) were determined with TMS as the internal reference, and *J* values are given in hertz (Hz). High-resolution mass spectra (HRMS) were obtained via an electron ionization (EI) source and an EBE double-focusing geometry mass analyzer. Chromatography was performed on silica gel 60 (230–400 mesh), and thin-layer chromatography was performed on TLC plates (Merck, silica gel 60 F_254_).

#### Photophysical Measurements

Spectroscopic grade solvents were purchased from Sigma-Aldrich and used as obtained. For optical studies, solutions of molecules at low concentrations, about a few micromoles per liter, were used to avoid dimerization or reabsorption effects. Before measurements, solutions were bubbled with pure nitrogen gas for 20 min. All absorption and fluorescence spectra were taken at room temperature (21 °C). A PerkinElmer UV/vis spectrometer, model Lambda 35, was used for absorption spectra measurement. Fluorescence spectra were recorded with an FLS 1000 spectrofluorometer from Edinburgh Instruments and corrected for the spectral response sensitivity of the photodetector. Fluorescence quantum yields (FQY) of molecules in solvents at 21 °C were determined using 9,10-diphenylanthracene and quinine sulfate as FQY standards. Solutions of low absorbance (*A* < 0.1) were used to avoid reabsorption or concentration quenching. Corrections for the refractive index of solvents have been performed in the calculations of quantum yields.^42^ Molar absorptivity (absorption coefficient), ε, was calculated from the absorbance, *A*, of a solution of the given molar concentration, *c*, in a cuvette of length *l* with a well-known formula: *A* = *c* × ε × *l*. Fluorescence kinetics studies were performed with the “time-correlated” single-photon counting technique. Excitation pulses were provided by the second harmonic of a mode-locked Coherent Mira-HP picosecond laser pumped by a Verdi 18 laser. The original 76 MHz repetition rate of a Mira laser was reduced with the aid of APE Pulse Selector to 3.8 MHz. Fluorescence photons were detected with an HMP-100–50 hybrid detector and an SPC-150 module, both from Becker&Hickl GmbH. Fluorescence decays were analyzed with a deconvolution computer program, which uses a nonlinear least-squares procedure with the Marquardt method.^43^ A standard χ^2^ test was used along with residual and autocorrelation function plots to judge the quality of a fit. The estimated precision of the decay time determination was 10 ps.

### Synthesis

#### General Procedure for the Synthesis of Compound **3**

To the flask was added compound **1** (272 mg, 1 mmol), 3-(diethylamino)phenol (**2**) (332 mg, 2 mmol), and In(OTf)_3_ (11 mg, 0.02 mmol). The reaction mixture was stirred at 140 °C (oil bath) for 5 h. Then the mixture was cooled to room temperature, dissolved in a small amount of DCM, and purified by column chromatography (silica, hexane/AcOEt 2:1 as the eluent). Crystallization from MeOH afforded the product in analytical purity.

#### Compound **3**

Yellow precipitate. Yield: 0.206 g (51%). Mp: 165–168 °C. ^1^H NMR (CD_2_Cl_2_, 500 MHz): δ 12.16 (s, 1H), 7.50 (d, 1H, *J* = 7.7 Hz), 7.47–7.42 (td, 1H, *J* = 7.5, 1.2 Hz), 7.38–7.30 (m, 3H), 6.58–6.55 (m, 2H), 3.84 (s, 3H), 3.72 (d, 1H, *J* = 14.0 Hz), 3.45 (q, 4H, *J* = 7.2 Hz), 2.90 (d, 1H, *J* = 14.0 Hz), 1.23 (t, 6H, *J* = 7.2 Hz). ^13^C{^1^H} NMR (CD_2_Cl_2_, 125 MHz): δ 171.5, 164.8, 159.0, 156.8, 153.5, 151.7, 145.6, 131.4, 131.1, 131.0, 130.9, 126.8, 125.5, 112.6, 109.1, 107.4, 106.6, 97.5, 52.4, 45.2, 30.0, 12.6. HRMS (EI): *m*/*z* calcd for C_24_H_23_NO_5_, 405.1576 [*M*^·+^]; found, 405.1588.

#### General Procedure for the Synthesis of Compound **5**

To the flask were added compound **1** (272 mg, 1 mmol), 4-chlororesorcinol (**10**) (290 mg, 2 mmol), and In(OTf)_3_ (11 mg, 0.02 mmol). The reaction mixture was stirred at 120 °C (oil bath) for 1 h. Then the mixture was cooled to room temperature, and AcOEt was added. The precipitate was filtered and crystallized from MeOH to afford the product in analytical purity.

#### Compound **5**

Off-white precipitate. Yield: 0.206 g (54%). Mp: 204–205 °C. ^1^H NMR (DMSO-*d*_6_, 500 MHz): δ 12.21 (s, 1H), 10.78 (s, 1H), 8.41 (s, 1H), 7.73 (d, 1H, *J* = 8.0 Hz), 7.46 (t, 1H, *J* = 7.0 Hz), 7.36 (t, 1H, *J* = 7.5 Hz), 7.20 (s, 1H), 6.82 (s, 1H), 6.52 (d, 1H, *J* = 7.5 Hz), 4.34 (s, 1H), 3.83 (s, 3H). ^13^C{^1^H} NMR (DMSO-*d*_6_, 125 MHz): δ 165.6, 164.9, 164.8, 153.6, 149.6, 145.4, 143.6, 132.0, 131.6, 130.4, 130.3, 127.8, 126.6, 123.9, 116.6, 111.1, 104.5, 97.7, 52.6, 37.4. HRMS (EI): *m*/*z* calcd for C_20_H_13_ClO_6_, 384.0401 [*M*^·+^]; found, 384.0401.

#### General Procedure for the Synthesis of Compound **8**

To the solution of naphthalene-2,3-dicarbaldehyde (**6**) (1.104 g, 6 mmol) in toluene (100 mL) were added dimethyl-1,3-acetonedicarboxylate (**7**) (1 mL, 7 mmol), piperidine (0.1 mL, 1 μmol), and acetic acid (0.3 mL, 5 μmol). The reaction mixture was stirred at 130 °C (oil bath) for 12 h using a Dean–Stark apparatus. Then the mixture was cooled to room temperature, most of the toluene was removed under a vacuum, and the remaining precipitate was filtered and purified by column chromatography (silica, DCM/MeOH 99:1 as the eluent). Crystallization from AcOEt–cyclohexane afforded the product in analytical purity.

#### Compound **8**

Yellow precipitate. Yield: 1.040 g (54%). Mp: 212–213 °C. ^1^H NMR (CDCl_3_, 500 MHz): δ 8.304 (s, 2H), 8.298 (s, 2H), 8.03–7.97 (m, 2H), 7.72–7.67 (m, 2H), 3.94 (s, 6H). ^13^C{^1^H} NMR (CDCl_3_, 125 MHz): δ 184.2, 166.7, 142.8, 136.1, 134.4, 133.7, 130.4, 129.2, 128.5, 52.9. HRMS (EI): *m*/*z* calcd for C_19_H_14_O_5_, 322.0841 [*M*^·+^]; found, 322.0833.

#### General Procedure for the Synthesis of Compound **9**

To the flask was added compound **8** (161 mg, 0.5 mmol), 3-(diethylamino)phenol (**2**) (165 mg, 1 mmol) and In(OTf)_3_ (5 mg, 0.01 mmol). The reaction mixture was stirred at 140 °C (oil bath) for 1 h. Then the mixture was cooled to room temperature, dissolved in a small amount of DCM, and purified by column chromatography (silica, hexane/AcOEt 2:1 as the eluent). Crystallization from MeOH afforded the product in analytical purity.

#### Compound **9**

Yellow precipitate. Yield: 0.107 g (47%). Mp: 218–220 °C. ^1^H NMR (CD_2_Cl_2_, 500 MHz): δ 12.20 (s, 1H), 7.99 (s, 1H), 7.89 (t, 2H, *J* = 7.7 Hz), 7.77 (s, 1H), 7.58 (td, 1H, *J* = 8.0, 1.2 Hz), 7.51 (td, 1H, *J* = 8.0, 1.1 Hz), 7.36 (d, 1H, *J* = 9.2 Hz), 6.65–6.57 (m, 2H), 3.91 (d, 1H, *J* = 14.0 Hz), 3.85 (s, 3H), 3.46 (q, 4H, *J* = 7.1 Hz), 3.15 (d, 1H, *J* = 14.0 Hz), 1.24 (t, 6H, *J* = 7.1 Hz). ^13^C{^1^H} NMR (CD_2_Cl_2_, 125 MHz): δ 171.6, 165.0, 158.9, 157.0, 153.7, 151.5, 143.1, 134.7, 131.7, 131.0, 130.1, 128.8, 127.9, 127.5, 126.3, 124.2, 112.7, 109.6, 109.0, 108.2, 106.3, 98.0, 52.4, 45.5, 30.4, 12.6. HRMS (EI): *m*/*z* calcd for C_28_H_25_NO_5_, 455.1733 [*M*^·+^]; found, 455.1743.

#### General Procedure for the Synthesis of Compound **11**

To the flask were added compound **8** (161 mg, 0,5 mmol), 4-chlororesorcinol (**10**) (145 mg, 1 mmol), and In(OTf)_3_ (5 mg, 0.01 mmol). The reaction mixture was stirred at 120 °C (oil bath) for 1 h. Then, the mixture was cooled to room temperature, and AcOEt was added. The precipitate was filtered and crystallized from MeOH to afford the product in analytical purity.

#### Compound **11**

Yellow precipitate. Yield: 0.097 g (44%). Mp: 200–201 °C. ^1^H NMR (DMSO-*d*_6_, 500 MHz): δ 12.36 (bs, 1H), 10.80 (s, 1H), 8.55 (s, 1H), 8.38 (s, 1H), 8.02–7.96 (m, 1H), 7.89–7.84 (m, 1H), 7.59–7.52 (m, 2H), 7.27 (s, 1H), 7.00 (s, 1H), 6.89 (s, 1H), 4.69 (s, 1H), 3.88 (s, 3H). ^13^C{^1^H} NMR (DMSO-*d*_6_, 125 MHz): δ 166.2, 165.1, 165.0, 153.6, 149.7, 145.6, 141.7, 134.0, 131.2, 131.0, 130.5, 130.4, 128.2, 128.0, 127.7, 127.3, 126.6, 122.5, 116.6, 111.4, 104.6, 97.9, 52.6, 37.8. HRMS (EI): *m*/*z* calcd for C_24_H_15_ClO_6_, 434.0557 [*M*^·+^]; found, 434.0544.

#### General Procedure for the Synthesis of Compound **13**

To a dry Schlenk flask were added compound **3** (41 mg, 0.1 mmol), T_3_P in DCM (64 mg, 0.2 mmol), and dry toluene (5 mL) under argon. Phenylhydrazine (**12**) (0.02 mL, 0.2 mmol) was subsequently added, and the reaction mixture was refluxed (oil bath) for 24 h. Then the mixture was cooled to room temperature and evaporated. The crude product was purified by column chromatography (silica, DCM/MeOH 99:1 as the eluent). It was then crystallized from DCM–cyclohexane to afford the product in analytical purity.

#### Compound **13**

Yellow precipitate. Yield: 0.033 g (72%). Mp: 246–247 °C. ^1^H NMR (CD_2_Cl_2_, 500 MHz): δ 9.18 (bs, 1H), 7.78–7.74 (m, 2H), 7.51–7.38 (m, 6H), 7.31 (t, 1H, *J* = 7.4 Hz), 7.18 (t, 1H, *J* = 7.4 Hz), 6.67–6.61 (m, 2H), 3.86 (d, 1H, *J* = 14.4 Hz), 3.48 (q, 4H, *J* = 7.1 Hz), 3.25 (d, 1H, *J* = 14.4 Hz), 1.25 (t, 6H, *J* = 7.1 Hz). ^13^C{^1^H} NMR (CD_2_Cl_2_, 125 MHz): δ 161.7, 156.2, 154.8, 151.8, 145.6, 143.8, 138.1, 132.3, 132.0, 131.6, 131.2, 129.4, 128.6, 125.7, 125.2, 119.3, 114.6, 109.8, 109.1, 97.7, 45.3, 28.0, 12.6. HRMS (EI): *m*/*z* calcd for C_29_H_25_N_3_O_3_, 463.1896 [*M*^·+^]; found, 463.1898.

#### General Procedure for the Synthesis of Compound **4**

To the flask were added compound **1** (816 mg, 3 mmol), 8-hydroxyjulolidine (945 mg, 5 mmol), and In(OTf)_3_ (34 mg, 0.06 mmol). The reaction mixture was stirred at 140 °C (oil bath) for 5 h. Compound **4** was unstable and was used to the next step without purification

#### Compound **4**

Orange precipitate. Yield: 0.996 g (77%). The structure was confirmed by mass spectrometry analysis in low resolution. LRMS (EI): *m*/*z* calcd for C_26_H_23_NO_5_, 429.1576 [*M*^·+^]; found, 429.1.

Compounds **14** and **15** were synthesized according to the same procedure as compound **13**.

#### Compound **14**

Orange precipitate. Yield: 0.058 g (79%). Mp: 230 °C (decomp). ^1^H NMR (CD_2_Cl_2_, 500 MHz): δ 9.25 (bs, 1H), 7.78–7.74 (m, 2H), 7.50–7.38 (m, 5H), 7.31 (td, 1H, *J* = 8.0, 1.0 Hz), 7.18 (t, 1H, *J* = 7.5 Hz), 6.98 (s, 1H), 3.85 (d, 1H, *J* = 14.0 Hz), 3.39–3.29 (m, 4H), 3.24 (d, 1H, *J* = 14.0 Hz), 3.02–2.89 (m, 2H), 2.80–2.60 (m, 2H), 2.08–2.01 (m, 2H), 2.01–1.89 (m, 2H). ^13^C{^1^H} NMR (CD_2_Cl_2_, 125 MHz): δ 161.9, 161.8, 154.9, 151.1, 147.2, 146.0, 143.8, 138.1, 132.5, 132.3, 131.3, 129.3, 128.4, 127.1, 125.6, 125.1, 119.5, 119.3, 114.1, 108.8, 106.6, 105.5, 50.4, 49.9, 28.0, 28.0, 21.7, 20.9, 20.7. HRMS (EI): *m*/*z* calcd for C_31_H_25_N_3_O_3_, 487.1896 [*M*^·+^]; found, 487.1897.

#### Compound **15**

Yellow precipitate. Yield: 0.037 g (55%). Mp: 268 °C (decomp). ^1^H NMR (CD_2_Cl_2_, 500 MHz): δ 7.97 (s, 1H), 7.90–7.80 (m, 3H), 7.73 (d, 2H, *J* = 7.8 Hz), 7.55 (t, 1H, *J* = 7.0 Hz), 7.50 (t, 1H, *J* = 7.0 Hz), 7.44 (d, 1H, *J* = 8.9 Hz), 7.40 (t, 2H, *J* = 8.4 Hz), 7.18 (t, 1H, *J* = 7.4 Hz), 6.68–6.62 (m, 2H), 4.12 (d, 1H, *J* = 14.3 Hz), 3.53–3.43 (m, 5H), 1.26 (t, 6H, *J* = 7.1 Hz). ^13^C{^1^H} NMR (CD_2_Cl_2_, 125 MHz): δ 161.6, 156.4, 155.1, 151.9, 145.7, 141.6, 137.9, 135.0, 132.8, 131.3, 131.1, 130.8, 129.4, 128.7, 128.1, 127.5, 126.5, 125.4, 119.6, 110.0, 109.6, 97.8, 45.4, 28.4, 12.7. HRMS (EI): *m*/*z* calcd for C_33_H_27_N_3_O_3_, 513.2052 [*M*^·+^]; found, 513.2062.

#### General Procedure for the Synthesis of Compound **16**

Compound **5** (250 mg, 0.65 mmol) was added to a round-bottom flask and stirred at 230 °C (oil bath) for 2 h under a vacuum. Then the mixture was cooled to room temperature, and the product was purified by preparative chromatography (silica, DCM/MeOH 95:5 as the eluent). The precipitate was crystallized from MeOH to afford the product in analytical purity.

#### Compound **16**

Yellow precipitate. Yield: 0.164 g (66%). Mp: 225 °C (decomp). ^1^H NMR (DMSO-*d*_6_, 500 MHz): δ 11.80 (s, 1H), 7.61–7.51 (m, 2H), 7.45 (t, 1H, *J* = 7.6 Hz), 7.41 (d, 1H, *J* = 7.6 Hz), 7.37 (s, 1H), 7.00 (s, 1H), 3.82 (s, 3H), 3.65 (d, 1H, *J* = 14.2 Hz), 2.96 (d, 1H, *J* = 14.2 Hz). ^13^C{^1^H} NMR (DMSO-*d*_6_, 125 MHz): δ 170.0, 161.8, 157.3, 156.9, 153.3, 151.0, 145.2, 131.2, 130.5, 129.4, 129.3, 126.6, 125.7, 117.1, 115.6, 110.9, 107.3, 103.5, 52.4, 28.9. HRMS (EI): *m*/*z* calcd for C_20_H_13_ClO_6_, 384.0401 [*M*^·+^]; found, 384.0403.

## Supporting Information Available

X-ray crystallographic information; photophysical data; theoretical data; copies of ^1^H and ^13^C{^1^H} NMR data (PDF)

## Accession Codes

CCDC 2046086 contains the supplementary crystallographic data for this paper. These data can be obtained free of charge via www.ccdc.cam.ac.uk/data_request/cif, or by emailing data_request@ccdc.cam.ac.uk, or by contacting The Cambridge Crystallographic Data Centre, 12 Union Road, Cambridge CB2 1EZ, UK; fax: +44 1223 336033.

## References

[ref1] aMessaoudi S.; Brion J.-D.; Alami M. Palladium-Catalyzed Decarboxylative Coupling of Quinolinone-3-Carboxylic Acids and Related Heterocyclic Carboxylic Acids with (Hetero)aryl Halides. Org. Lett. 2012, 14, 1496–1499. 10.1021/ol300235k. 22381127

[ref2] aTsukamoto K.; Shinohara Y.; Iwasaki S.; Maeda H. A Coumarin-Based Fluorescent Probe for Hg^2+^ and Ag^+^ with an *N*’-Acetylthioureido Group as a Fluorescence Switch. Chem. Commun. 2011, 47, 5073–5075. 10.1039/c1cc10933b. 21431242

[ref3] aKim I.; Kim D.; Sambasivan S.; Ahn K. H. Synthesis of π-Extended Coumarins and Evaluation of Their Precursors as Reactive Fluorescent Probes for Mercury Ions. Asian J. Org. Chem. 2012, 1, 60–64. 10.1002/ajoc.201200034.

[ref4] aLiu Z.; Helander M. G.; Wang Z.; Lu Z. Efficient Single-Layer Organic Light-Emitting Diodes Based on C545T-Alq_3_ System. J. Phys. Chem. C 2010, 114, 11931–11935. 10.1021/jp101269r.

[ref5] aAdronov A.; Gilat S. L.; Fréchet J. M. J.; Ohta K.; Neuwahl F. V. R.; Fleming G. R. Light harvesting and energy transfer in laser- dye-labeled poly (aryl ether) dendrimers. J. Am. Chem. Soc. 2000, 122, 1175–1185. 10.1021/ja993272e.

[ref6] aMukhopadhyay A.; Hossen T.; Ghosh I.; Koner A. L.; Nau W. M.; Sahu K.; Moorthy J. N. Helicity-Dependent Regiodifferentiation in the Excited-State Quenching and Chiroptical Properties of Inward/Outward Helical Coumarins. Chem. - Eur. J. 2017, 23, 14797–14805. 10.1002/chem.201701787. 28792106

[ref7] aKim I.; Kim D.; Sambasivan S.; Ahn K. H. Synthesis of π-Extended Coumarins and Evaluation of Their Precursors as Reactive Fluorescent Probes for Mercury Ions. Asian J. Org. Chem. 2012, 1, 60–64. 10.1002/ajoc.201200034.

[ref8] aWecławski M. K.; Tasior M.; Hammann T.; Cywiński P. J.; Gryko D. T. From π-Expanded Coumarins to π-Expanded Pentacenes. Chem. Commun. 2014, 50, 9105–9108. 10.1039/C4CC03078H. 24985198

[ref9] Tasior M.; Poronik Y. M.; Vakuliuk O.; Sadowski B.; Karczewski M.; Gryko D. T. V-Shaped Bis-Coumarins: Synyhesis and Optical Properties. J. Org. Chem. 2014, 79, 8723–8732. 10.1021/jo501565r. 25133521

[ref10] aWang G.; Liu Y.; Zhang L.; An L.; Chen R.; Liu Y.; Luo Q.; Li Y.; Wang H.; Xue Y. Computational Study on the Antioxidant Property of Coumarin-Fused Coumarins. Food Chem. 2020, 304, 125446 10.1016/j.foodchem.2019.125446. 31491715

[ref11] aShao Z.; Xu L.; Wang L.; Wei H.; Xiao J. Catalyst – Free Tandem Michael Addition/Decarboxylation of (Thio)coumarin-3-carboxylic Acids with Indoles: Facile Synthesis of Indole-3-substituted 3,4-dihydro(thio)coumarins. Org. Biomol. Chem. 2014, 12, 2185–2188. 10.1039/C3OB42582G. 24589942

[ref12] aBu F.; Duan R.; Xie Y.; Yi Y.; Peng Q.; Hu R.; Qin A.; Zhao Z.; Tang B. Z. Unusual Aggregation-Induced Emission of a Coumarin Derivative as a Result of the Restriction of an Intramolecular Twisting Motion. Angew. Chem., Int. Ed. 2015, 54, 14492–14497. 10.1002/anie.201506782. 26439884

[ref13] Gawaskar S.; Schepmann D.; Bonifazi A.; Wünsch B. Synthesis, GluN2B Affinity and Selectivity of Benzo[7]annulen-7-amines. Bioorg. Med. Chem. 2014, 22, 6638–6646. 10.1016/j.bmc.2014.10.004. 25458498

[ref14] aGajewski J. J. Hydrocarbon Thermal Isomerizations; Academic Press: NY, 1981.

[ref15] Moss S.; King B. T.; de Meijere A.; Kozhushkov S. I.; Eaton P. E.; Michl J. LiCB_11_Me_12_: A Catalyst for Pericyclic Rearrangements. Org. Lett. 2001, 3, 2375–2377. 10.1021/ol0161864. 11463320

[ref16] Woodward R. B.; Hoffmann R. The Conservation of Orbital Symmetry; Academic Press: NY, 1970; pp 114–140.

[ref17] Hess B. A. Jr.; Baldwin J. E. [1,5] Sigmatropic Hydrogen Shifts in Cyclic 1,3-Dienes. J. Org. Chem. 2002, 67, 6025–6033. 10.1021/jo025917q. 12182638

[ref18] aWróbel Z.; Kwast A. Simple Synthesis of *N*-Aryl-2-Nitrosoanilines in the Reaction of Nitroarenes with Aniline Anion Derivatives. Synthesis 2010, 22, 3865–3872. 10.1055/s-0030-1258230.

[ref19] aZhang X.-S.; Li Z.-W.; Shi Z.-J. Palladium-Catalyzed Base-Accelerated Direct C-H Bond Alkenylation of Phenols to Synthesize Coumarin Derivatives. Org. Chem. Front. 2014, 1, 44–49. 10.1039/C3QO00010A.

[ref20] Mallouli A.; Lepage Y. Convenient Syntheses of Naphthalene,- Anthracene,- and Naphthacene-2,3-dicarboxaldehydes. Synthesis 1980, 1980, 689 10.1055/s-1980-29170.

[ref21] Alkiş M.; Pekyilmaz D.; Yalçin E.; Aydiner B.; Dede Y.; Seferoğlu Z. H-Bond Stabilization of a Tautomeric Coumarin-Pyrazole-Pyridine Triad Generates a PET Driven, Reversible and Reusable Fluorescent Chemosensor for Anion Detection. Dyes Pigm. 2017, 141, 493–500. 10.1016/j.dyepig.2017.03.011.

[ref22] Seydimemet M.; Ablajan K.; Hamdulla M.; Li W.; Omar A.; Obul M. L-Proline Catalyzed Four-Component One-Pot Synthesis of Coumarin-Containing Dihydropyrano[2,3-*c*]pyrazoles Under Ultrasonic Irradiation. Tetrahedron 2016, 72, 7599–7605. 10.1016/j.tet.2016.10.016.

[ref23] Pandit R. P.; Lee Y. R. Construction of Multifunctionalized Azopyrazoles by Silver-Catalyzed Cascade Reaction of Diazo Compounds. Adv. Synth. Catal. 2015, 357, 2657–2664. 10.1002/adsc.201500197.

[ref24] aKagaku O.; Kaisha K. Patent Application US 1997 /005639776A.

[ref25] Desroses M.; Jacques-Cordonnier M.-C.; Llona-Minguez S.; Jacques S.; Koolmeister T.; Helleday T.; Scobie M. A Convenient Microwave-Assisted Propylphosphonic Anhydride (T_3_P) Mediated One-Pot Pyrazolone Synthesis. Eur. J. Org. Chem. 2013, 2013, 5879–5885. 10.1002/ejoc.201300380.

[ref26] aRtishchev N. I.; Nosova G. I.; Solovskaya N. A.; Lukyaashina V. A.; Galaktionova E. F.; Kudryavtsev V. V. Photosensitization of Conjugated Bischalcone Derivatives. New T-Sensitizers. Russ. J. Gen. Chem. 2002, 72, 1942–1950. 10.1023/A:1023471514381.

[ref27] Vitorio F.; Pereira T. M.; Castro R. N.; Guedes G. P.; Graebin C. S.; Kummerle A. E. Synthesis and Mechanism of Novel Fluorescent Coumarin-Dihydropyrimidinone Dyads Obtained by the Biginelli Multicomponent Reaction. New J. Chem. 2015, 39, 2323–2332. 10.1039/C4NJ02155J.

[ref28] Babür B.; Seferoğlu N.; Seferoğlu Z. A Ratiometric Fluorescence Chemosensor Based on a Coumarin-Pyrazolone Hybrid: The Synthesis and an Investigation of the Photophysical, Tautomeric and Anion Binding Properties by Spectroscopic Techniques and DFT Calculations. Tetrahedron Lett. 2015, 56, 2149–2154. 10.1016/j.tetlet.2015.03.014.

[ref29] aUchiyama S.; Takehira K.; Yoshihara T.; Tobita S.; Ohwada T. Environment-Sensitive Fluorophore Emitting in Protic Environments. Org. Lett. 2006, 8, 5869–5872. 10.1021/ol062490r. 17134293

[ref30] Weigend F.; Häser M. RI-MP2: First Derivatives and Global Consistency. Theor. Chem. Acc. 1997, 97, 331–340. 10.1007/s002140050269.

[ref31] Schirmer J. Beyond the Random-Phase Approximation: A New Approximation Scheme for the Polarization Propagator. Phys. Rev. A: At., Mol., Opt. Phys. 1982, 26, 2395–2416. 10.1103/PhysRevA.26.2395.

[ref32] Trofimov B.; Schirmer J. An Efficient Polarization Propagator Approach to Valence Electron Excitation Spectra. J. Phys. B: At., Mol. Opt. Phys. 1995, 28, 2299–2324. 10.1088/0953-4075/28/12/003.

[ref33] Hättig Ch Structure Optimizations for Excited States with Correlated Second-Order Methods: CC2 and ADC(2). Adv. Quantum Chem. 2005, 50, 37–60. 10.1016/S0065-3276(05)50003-0.

[ref34] Dunning T. H. Gaussian Basis Sets for Use in Correlated Molecular Calculations. I. The Atoms Boron Through Neon and Hydrogen. J. Chem. Phys. 1989, 90, 1007–1023. 10.1063/1.456153.

[ref35] Christiansen O.; Koch H.; Jørgensen P. The Second-Order Approximate Coupled Cluster Singles and Doubles Model CC2. Chem. Phys. Lett. 1995, 243, 409–418. 10.1016/0009-2614(95)00841-Q.

[ref36] Jung Y.; Lochan R. C.; Dutoi A. D.; Head-Gordon M. Scaled Opposite-Spin Second Order Moller-Plesset Correlation Energy: An Economical Electronic Structure Method. J. Chem. Phys. 2004, 121, 9793–9802. 10.1063/1.1809602. 15549852

[ref37] Hellweg A.; Grün S. A.; Hattig C. Benchmarking the Performance of Spin-Component Scaled CC2 in Ground and Electronically Excited States. Phys. Chem. Chem. Phys. 2008, 10, 4119–4127. 10.1039/b803727b. 18612515

[ref38] Jacquemin D.; Duchemin I.; Blasé X. 0–0 Energies Using Hybrid Schemes: Benchmarks of TD-DFT, CIS(D), ADC(2), CC2 and BSE/*GW* Formalisms for 80 Real-Life Compounds. J. Chem. Theory Comput. 2015, 11, 5340–5359. 10.1021/acs.jctc.5b00619. 26574326 PMC4642227

[ref39] Vérité P. M.; Hédé S.; Jacquemin D. A Theoretical Elucidation of the Mechanism of Tuneable Fluorescence in a Full-Colour Emissive ESIPT Dye. Phys. Chem. Chem. Phys. 2019, 21, 17400–17409. 10.1039/C9CP03759D. 31359018

[ref40] Barboza C. A.; Gawrys P.; Banasiewicz M.; Suwinska K.; Sobolewski A. L. Photophysical Transformations Induced by Chemical Substitution to Salicylaldimines. Phys. Chem. Chem. Phys. 2020, 22, 6698–6705. 10.1039/D0CP00110D. 32162638

[ref41] aKwon J. E.; Park S. Y. Advanced Organic Optoelectronic Materials: Harnessing Excited-State Intramolecular Proton Transfer (ESIPT) Process. Adv. Mater. 2011, 23, 3615–3642. 10.1002/adma.201102046. 21780312

[ref42] Birks J. B. Photophysics of Aromatic Molecules; Wiley: London, 1979; pp 97–100.

[ref43] Demas J. N. Excited State Lifetime Measurements; Academic Press: NY, 1983; pp 89–92.

