# Supplementary material for: Polarized Helical Coumarins: [1,5] Sigmatropic Rearrangement and Excited-State Intramolecular Proton Transfer
Source: J Org Chem. 2021 Apr 8;86(9):6148–59. doi: 10.1021/acs.joc.0c02978 (PMC8154611; doi:10.1021/acs.joc.0c02978)
Supplement: Supplementary file 1 — jo0c02978_si_001.pdf [file jo0c02978_si_001.pdf]

Supporting Information for

Polarized Helical Coumarins: [1,5] Sigmatropic Rearrangement and Excited-State Intramolecular Proton Transfer

Łukasz Kielesiński, Olaf W. Morawski, Cristina A. Barboza and Daniel T. Gryko**

Table of Contents

1. Crystallographic data. S3
2. Spectroscopic data. S6
3. Computational results. S7
4. NMR Spectra. S22
5. Cartesian coordinates. S31

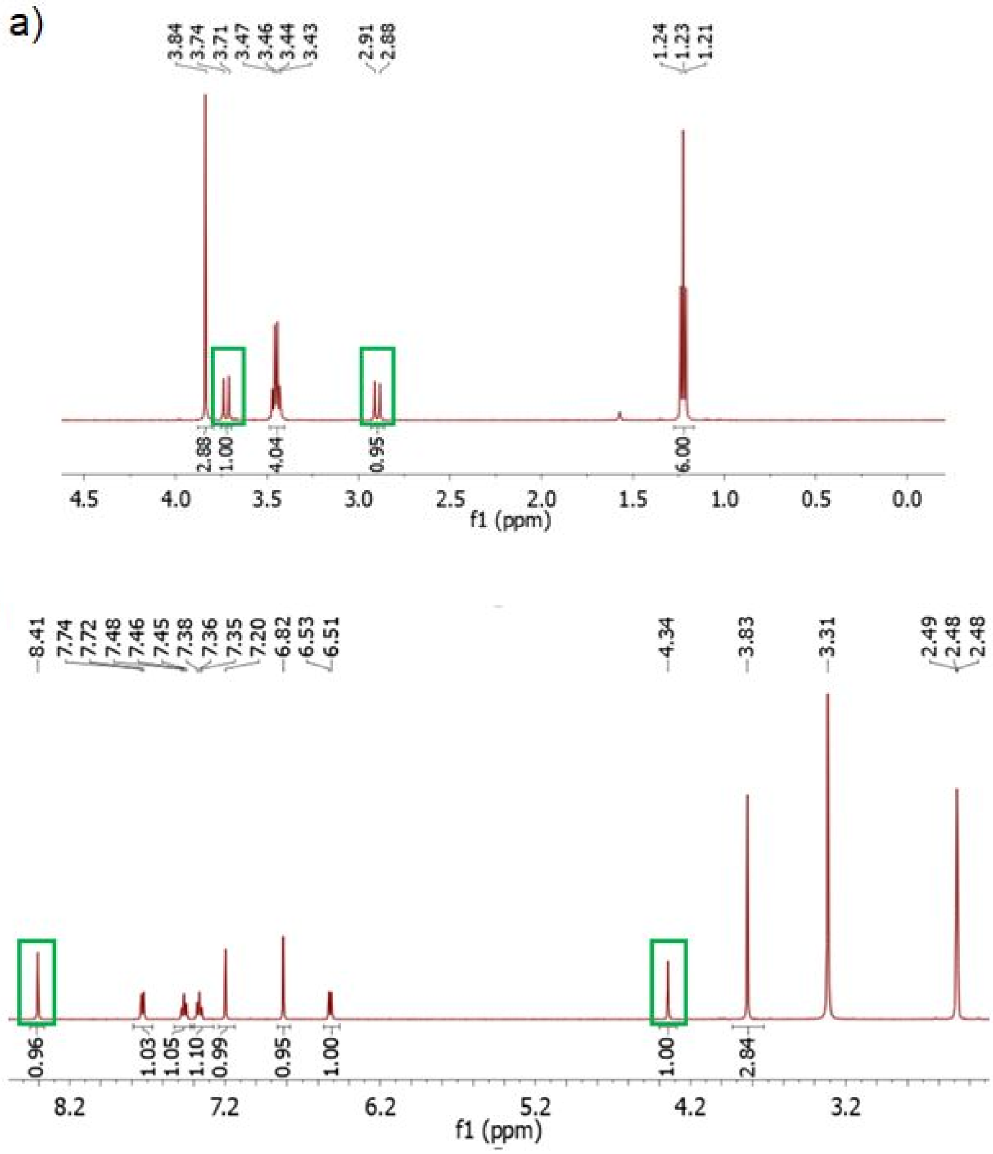

Figure S1. a) The ^{1}H NMR spectrum of compound **3** in CD_2Cl_2 - aliphatic region. The green frames indicate proton signals originating from the methylene bridge in the seven membered ring; b) The ^{1}H NMR spectrum of compound **5** in DMSO-d_6. The green frames indicate signals assigned to the double bond localized in the seven membered ring and from the coumarin pyran ring.

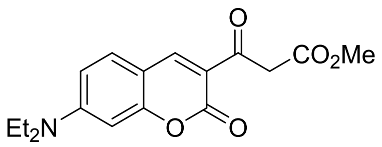

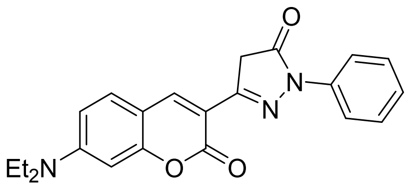

Figure S2. Structures of keto-coumarin derivatives (**Cum1** and **Cum2**).

1. Crystallographic data.

Table S1. Crystallographic data for compound **15**.

**Chemical formula**	$C_{33}H_{27}N_3O_3$	
**Formula weight**	513.58 g/mol	
**Temperature**	100 K	
**Wavelength**	1.54184 Å	
**Crystal size**	1.0 x 0.1 x 0.03 mm	
**Crystal habit**	yellow plate	
**Crystal system**	monoclinic	
**Space group**	$P2_1/c$	
**Unit cell dimensions**	a = 12.8454(2) Å	α = 90°
	b = 20.3341(2) Å	β = 97.221(1)°
	c = 9.6521(1) Å	γ = 90°
**Volume**	2501.13(5) Å^3	
**Z**	4	
**Diffractometer**	Agilent Supernova	
**Radiation source**	CuK_α	
**Reflections collected**	25904	
**Independent reflections**	4740 [R(int) = 0.0388]	
**Tmin, Tmax**	0.601, 1.000	
**Absorption correction**	multi-scan	
**Refinement method**	Full-matrix least-squares on F^2	
**Restraints / parameters**	0 / 286	
**Goodness-of-fit on F^2**	1.037	
**Final R indices**	$[F^2 > 2\sigma(F^2)]$	R1 = 0.0438, wR2 = 0.1203
	all data	R1 = 0.0481, wR2 = 0.1248

Single crystal X-ray diffraction measurements were carried out on a Agilent Supernova diffractometer, at 100 K with Cu Ka radiation (1.54184 Å). Yellow crystal of approximate dimensions 1.0 x 0.1 x 0.03 mm (plate) was used. The data reduction was made by using CrysAlisPRO [1] software. The structures were solved by direct methods and refined on F^2 by full-matrix least-squares by using SHELXS97 and SHELXL97 [2]. All non-hydrogen atoms were refined as anisotropic while hydrogen atoms were placed in calculated positions, and refined in riding mode.

[1] Agilent *CrysAlis PRO*. Agilent Technologies, Yarnton, England, **2011**.

[2] Sheldrick, G. M. *Acta Cryst*., **2008**, A64 112-122.

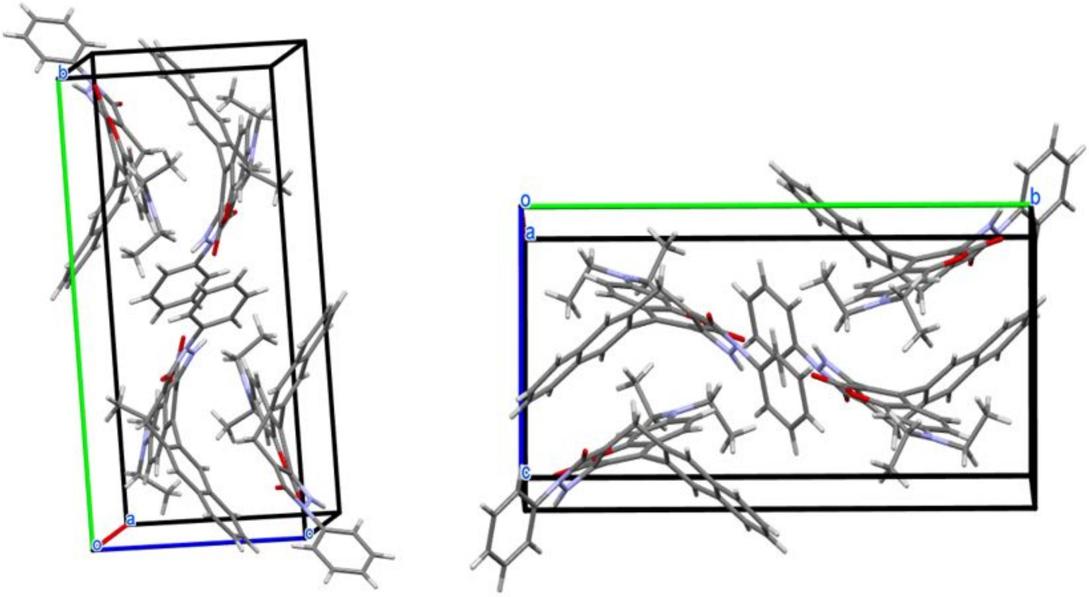

Figure S3. Packing diagram of crystals in elementary cell of compound **15**.

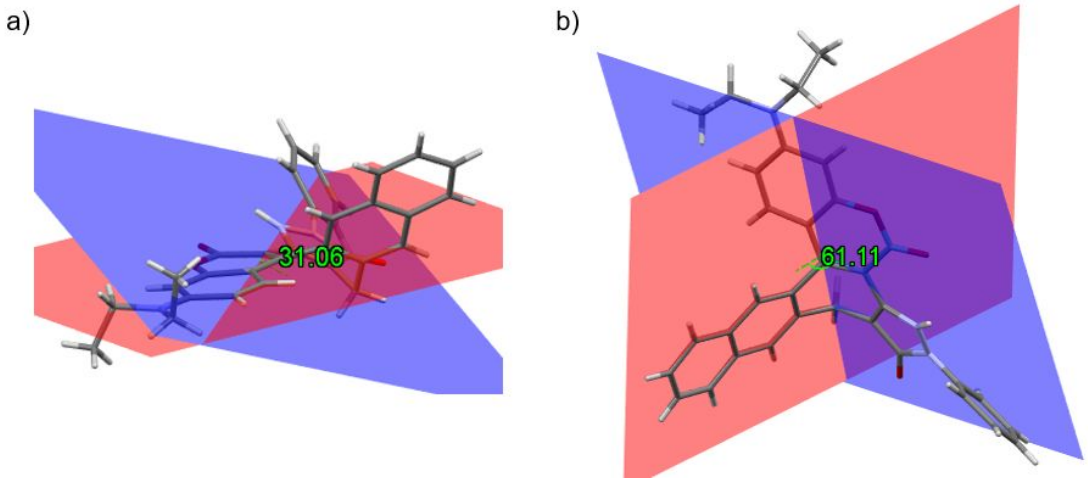

c)

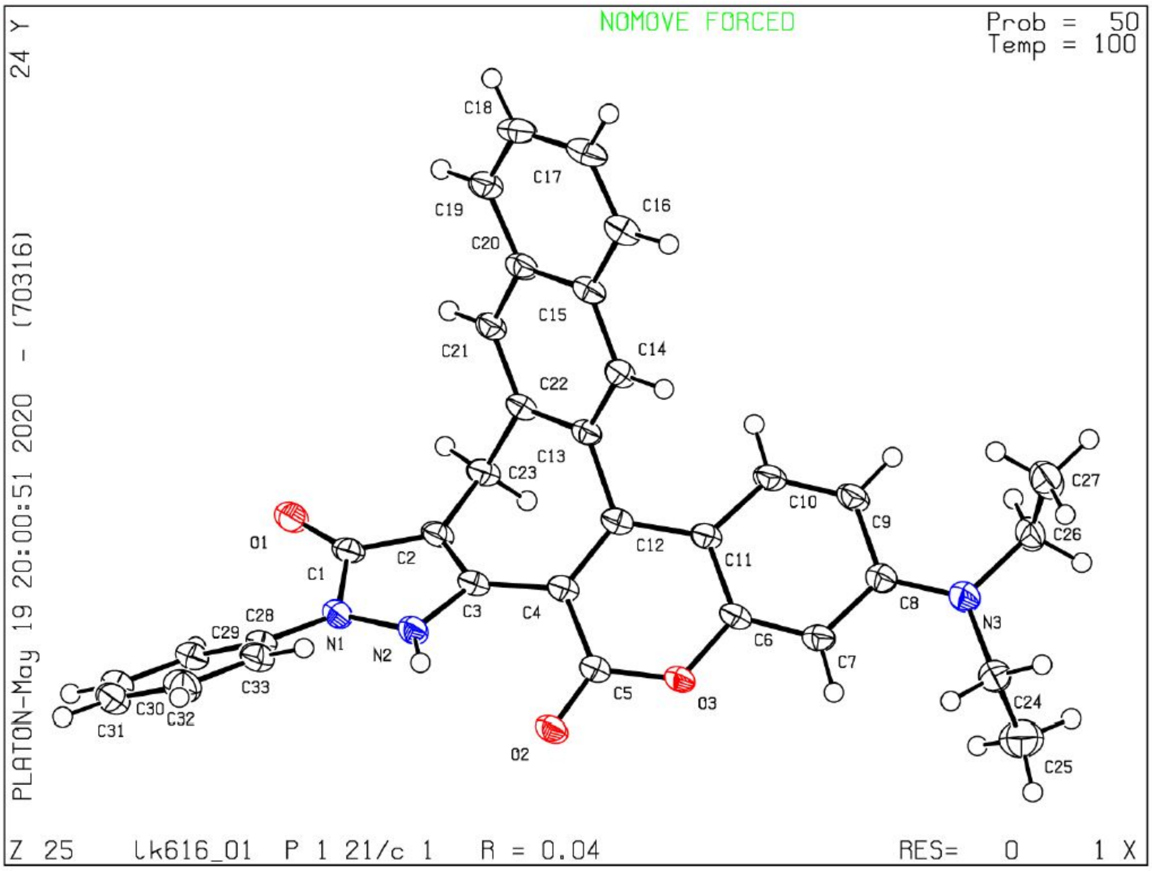

Figure S4. a) The plane and the value of dihedral angle between coumarin moiety and pyrazolone ring, b) the value of twist angle of seven membered ring in compound **15**, c) thermal ellipsoid plot for compound **15** with 50% probability level of ellipsoids contour.

2. Spectroscopic data.

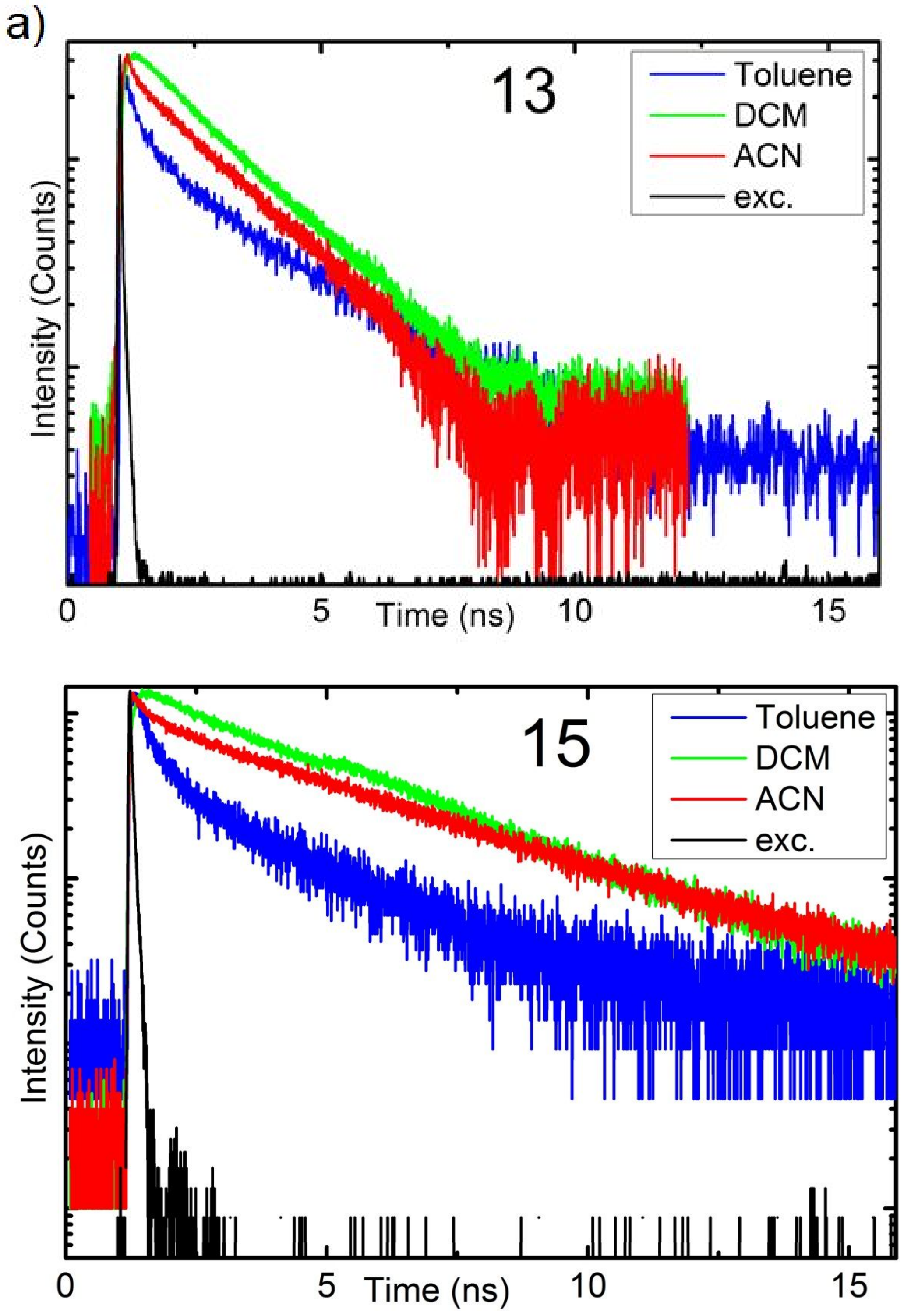

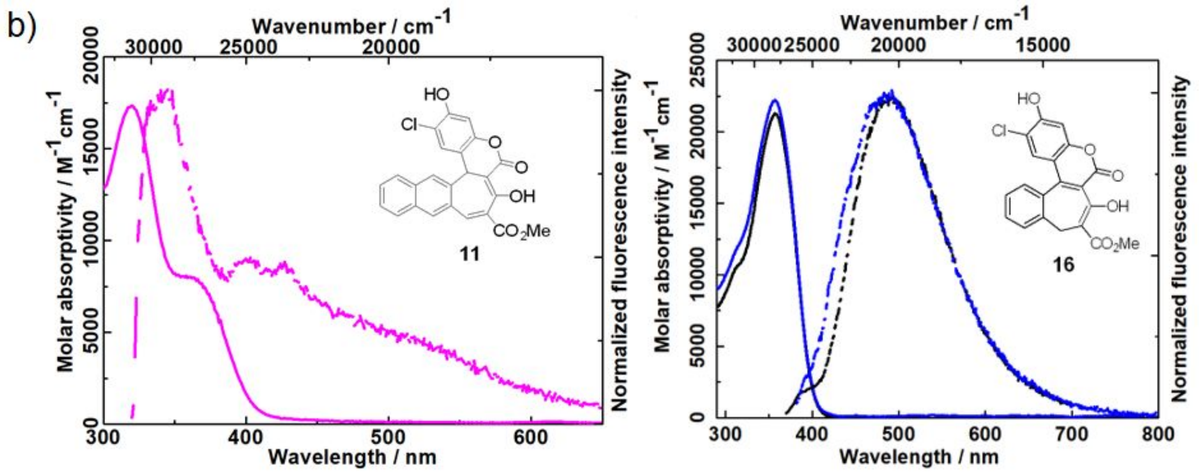

Figure S5. a) Semilogarithmic plot of fluorescence decays of compounds **13** and **15** recorded at 21 ^{o}C. Excitation pulse 394 nm (black line) and fluorescence decays observed at 500 nm in toluene (blue), DCM (green) and ACN (red). Temporal resolution 4.07 ps/channel and 12.2 ps/channel (compound **13** in DCM and ACN), b) Absorption (solid line) and emission (dashed line) spectra of compounds **11** (excited at 290 nm), **16** (excited at 340 nm) in toluene (black), DCM (blue) and dioxane (magenta).

3. Computational results.

Compounds 5 and* 11.** Optimized structures of **5** and **11** obtained at the MP2/cc-pVDZ level of theory are significantly different compared to the other coumarin derivatives due to the presence of a proton in the 4-position of the lactone ring, which seems to favor the C_1-C_2-C_3-O_4 twist by ~90° in the absence of explicit solvent molecules. This makes the phenyl and naphthyl groups bonded to the cycloheptatrienol moieties almost perpendicular to the coumarin fragment. For compound **5**, the ***ab isomer is around 0.3 eV more stable than ***ac*** computed in the gas phase. Besides, for the isomer ***ab*** an extra ground state structure was found, called ***open ab***, that could be formed due to the rotation of the chlorophenyl OH moiety, being predicted to be 0.1 eV less stable than the respective ***ab*** species.

Due to the significantly lower absorbance and intensity of fluorescence spectra of **5** and **11** measured in a moderately polarizable solvent such as dioxane, intermolecular interactions were considered and theoretically evaluated at the MP2/cc-pVDZ level of theory. For compound **5**, three structures were initially optimized for the more stable conformer **5 *ab***: *i.* closed shell anionic deprotonated, *ii.* neutral complex with one solvent molecule near to the –OH group attached to the coumarin [5 – (1,4-dioxane)], and *iii.* the open shell [5^{-} ··· (1,4-dioxane)–H^{+}]. The latter was found to be unstable. There is no evidence of deprotonation in the ground state

of **5**. Thus, the two species which are expected to be populated for compound **5** in the ground state are ***ab*** and ***open ab***. The influence of the solvent over the proton transfer of ***ab*** and ***open ab*** was evaluated by placing two dioxane molecules on top and below the O···H–O moiety (Table S2). Within this approximation the ***open ab + 2 dioxane*** structure is less stable than ***ab + 2 dioxane*** by less than 0.1 eV. However, the important distinction between the structures obtained in the gas phase and those using explicit dioxane molecules is that the solvent molecules hinder the C_1-C_2-C_3-O_4 twist, keeping the proton transfer site coplanar as can be seen in Figure S5. Similar results were obtained for compound **11**.

In agreement with absorption spectra measurements, the computed oscillator strengths for vertical excitations of compounds **5** are much lower (less than 0.1) than the other coumarin derivatives. They also have an increased charge transfer character, from the coumarin to the carboxyl group (Table S4). A broad absorption band is observed for this compound at 295 nm in dioxane (Figure 3) which may be related to the population of more than one absorbing species in the ground state, namely ***ab*** and ***open ab***. Computed vertical excitation energies at the ADC(2)/cc-pVDZ level of theory predict that both species would be almost degenerate. Excitation energies were also computed for ***ab + 2 dioxane*** and ***open ab + 2 dioxane***. The presence of explicit solvent molecules decreases the respective excitation energies by 0.2 eV. The same trend was observed for the compound **11**.

The presence of adding one extra phenyl ring to the structure of **11** decreases the excitation energy by ~0.3 eV respective to **5**. However, excitation energies of ***ab + 2 dioxane*** and ***open ab + 2 dioxane*** are predicted to be almost degenerate, around 3.6 eV for compound **11**. Experimentally, a redshift of the most intense absorption band (~320 nm) and the appearance of a shoulder at a longer wavelength is observed (Figure 3 and Figure S5b). Despite the similarity of absorption bands measured for compounds **5** and **11**, the latter has electronic transitions of ^{1}LE type, involving mostly molecular orbitals located on the naphthalene and cycloheptatrienol moieties of the coumarin derivative. Molecular orbitals computed for **5** ***ab + 2 dioxane*** and **11** ***ab + 2 dioxane*** suggest a significant contribution of the solvent to the electronic transitions.

For compound **5**, without the presence of two explicit solvent molecules around the ***ab*** moiety no emissive structure was found due to competition of the nonradiative channel through the twist around the C_2=C_3 double bond. However, the microsolvated ***ab + 2 dioxane*** and ***open ab + 2 dioxane*** system is predicted to emit at 2 eV. The optimization of the first singlet excited state results of ***ac + 2 dioxane*** molecules favor the planarity of the proton transfer site resulting

in barrierless ESIPT and population of the non-emissive structure with the labile proton bonded to the oxygen ***c***.

Thus, microsolvation using two dioxane molecules seems to be essential to describe the observed photochemistry of **5** and **11**, since the solvent participates in the electronic transitions and also favors coplanarity of the proton transfer sites, allowing the emission of ***ab + 2 dioxane*** and ***open ab + 2 dioxane***, predicted to be around 2 eV. The most important contrast between the computed emission energies of **5** and **11** relies on the parameter $\Delta E_{ab} - E_{open\ ab}$, which is close to zero for **5**, but around 0.4 eV for **11**. These findings are correlated with the emission spectra measured in dioxane, for which only one emission band is observed for compound **5**, while **11** shows two separate emission bands with ***open ab + 2 dioxane*** predicted to emit at a longer wavelength than ***ab + 2 dioxane***.

Table S2. Photophysically relevant conformers determined at ADC(2)/cc-pVDZ level of theory. Relative energies and dipole moments are given in electronvolts and Debye, respectively.

**3**	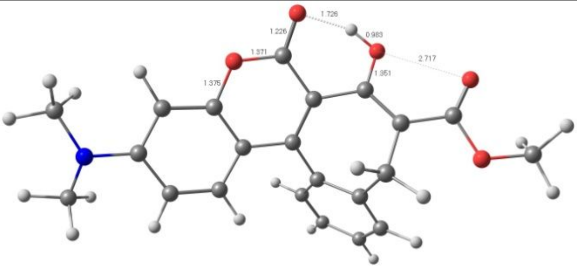	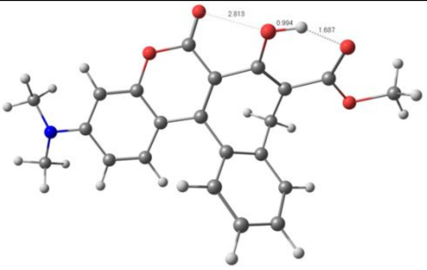
	ab	***ac***
**4**	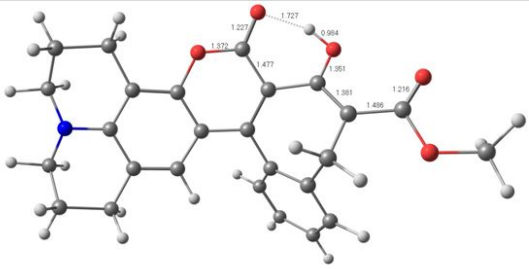	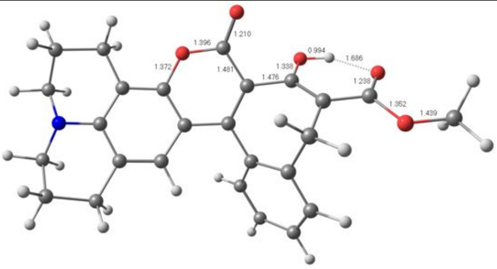
	ab	***ac***
**5**	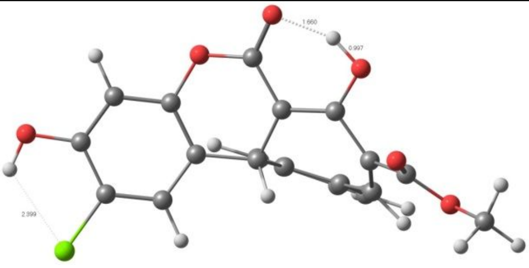	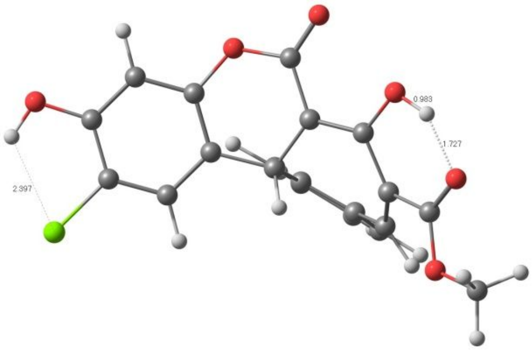
	ab	***ac***

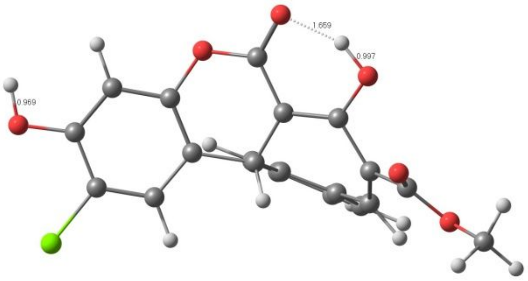

open ab

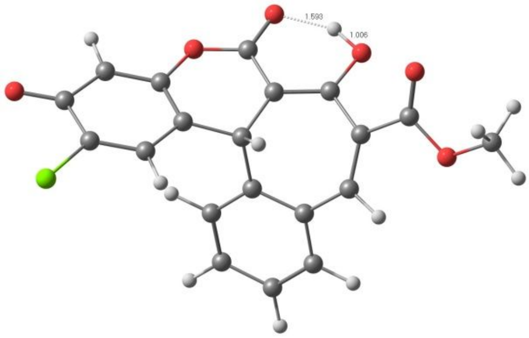

deprotonated ab

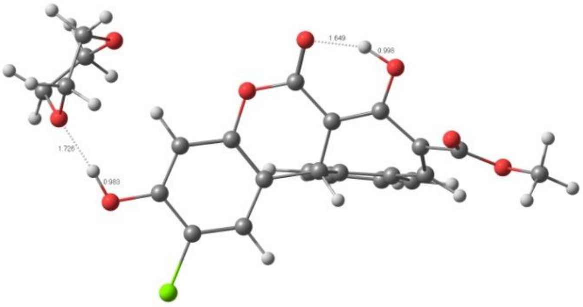

dioxane ab

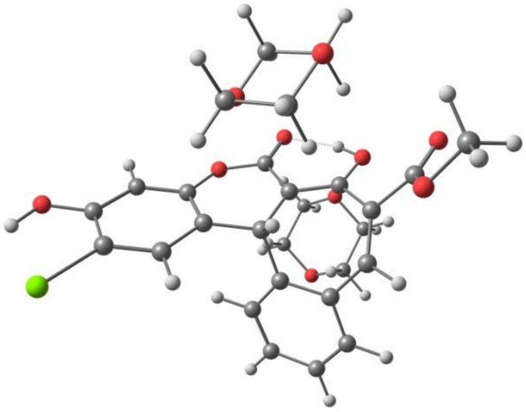

ab +2 dioxane

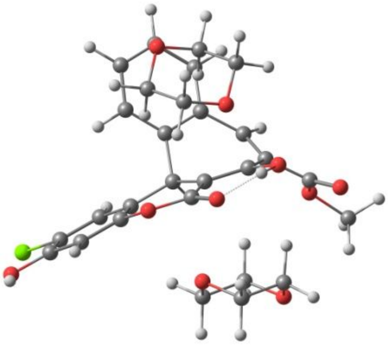

open ab + 2 dioxane

9

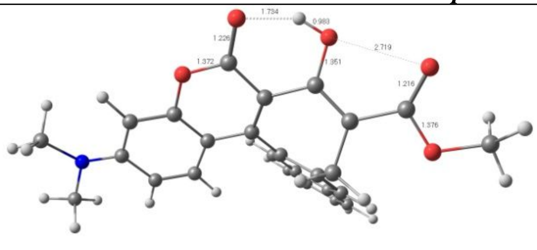

ab

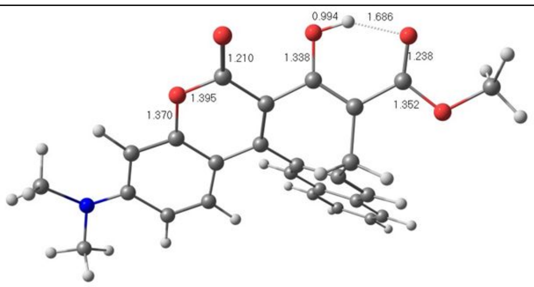

ac

11

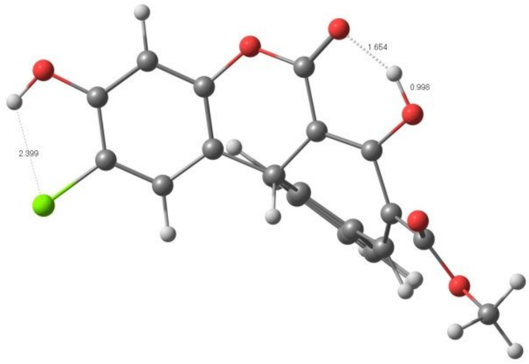

ab

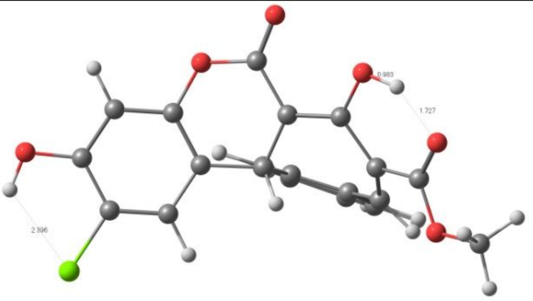

ac

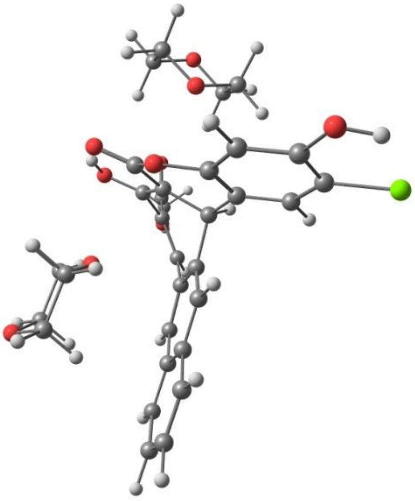

ab +2 dioxane

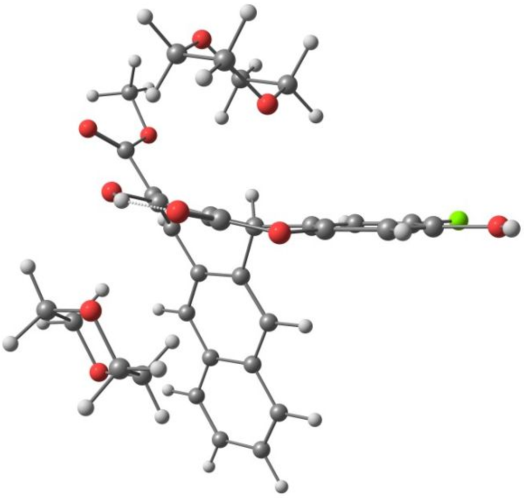

open ab + 2 dioxane

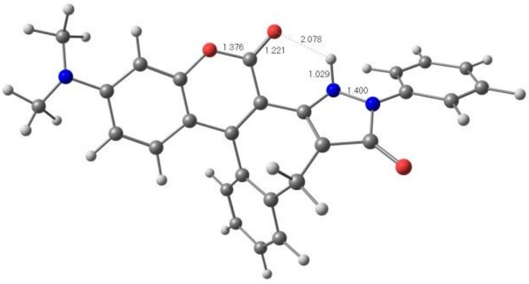

13

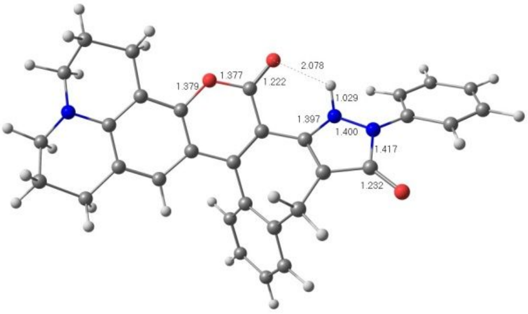

14

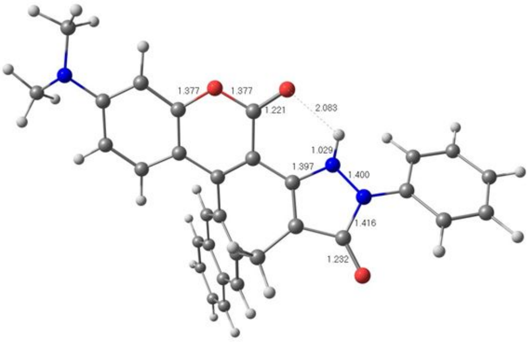

15

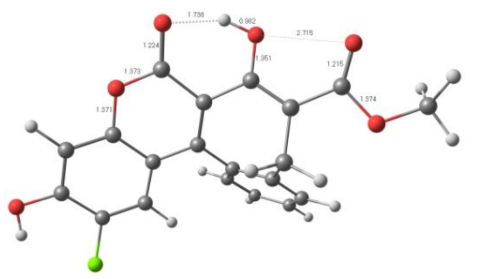

ab

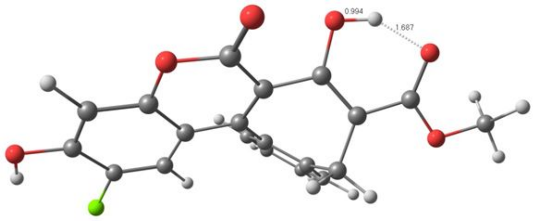

ac

16

Table S3. Total (E) and zero-point vibrational energies (ZPE) given in Hartree, dipole moments (μ), and leading electronic configurations of **3**, **4**, **5**, **9, 11, 13, 14, 15** and **16** computed for the MP2/cc-pVDZ equilibrium geometry of the ground state.

		E/Hartree	ZPE/Hartree	μ/Debye	el. conf.
3	***ab***	-1276.994	0.371	10.46	$(99a)^2$
	ac	-1277.004	0.372	8.20	$(99a)^2$
4	***ab***	-1431.382	0.445	8.68	$(113a)^2$
	ac	-1431.392	0.445	6.30	$(113a)^2$
5	***ab***	-1677.542	0.292	6.58	$(99)^2$
	ac	-1677.532	0.292	8.93	$(99)^2$
9	***ab***	-1430.170	0.417	10.64	$(112a)^2$
	ac	-1430.180	0.417	6.51	$(112a)^2$
11	***ab***	-1830.717	0.338	5.29	$(112a)^2$
	ac	-1830.708	0.338	7.56	$(112a)^2$
13		-1427.266	0.426	8.55	$(114a)^2$
14		-1581.655	0.500	10.59	$(128a)^2$
15		-1580.443	0.472	10.73	$(127a)^2$
16	***ab***	-1677.544	0.292	6.05	$(99a)^2$
	ac	-1677.555	0.293	5.83	$(99a)^2$

Table S4a. Vertical transition energy (ΔE), oscillator strength (f), dipole moment (μ), and leading electronic configurations of **3**, **4**, **5**, **9**, **11**, **13**, **14**, **15** and **16** computed with ADC(2)/cc-pVDZ method at the MP2/cc-pVDZ equilibrium geometry of the ground state.

		State	ΔE/eV	f	μ/Debye	el. conf.
3	***ab***	S_0	0.27	-	10.46	$(99a)^2$
		$^1\pi\pi^*$	3.08	0.44	8.14	0.93(99a-100a)
		$^1\pi\pi^*$	3.60	0.16	12.57	0.84(98a-100a)
		$^1n\pi^*$	4.06	0.00	6.65	0.79(93a-100a)
	ac	S_0	0.00	-	6.41	$(99a)^2$
		$^1\pi\pi^*$	3.38	0.62	13.48	0.95(99a-100a)
		$^1\pi\pi^*$	3.88	0.01	4.54	0.76(98a-100a)
		$^1\pi\pi^*$	4.13	0.00	11.36	0.75(96a-100a)
4	***ab***	S_0	0.26	-	8.68	$(113a)^2$
		$^1\pi\pi^*$	2.95	0.50	13.98	0.95(113a-114a)
		$^1\pi\pi^*$	3.46	0.04	8.26	0.88(112a-114a)
		$^1\pi\pi^*$	3.87	0.00	15.94	0.73(111a-114a)

	ac	S_0	0.00	-	8.08	$(113a)^2$
		$^1\pi\pi^*$	3.16	0.55	16.92	0.95(113a-114a)
		$^1\pi\pi^*$	3.81	0.00	6.31	0.82(112a-114a)
		$^1\pi\pi^*$	3.87	0.01	6.08	0.73(111a-114a)
5	***ab***	S_0	0.00	-	6.58	$(99)^2$
		$^1\pi\pi^*$	3.96	0.09	3.02	0.64(98a-100a)
		$^1\pi\pi^*$	4.40	0.08	9.01	0.75(97a-100a)
		$^1\pi\pi^*$	4.54	0.07	12.39	0.74(99a-100a)
	ac	S_0	0.24	-	8.93	$(99)^2$
		$^1\pi\pi^*$	3.44	0.07	2.61	0.88(98a-100a)
		$^1\pi\pi^*$	3.95	0.04	4.60	0.65(94a-100a)
		$^1\pi\pi^*$	4.06	0.02	12.38	0.78(99a-100a)
	open ab	S_0	0.12	-	2.97	$(99)^2$
		$^1\pi\pi^*$	3.96	0.09	2.83	0.59(98a-100a)
		$^1\pi\pi^*$	4.40	0.07	7.72	0.77(97a-100a)
		$^1\pi\pi^*$	4.48	0.08	13.16	0.77(99a-100a)
	deprot ab	S_0	-	-	19.40	$(99)^2$
		$^1\pi\pi^*$	1.71	0.00	6.01	0.98(99a-100a)
		$^1\pi\pi^*$	2.72	0.00	11.49	0.89(96a-100a)
		$^1\pi\pi^*$	3.04	0.02	9.20	0.98(99a-101a)
	dioxane ab	S_0	-	-	3.31	$(123)^2$
		$^1\pi\pi^*$	3.91	0.08	8.36	0.62(122a-124a)
		$^1\pi\pi^*$	4.26	0.04	17.53	0.81(123a-124a)
		$^1\pi\pi^*$	4.40	0.10	7.56	0.73(121a-124a)
	ab + 2 dioxane	S_0	0.00	-	5.56	$(147)^2$
		$^1\pi\pi^*$	3.75	0.07	3.95	0.61(147a-148a)
		$^1\pi\pi^*$	4.24	0.05	5.16	0.59(145a-148a)
		$^1\pi\pi^*$	4.37	0.02	9.28	0.50(146a-148a)
	open ab + 2 dioxane	S_0	0.09	-	3.06	$(147)^2$
		$^1\pi\pi^*$	3.73	0.07	4.24	0.57(146a-148a)
		$^1\pi\pi^*$	4.20	0.06	5.13	0.61(145a-148a)
		$^1\pi\pi^*$	4.32	0.02	13.99	0.68(147a-148a)
9	***ab***	S_0	0.27	-	10.64	$(112a)^2$
		$^1\pi\pi^*$	3.00	0.36	8.14	0.84(112a-113a)
		$^1\pi\pi^*$	3.51	0.20	15.22	0.69(111a-113a)
		$^1\pi\pi^*$	3.82	0.02	11.99	0.63(109a-113a)
	ac	S_0	0.00	-	8.37	$(112a)^2$
		$^1\pi\pi^*$	3.31	0.55	12.53	0.82(112a-113a)
		$^1\pi\pi^*$	3.73	0.04	8.70	0.66(111a-113a)
		$^1\pi\pi^*$	4.05	0.00	6.33	0.44(108a-113a)
11	***ab***	S_0	0.00	-	6.96	$(112a)^2$
		$^1\pi\pi^*$	3.66	0.12	9.38	0.83(112a-113a)
		$^1\pi\pi^*$	4.07	0.04	5.58	0.55(111a-113a)
		$^1\pi\pi^*$	4.26	0.32	9.70	0.57(111a-113a)
	ac	S_0	0.28	-	9.24	$(112a)^2$
		$^1\pi\pi^*$	3.25	0.10	5.15	0.78(112a-113a)
		$^1\pi\pi^*$	3.74	0.01	8.10	0.53(109a-113a)
		$^1\pi\pi^*$	3.89	0.12	6.00	0.51(110a-113a)
	ab + 2 dioxane	S_0	-	-	5.91	$(160a)^2$
		$^1\pi\pi^*$	3.56	0.08	4.80	0.75(160a-161a)

		$^1\pi\pi^*$	3.91	0.00	5.52	0.62(157a-161a)
		$^1\pi\pi^*$	4.20	0.31	9.63	0.73(159a-161a)
	open ab* + 2 *dioxane	S_0	-	-	3.32	$(160a)^2$
		$^1\pi\pi^*$	3.59	0.09	5.41	0.77(160a-161a)
		$^1\pi\pi^*$	3.95	0.02	5.73	0.56(157a-161a)
		$^1\pi\pi^*$	4.16	0.22	7.11	0.66(158a-161a)
13	***keto***	S_0	-	-	10.50	$(114a)^2$
		$^1\pi\pi^*$	2.93	0.04	8.51	0.61(113a-115a)
		$^1\pi\pi^*$	3.17	0.67	15.02	0.69(114a-115a)
		$^1n\pi^*$	3.84	0.02	5.24	0.80(106a-115a)
14	**ADC(2)**	S_0	-	-	10.59	$(128a)^2$
		$^1\pi\pi^*$	2.94	0.11	6.99	0.56(127a-129a)
		$^1\pi\pi^*$	2.97	0.53	17.88	0.71(128a-129a)
		$^1n\pi^*$	3.79	0.02	2.97	0.57(120a-129a)
	**CC2**	S_0	-	-	8.91	$(128a)^2$
		$^1\pi\pi^*$	3.14	0.81	16.86	0.87(128a-129a)
		$^1\pi\pi^*$	3.19	0.03	7.29	0.76(127a-129a)
		$^1n\pi^*$	3.94	0.01	14.08	0.81(124a-129a)
	**SOS-CC2**	S_0	-	-	8.92	$(128a)^2$
		$^1\pi\pi^*$	3.45	0.81	16.33	0.82(128a-129a)
		$^1\pi\pi^*$	3.84	0.09	6.13	0.68(127a-129a)
		$^1n\pi^*$	4.11	0.01	11.61	0.67(127a-129a)
15	***keto***	S_0	-	-	10.73	$(127a)^2$
		$^1\pi\pi^*$	2.89	0.04	8.61	0.71(126a-128a)
		$^1\pi\pi^*$	3.11	0.63	13.87	0.79(127a-128a)
		$^1\pi\pi^*$	3.68	0.06	13.56	0.74(125a-128a)
16	***ab***	S_0	0.28	-	7.67	$(99a)^2$
		$^1\pi\pi^*$	3.10	0.20	7.58	0.89(99a-100a)
		$^1\pi\pi^*$	3.89	0.18	7.61	0.68(98a-100a)
		$^1n\pi^*$	3.96	0.04	8.22	0.76(94a-100a)
	ac	S_0	0.00	-	7.15	$(99a)^2$
		$^1\pi\pi^*$	3.59	0.33	8.12	0.86(99a-100a)
		$^1\pi\pi^*$	3.88	0.09	4.91	0.60(98a-100a)
		$^1\pi\pi^*$	4.26	0.03	3.82	0.53(98a-100a)

Table S4b. Relevant molecular π orbitals involved into the lowest electronic excitations for the most stable isomer of each derivative.

**HOMO**		**LUMO**	
**3 *ac***			
98a	*99a*	*100a*	*101a*
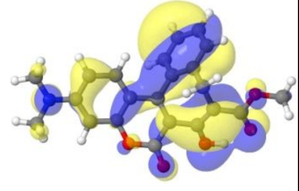	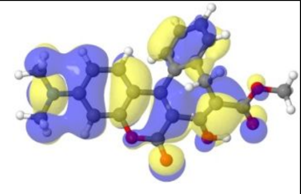	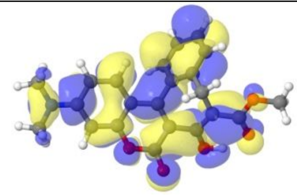	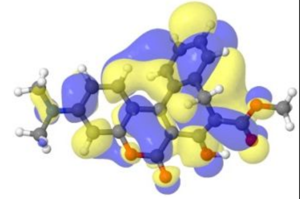

**4** ***ac***			
112a	*113a*	*114a*	*115a*
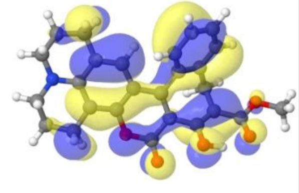	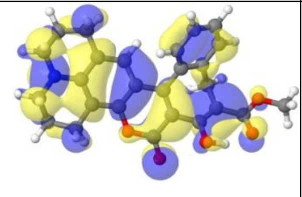	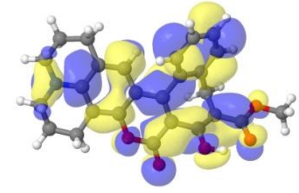	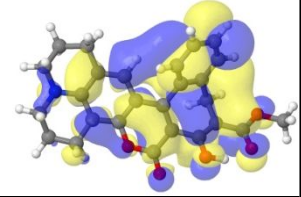
**5** ***ac***			
98	*99*	*100*	*101*
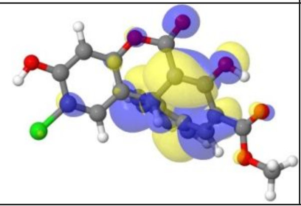	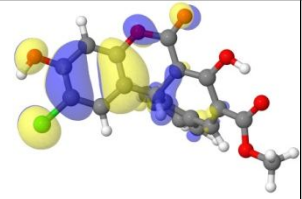	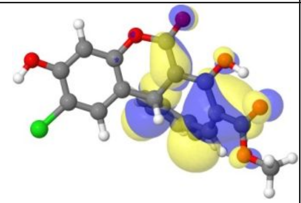	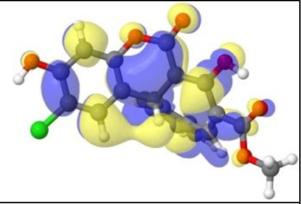
**5** ***ab***			
98	*99*	*100*	*101*
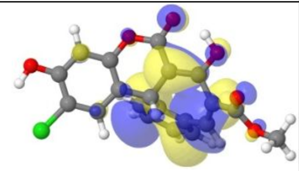	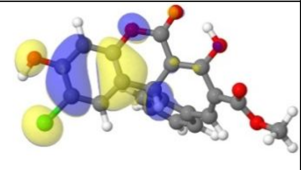	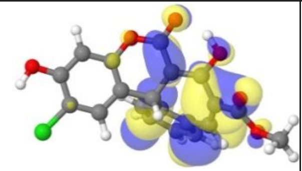	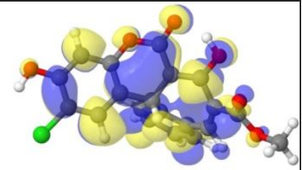
**5** ***ab + 2dioxane***			
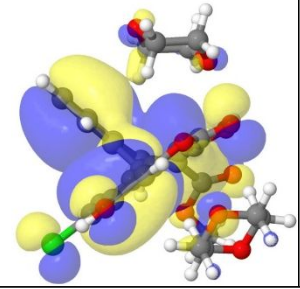	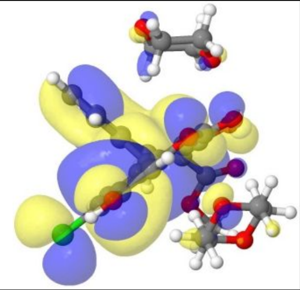	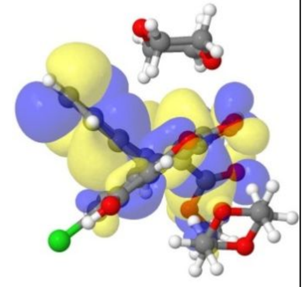	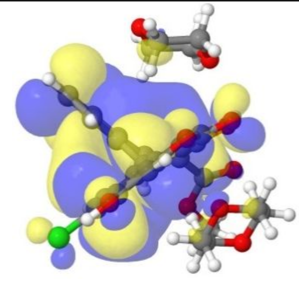
**9** ***ac***			
111a	*112a*	*113a*	*114a*
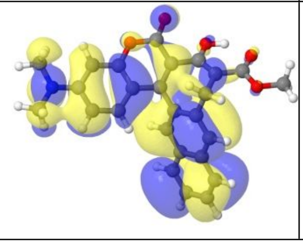	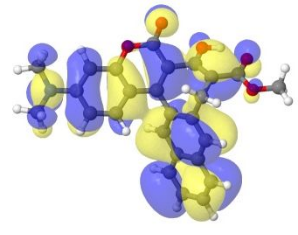	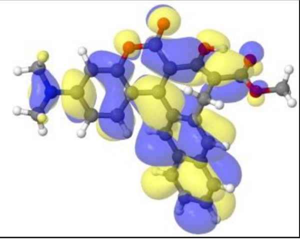	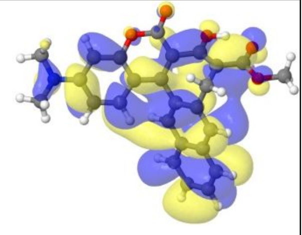
11 ab			
111	*112*	*113*	*114*
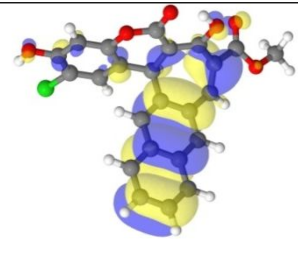	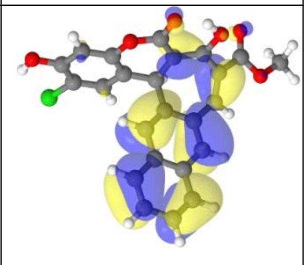	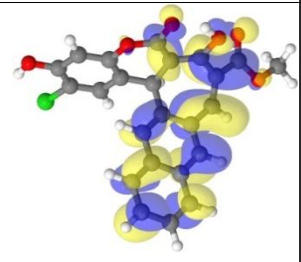	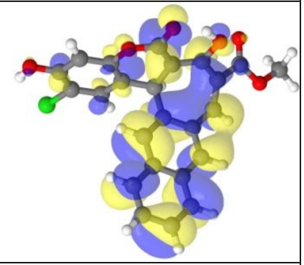
11 ab + 2dioxane			
159	*160*	*161*	*162*

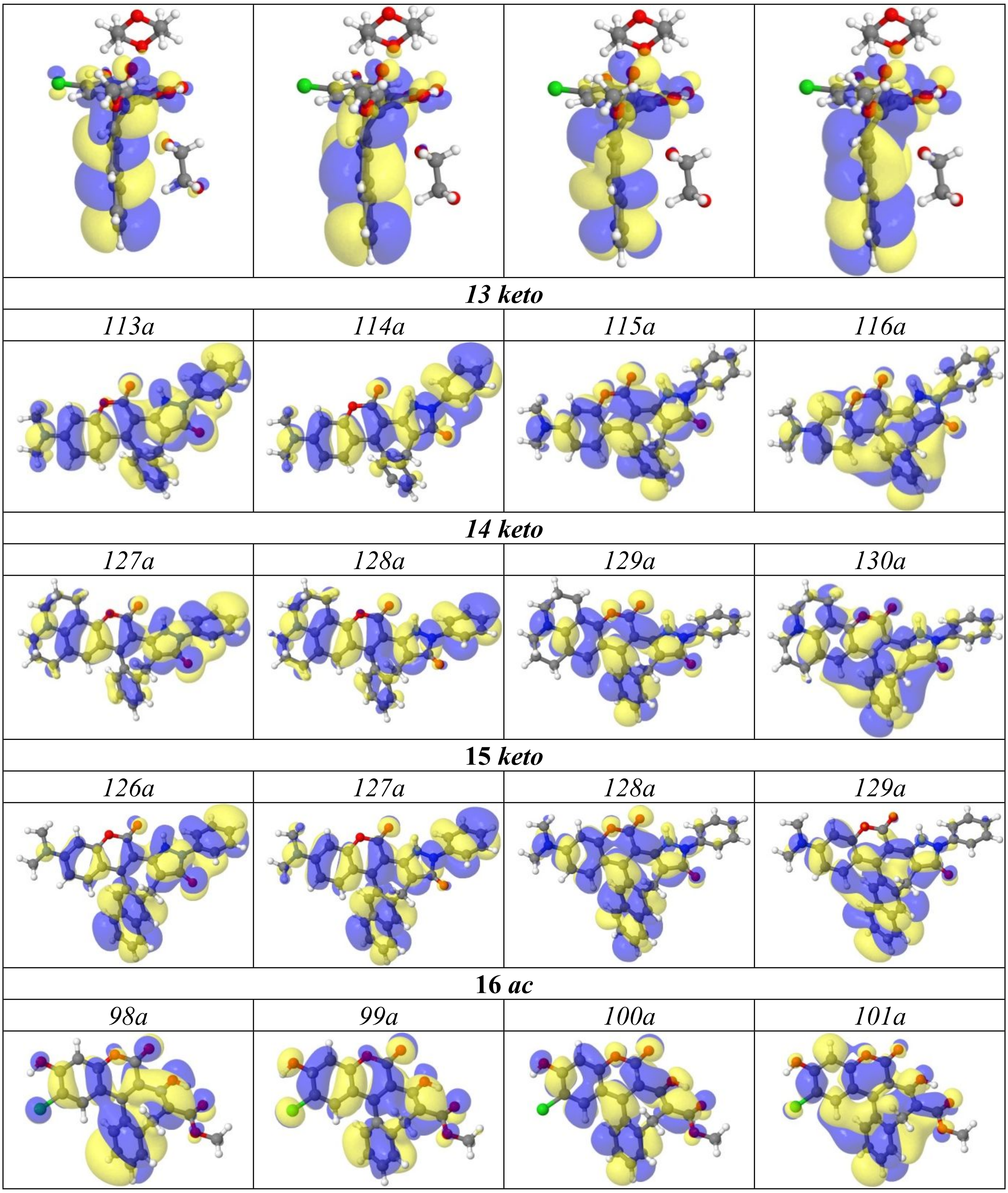

Table S5. Adiabatic energy of the fluorescing state (E(S_1)) for the most photophysically relevant species, vertical transition energy (ΔE), oscillator strength (f) and dipole moments (μ(S_1) and μ(S_0)), computed with ADC(2)/cc-pVDZ method at the equilibrium geometry of the S_1 (S_2^{**}) state.

		E(S_1)/eV	ΔE/eV	*f*	μ(S_1)/D	μ(S_0)/D
3	***ab***	1.97	-0.08	-	4.68	15.96
	ac	2.79	1.61, 2.31*	0.25, 0.56*	7.79, 7.33*	11.31, 8.03*
4	***ab***	1.96	-0.06	-	4.63	16.25
	ac	2.73	1.79, 2.39*	0.38, 0.70*	7.97, 9.94*	11.26, 8.25*

5	***ab***	4.72	-0.33	-	2.99	7.99
	ac	4.22	-0.66	-	4.04	11.00
	open ab	4.84	-0.30	-	1.63	6.94
	ab deprotonated	-	0.35, 1.19	-	5.63	22.65
	ab + 2 dioxane	-	1.21, 2.01[*]	0.03, 0.09[*]	3.60, 2.51[*]	8.00, 5.10[*]
	ac + 2 dioxane	-	-0.13	-	4.26	12.57
	ab deprotonated + 2 dioxane	-	-0.02	-	4.45	22.60
	open ab +2 dioxane	-	1.18, 1.99[*]	0.03, 0.09[*]	4.32, 2.14[*]	6.15, 3.26[*]
9	***ab***	1.87	-0.24	-	4.75	16.21
	ac	2.68	1.46, 2.19[*]	0.45[*]	6.62, 6.65[*]	11.67, 7.92[*]
11	***ab***	-	0.54, 1.37[*]	0.02, 0.09[*]	3.26, 4.12[*]	10.91, 6.73[*]
	ac	-	-0.65	-	4.18	12.36
	ab + 2 dioxane	-	1.09, 1.91[*]	0.04, 0.12[*]	3.42, 2.05[*]	9.09, 5.57[*]
	ac + 2 dioxane	-	-0.22	-	4.71	12.75
	open ab + 2 dioxane	-	1.50, 2.27[*]	0.06, 0.13[*]	5.28, 3.33[*]	6.18, 2.99[*]
13	***enol***	0.97	-0.69	-	1.39	18.47
14	***enol***	0.97	-0.65	-	1.36	18.91
	keto[**]	2.57	1.95, 2.92[*]	0.01[*]	3.82, 5.20[*]	13.54, 10.54[*]
	keto + 2 acetonitrile[**]	-	1.73, 2.42[*]	0.78[*]	10.25, 12.97[*]	19.72, 15.12[*]
15	***enol***	0.91	-0.73	-	1.31	18.96
16	***ab***	1.90	-0.21	-	4.09	12.17
	ac	2.78	1.51, 2.28[*]	0.35[*]	7.06, 6.69[*]	8.97, 6.11[*]

[*] Data obtained at SOS-CC2/cc-pVDZ level of theory.

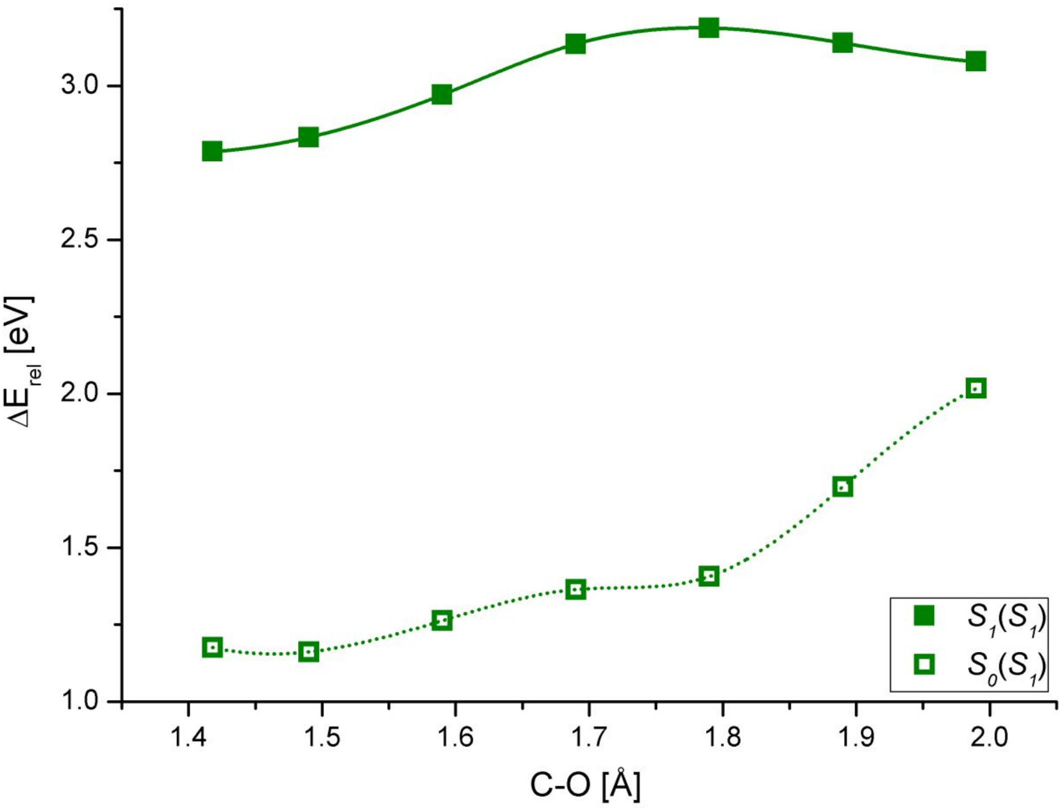

3

Figure S6. Minimum energy profiles for the open-ring reaction for the respective ***ac*** isomer of **3** in the ground (S_0) and first excited singlet state (S_1) computed at MP2/ADC(2)/cc-pVDZ level of theory, respectively. Symbols connected by solid line denote energy profile optimized in the S_1 state, while dashed lines denotes vertical energy of the S_0.

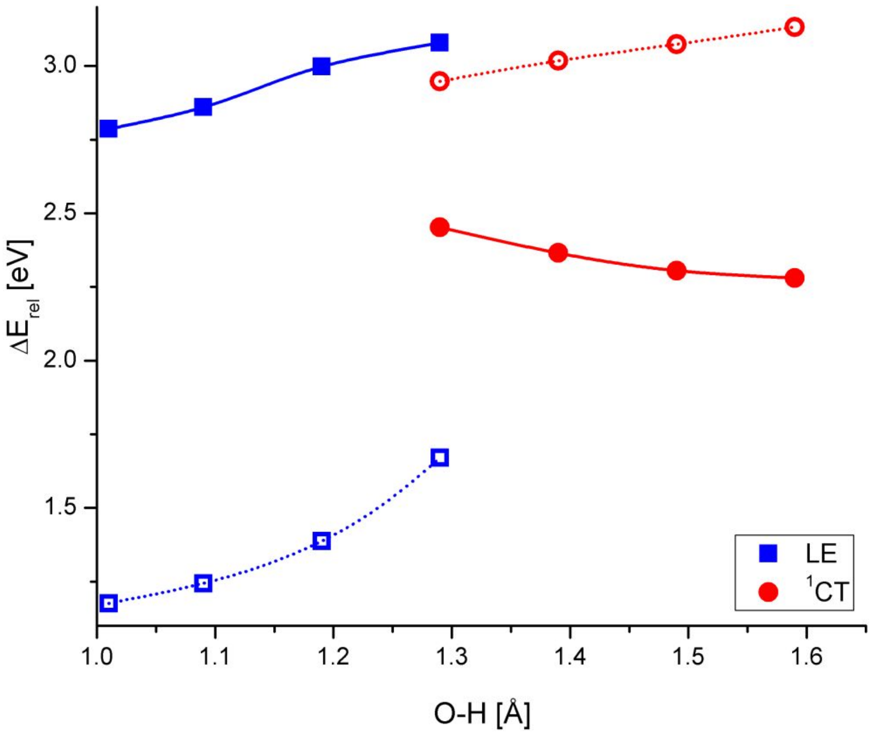

3

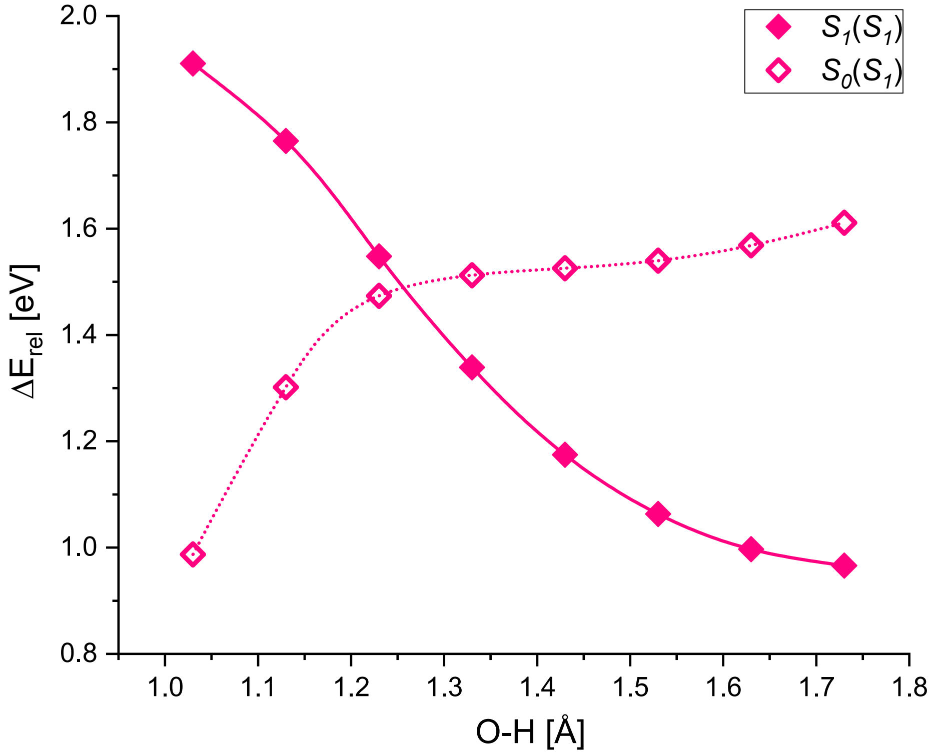

13

Figure S7. Potential energy (PE) profiles for hydrogen transfer for the respective ***ac*** isomer of **3** and **13** in the first excited singlet state (S_1) (symbols connected by solid lines) along the minimum-energy path (MEP) for locally excited (squares) and charge transfer (circles) states electron transfer ground (S_0) and computed at MP2/ADC(2)/cc-pVDZ level of theory, respectively. Symbols connected by solid line denote energy profile optimized in the S_1 state, while dashed lines denotes vertical energy of the S_0.

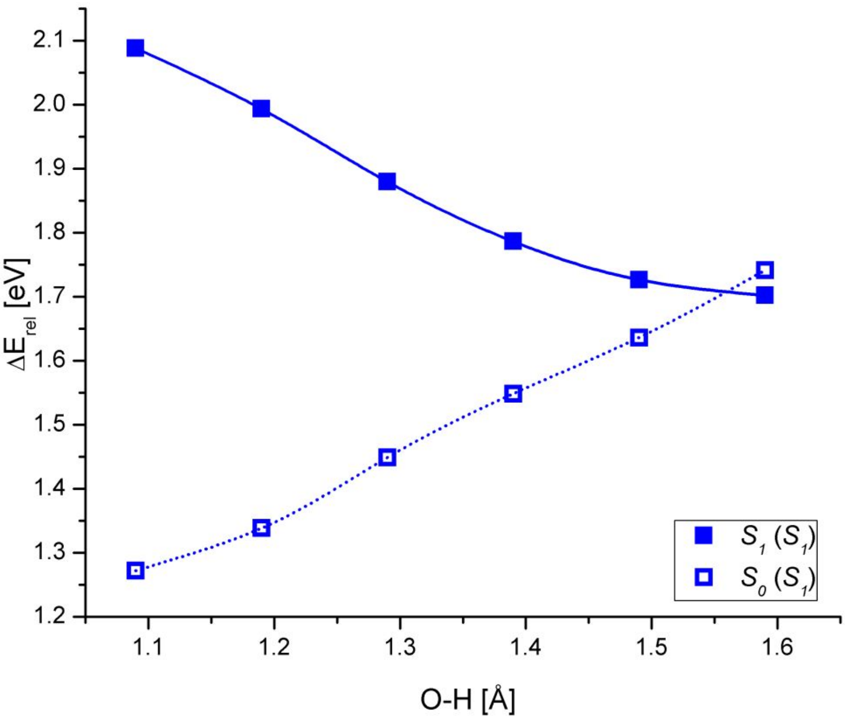

3

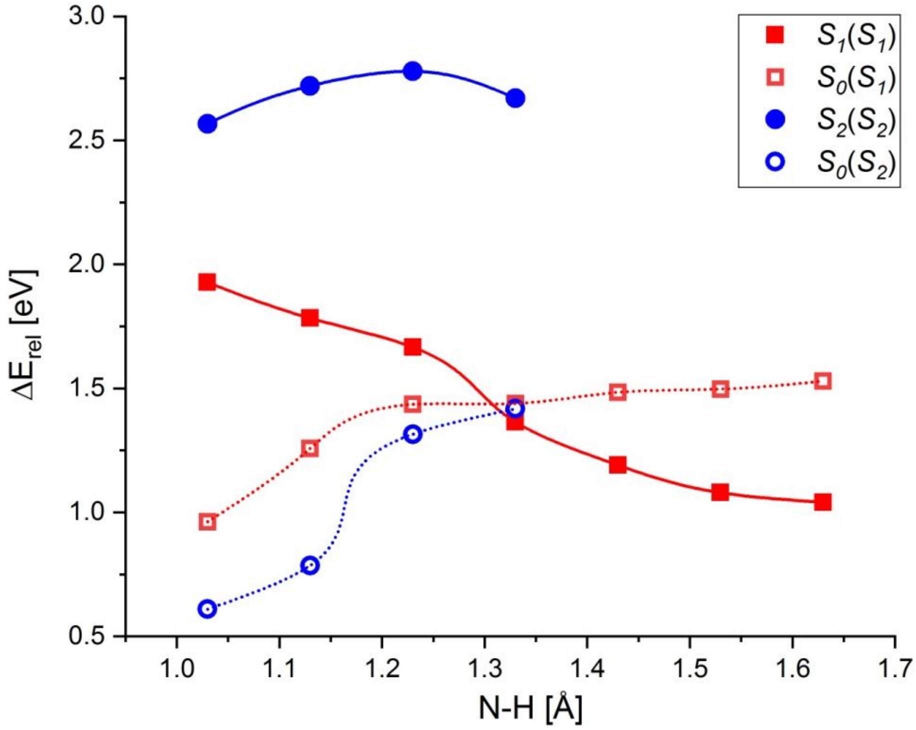

14

Figure S8. Minimum-energy profiles for hydrogen transfer for the respective ***ab*** isomer of **3** and **14** in the ground (S_0) and first excited singlet states computed at, respectively. Symbols connected by solid line denote energy profile optimized in the S_1 and S_2) states, while dashed lines denotes vertical energy of the S_0.

Table S6. Nuclear conformations and S_1-S_0 electron density difference computed at ADC(2)/cc-pVDZ level of theory. Red (blue) indicates electron acceptor (donor) regions of **3** computed at MP2/ADC(2)/cc-pVDZ level of theory at the discontinuity of the PE landscape showed in the **Figure S6** (O-H bond distance=1.3 Å).

Molecular Structure	S_1-S_0 Density Difference
^{1}LE state<br>μ_{S1} = 6.89; μ_{S0} = 10.60<br>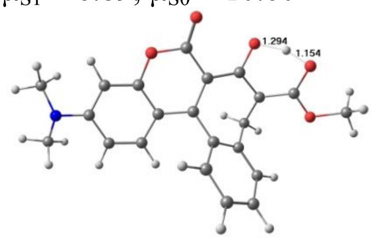	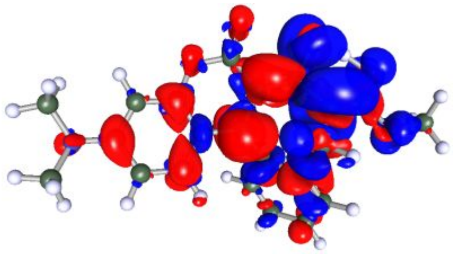
^{1}CT state<br>μ_{S1} = 2.32; μ_{S0} = 10.77<br>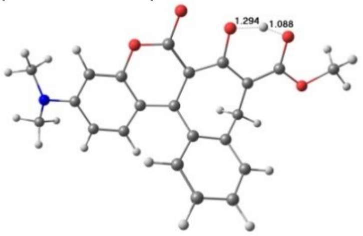	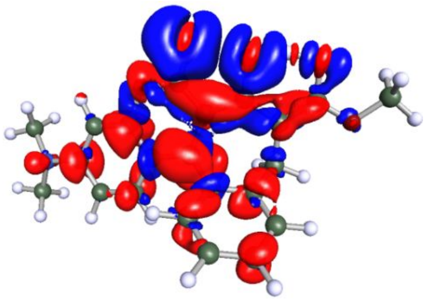

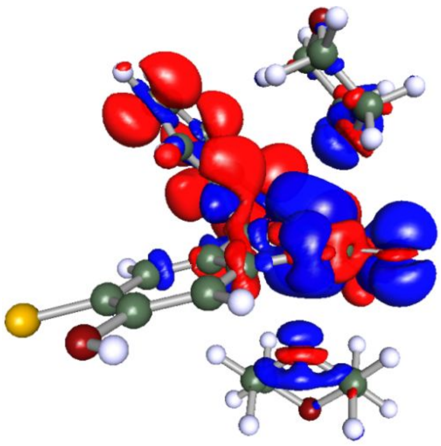

5

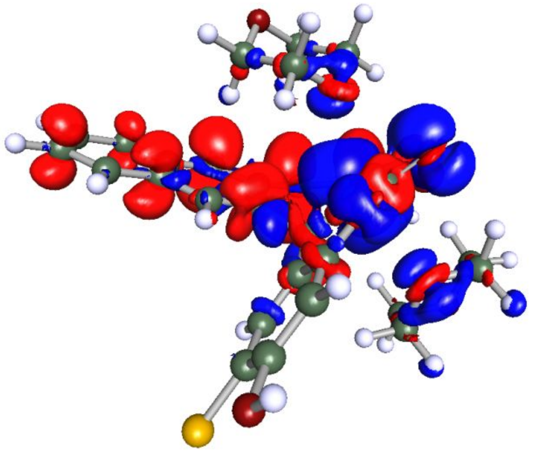

11

Figure S9. S_1-S_0 electron density difference computed at ADC(2)/cc-pVDZ level of theory. Red (blue) indicates electron acceptor (donor) regions of ***open ab*** isomer of **5** and **11** computed at MP2/ADC(2)/cc-pVDZ level of theory.

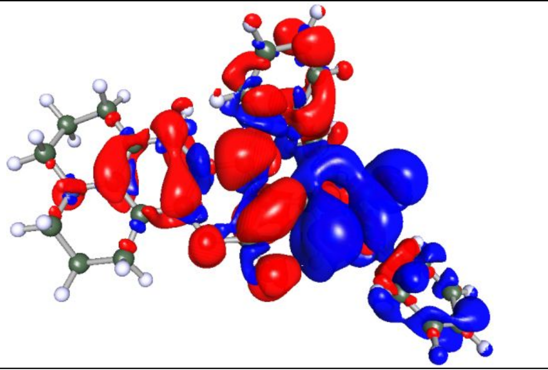	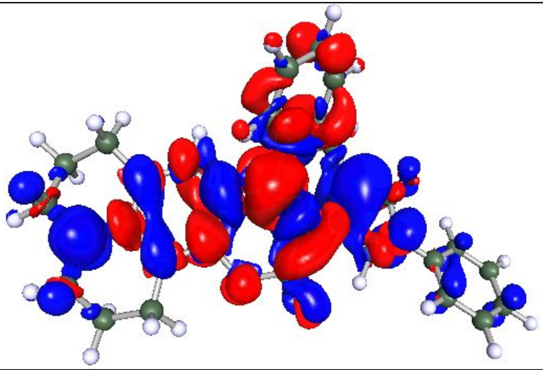
***S_1* (LE)**	***S_2* (CT)**

Figure S10. Electron density differences computed at MP2/ADC(2)/cc-pVDZ level of theory**.** Red (blue) indicates electron acceptor (donor) regions of **14**.

4. NMR Spectra.

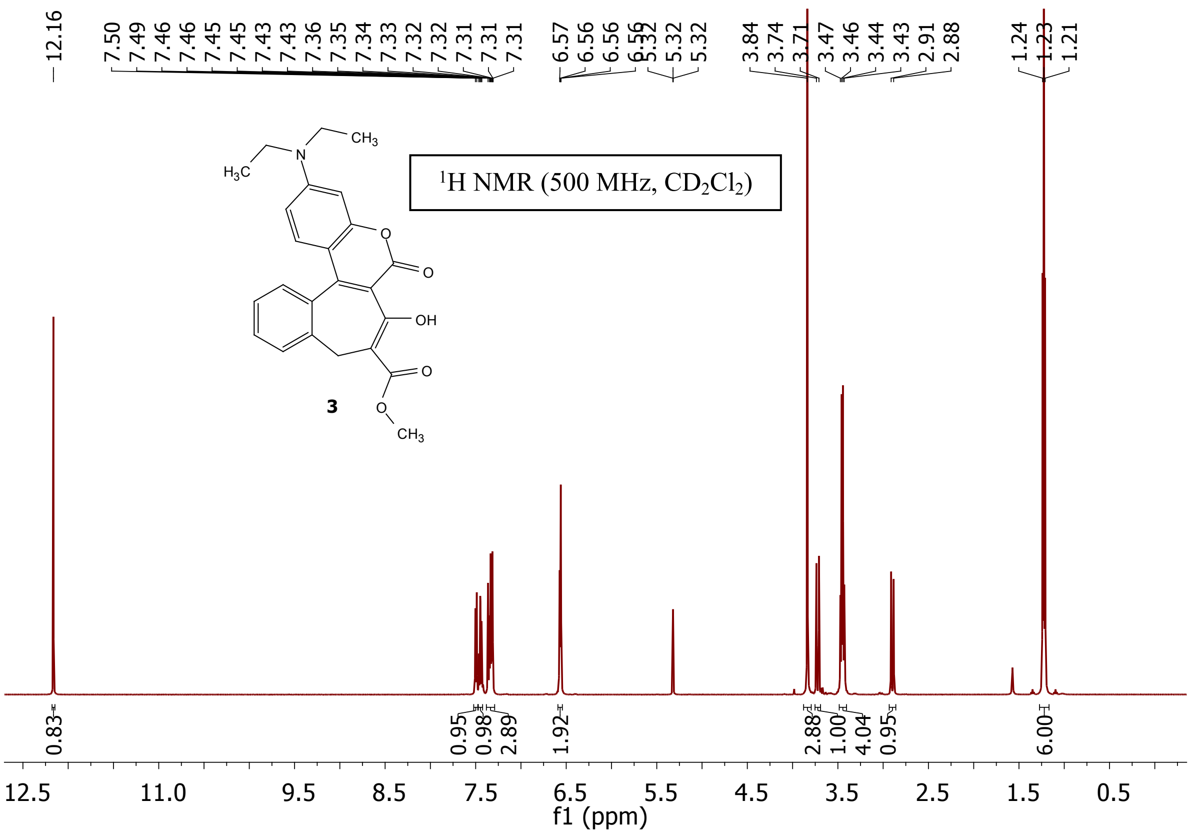

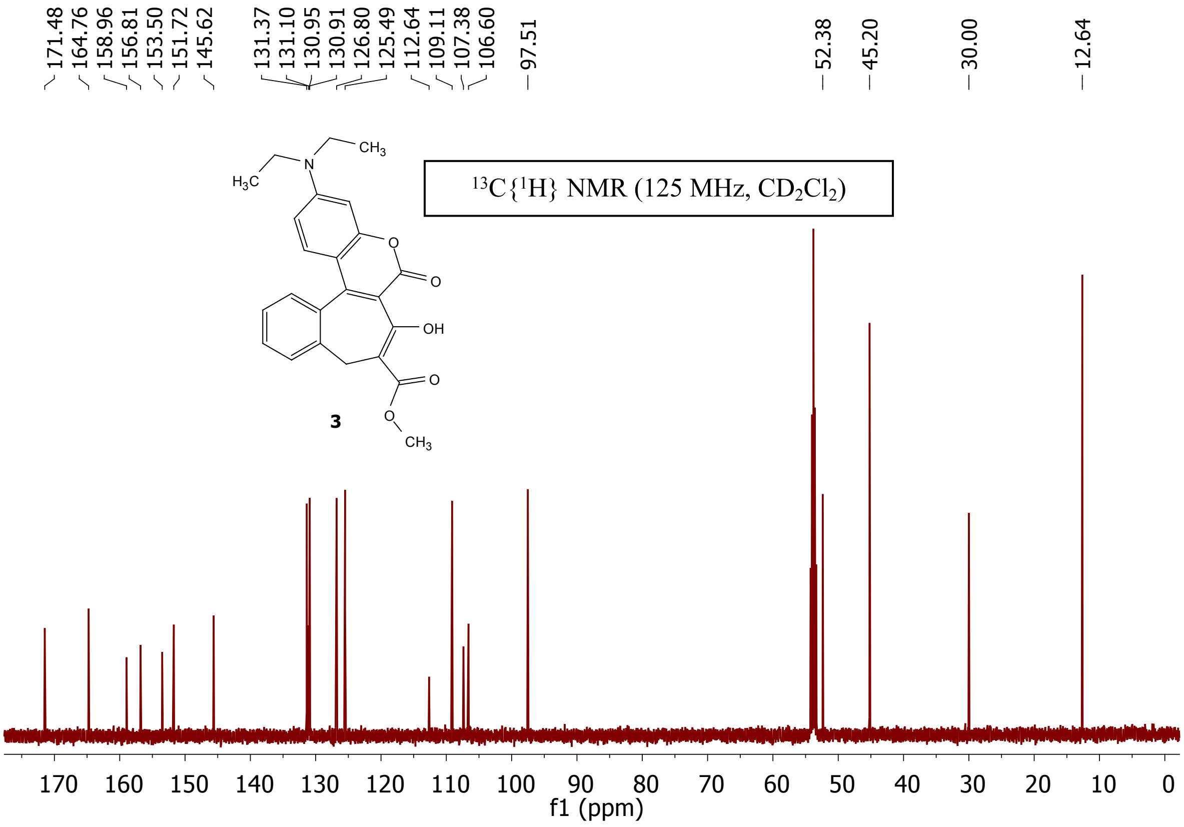

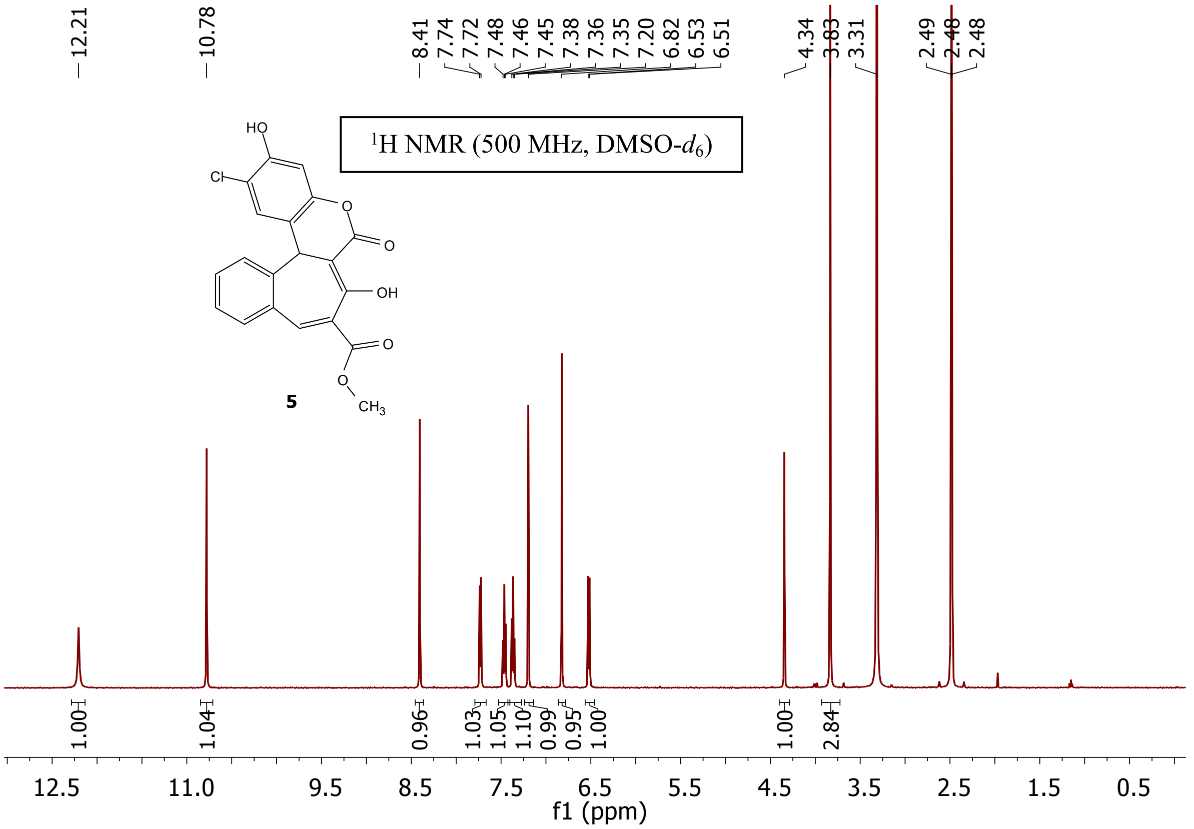
12.21
10.78
8.41
7.74
7.72
7.48
7.46
7.45
7.38
7.36
7.35
7.20
6.82
6.53
6.51
4.34
3.83
3.31
2.49
2.48
2.48
^{1}H NMR (500 MHz, DMSO-d_6)
HO
Cl
O
O
OH
O
O
CH3
5
1.00
1.04
0.96
1.03
1.05
1.10
0.99
0.95
1.00
1.00
2.84
12.5
11.0
9.5
8.5
7.5
6.5
5.5
4.5
3.5
2.5
1.5
0.5
f1 (ppm)

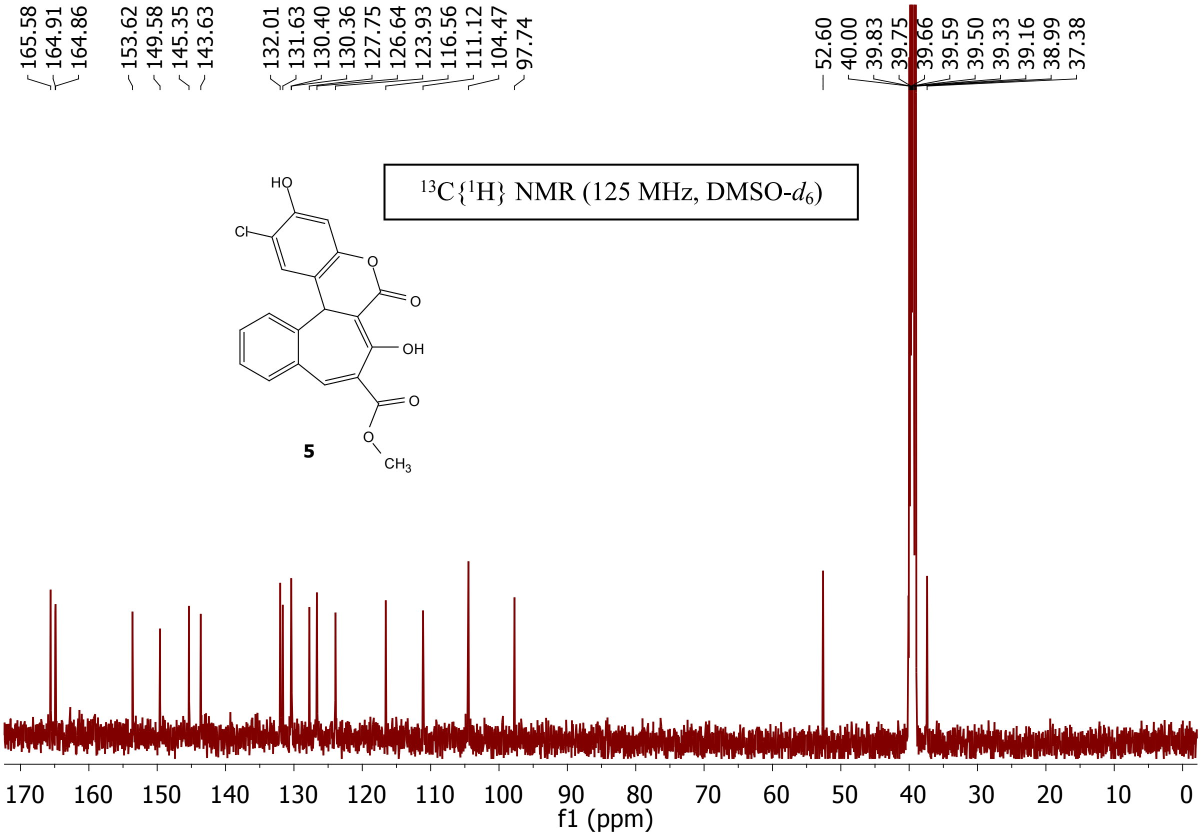
165.58
164.91
164.86
153.62
149.58
145.35
143.63
132.01
131.63
130.40
130.36
127.75
126.64
123.93
116.56
111.12
104.47
97.74
52.60
40.00
39.83
39.75
39.66
39.59
39.50
39.33
39.16
38.99
37.38
^{13}C{^{1}H} NMR (125 MHz, DMSO-d_6)
HO
Cl
O
O
OH
O
O
CH3
5
170
160
150
140
130
120
110
100
90
80
70
60
50
40
30
20
10
0
f1 (ppm)

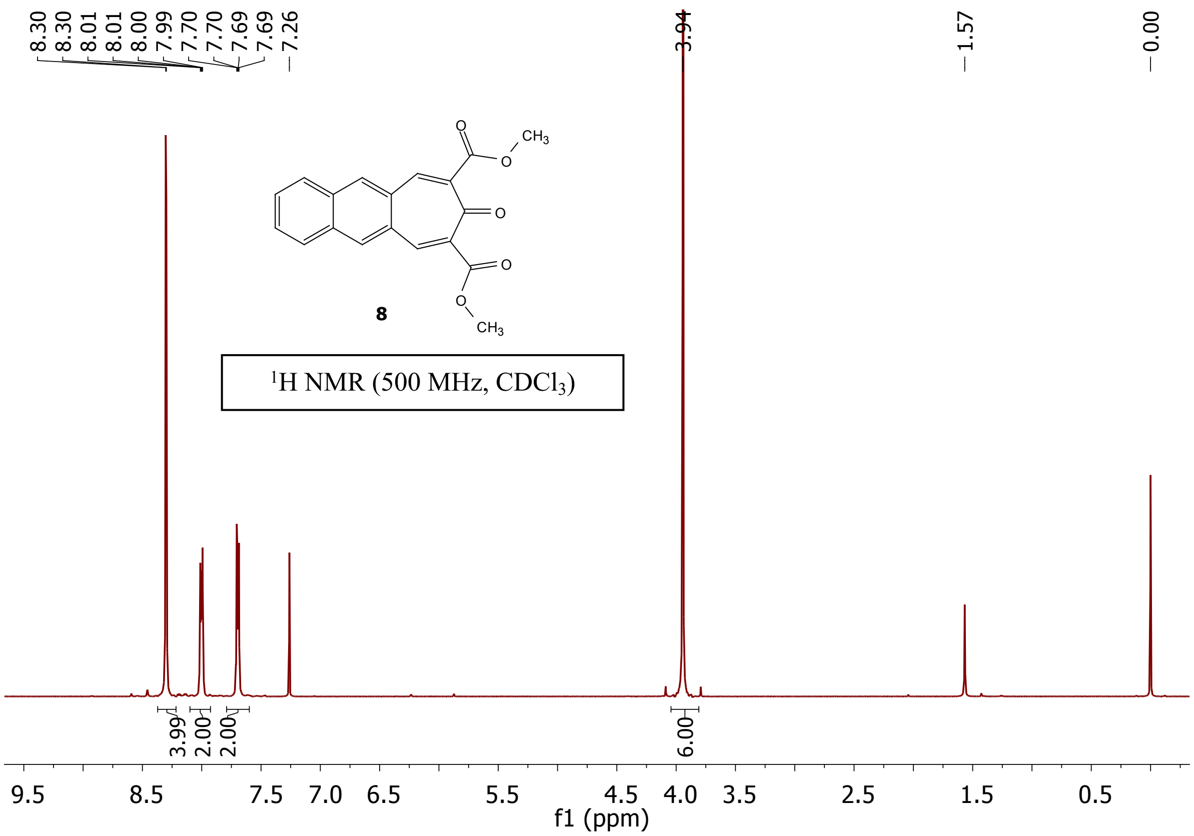
8.30
8.30
8.01
8.01
8.00
7.99
7.70
7.70
7.69
7.69
7.26
3.94
1.57
0.00
8
¹H NMR (500 MHz, CDCl₃)
3.99
2.00
2.00
6.00
9.5
8.5
7.5
7.0
6.5
5.5
4.5
4.0
3.5
2.5
1.5
0.5
f1 (ppm)

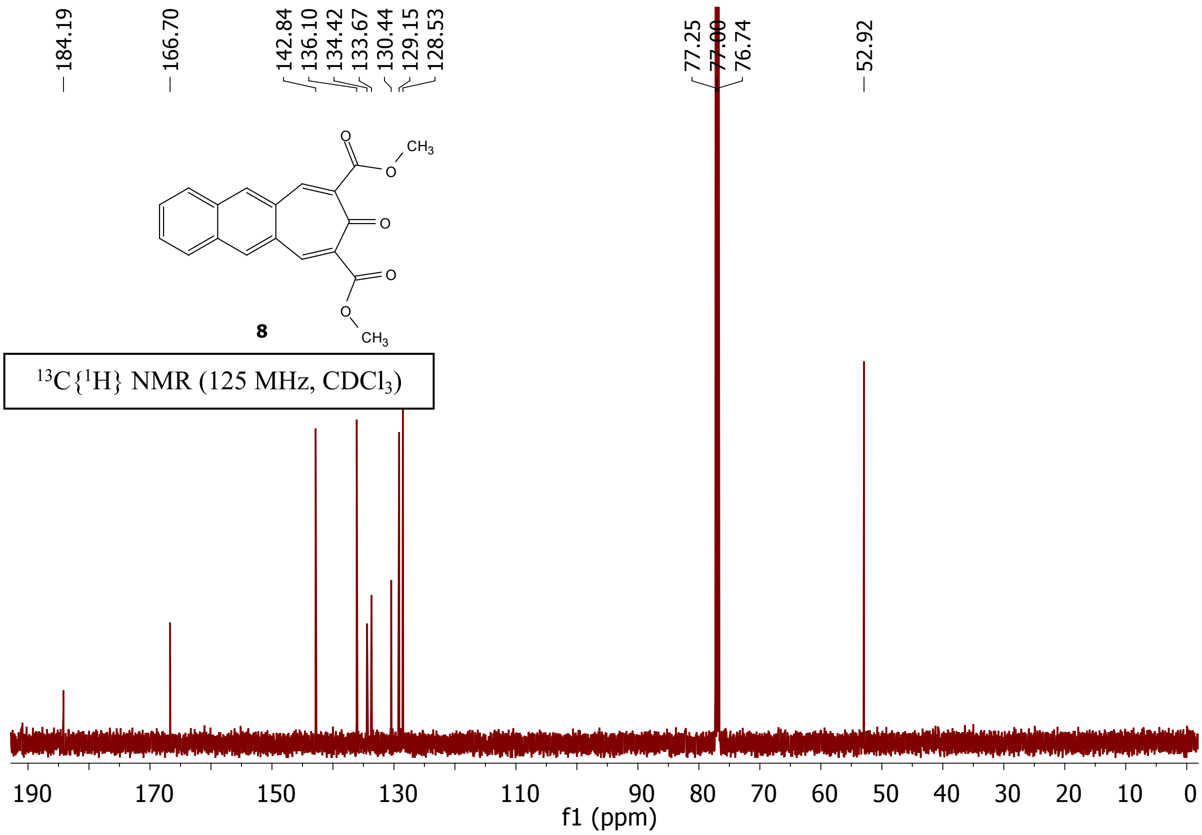
184.19
166.70
142.84
136.10
134.42
133.67
130.44
129.15
128.53
77.25
77.00
76.74
52.92
8
¹³C{¹H} NMR (125 MHz, CDCl₃)
190
170
150
130
110
90
80
70
60
50
40
30
20
10
0
f1 (ppm)

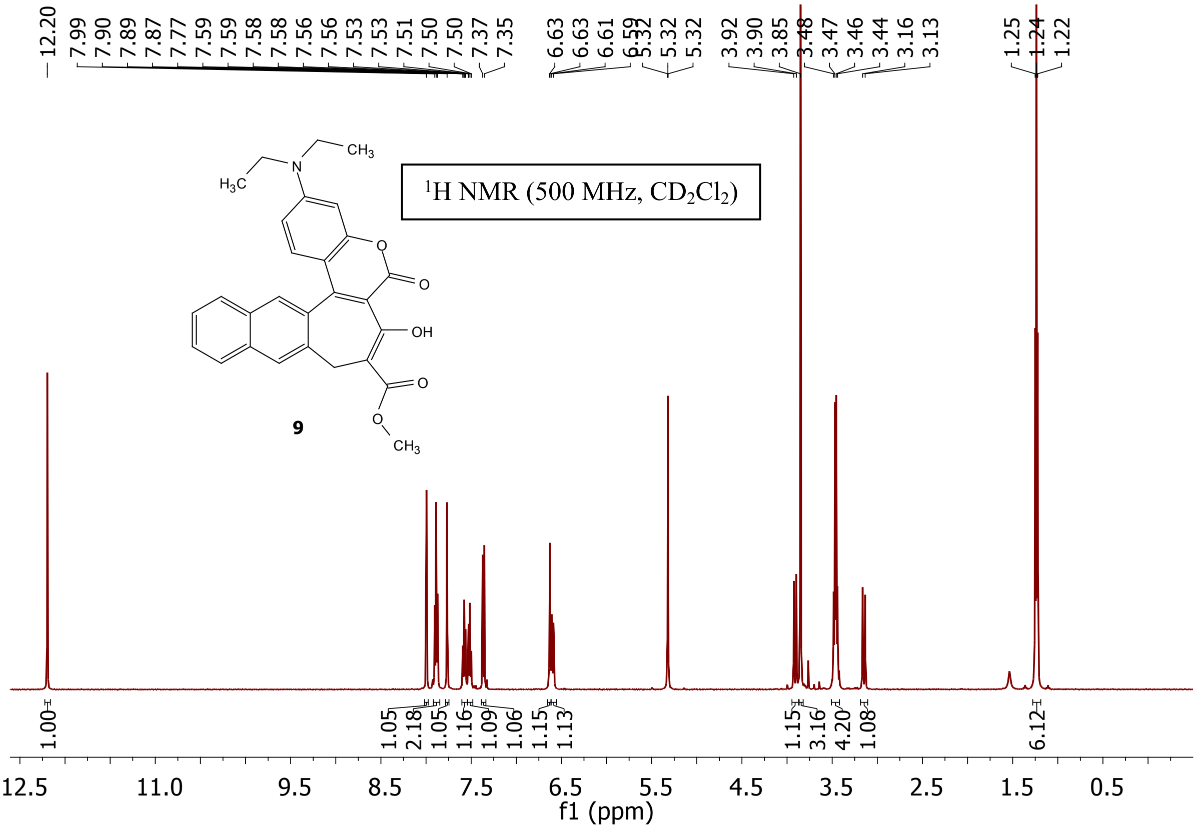
1H NMR (500 MHz, CD2Cl2)
9
f1 (ppm)

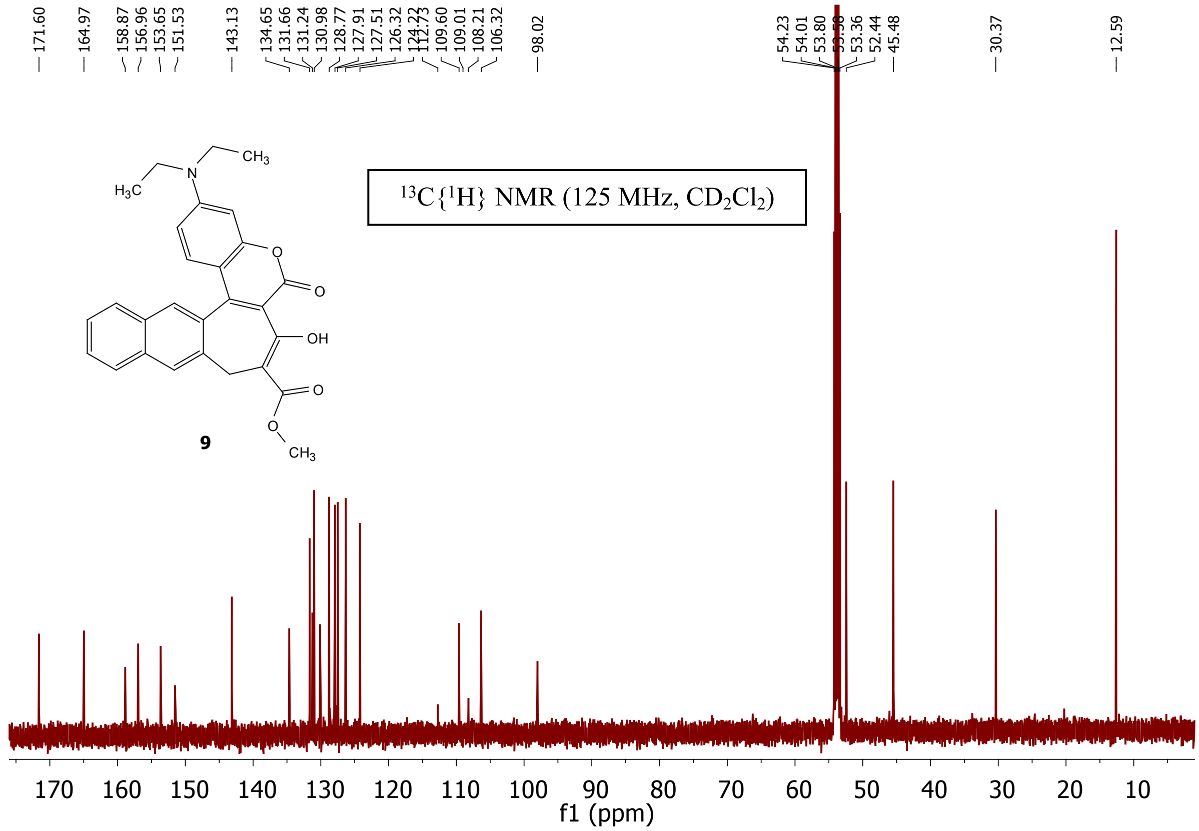
13C{1H} NMR (125 MHz, CD2Cl2)
9
f1 (ppm)

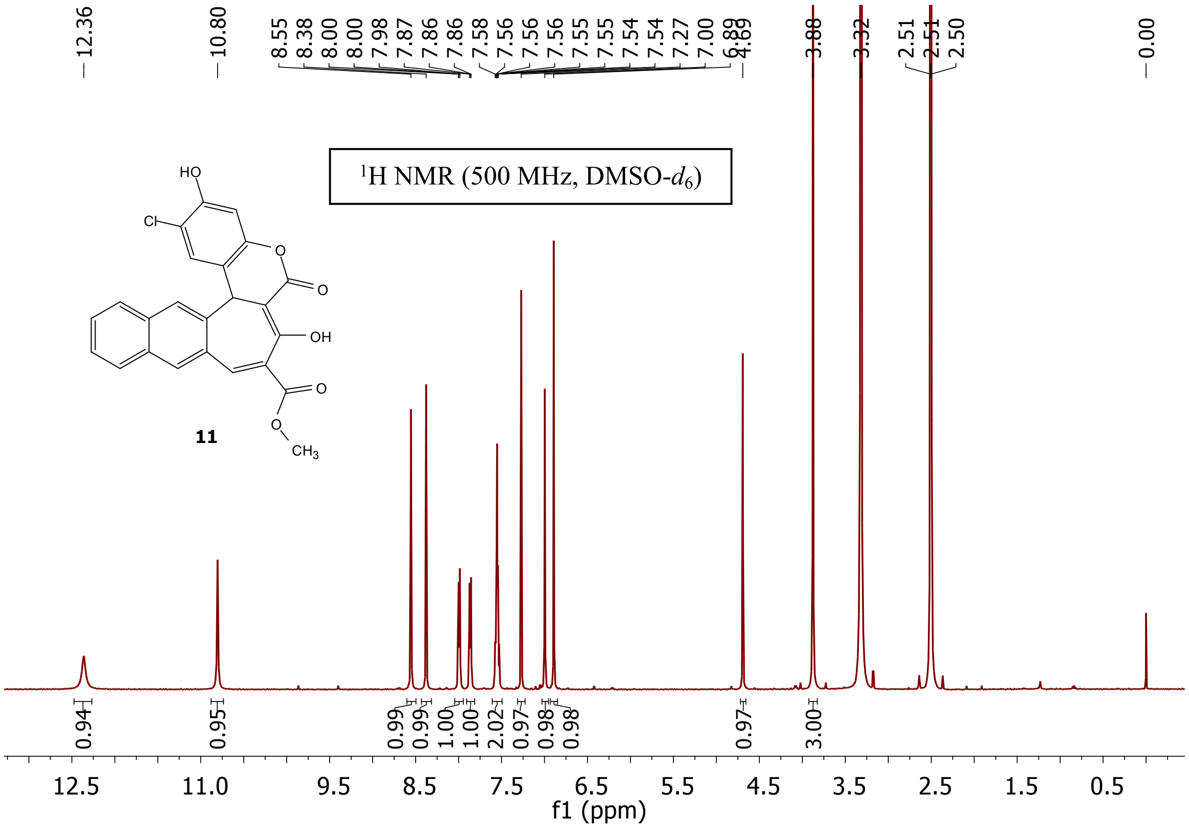
^{1}H NMR (500 MHz, DMSO-d_6)
12.36
10.80
8.55 8.38 8.00 8.00 7.98 7.87 7.86 7.86 7.58 7.56 7.56 7.56 7.55 7.55 7.54 7.54 7.27 7.00 6.89 4.69
3.88
3.32
2.51 2.51 2.50
0.00
HO
Cl
O
O
OH
O
O
CH3
11
0.94
0.95
0.99 0.99 1.00 1.00 2.02 0.97 0.98 0.98
0.97
3.00
12.5 11.0 9.5 8.5 7.5 6.5 5.5 4.5 3.5 2.5 1.5 0.5
f1 (ppm)

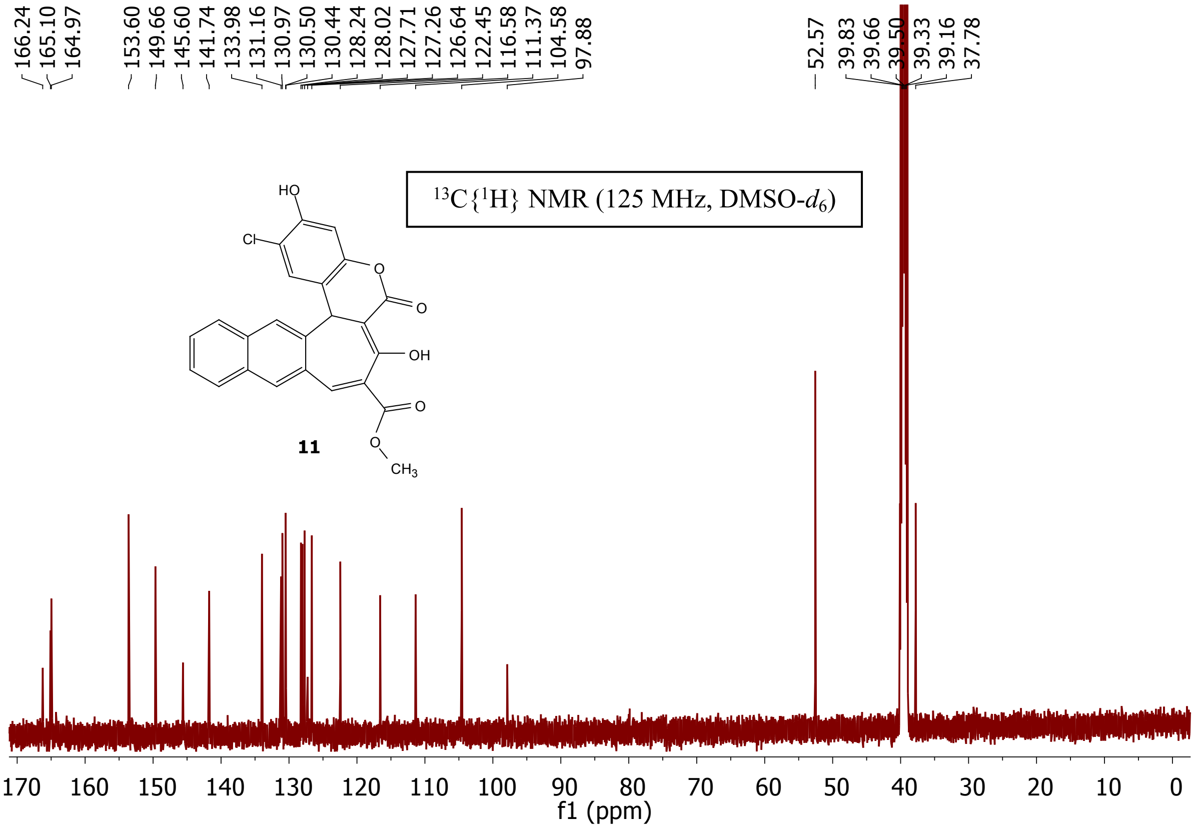
^{13}C{^{1}H} NMR (125 MHz, DMSO-d_6)
166.24 165.10 164.97
153.60 149.66 145.60 141.74 133.98 131.16 130.97 130.50 130.44 128.24 128.02 127.71 127.26 126.64 122.45 116.58 111.37 104.58 97.88
52.57
39.83 39.66 39.50 39.33 39.16 37.78
HO
Cl
O
O
OH
O
O
CH3
11
170 160 150 140 130 120 110 100 90 80 70 60 50 40 30 20 10 0
f1 (ppm)

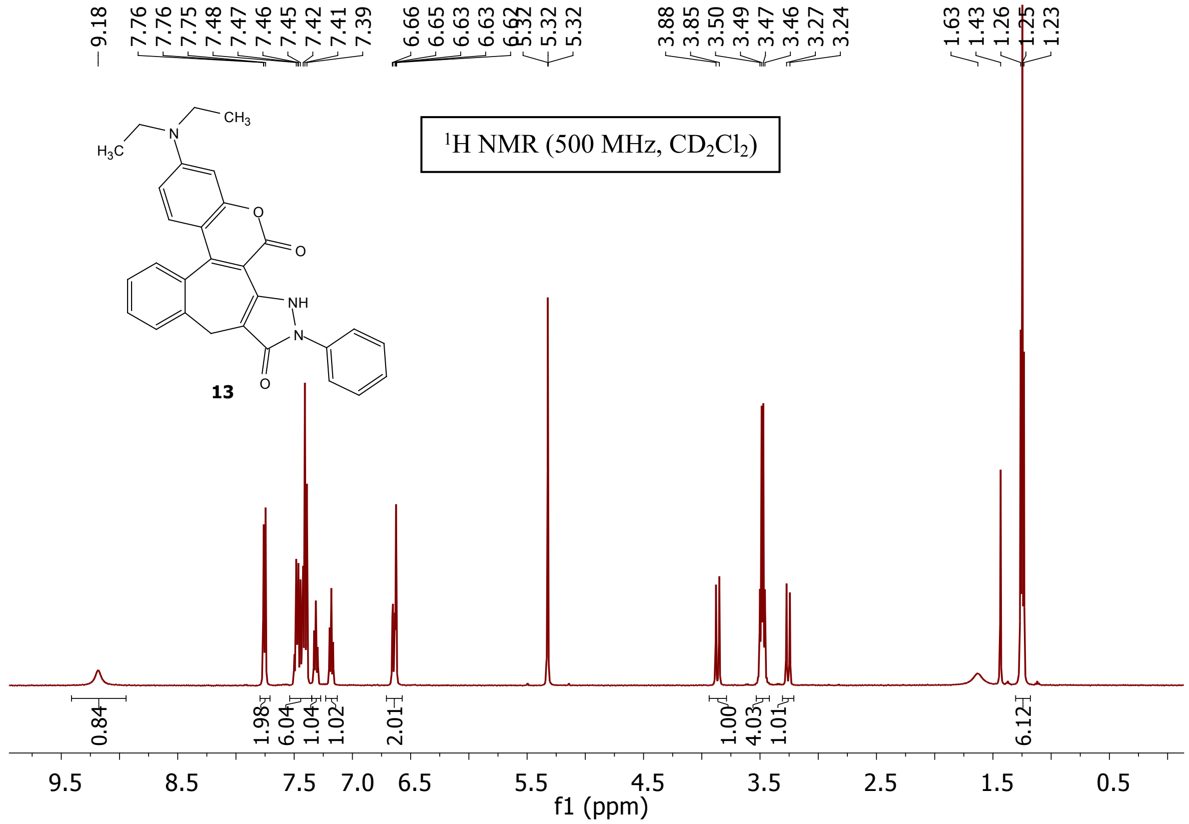

^{1}H NMR (500 MHz, CD_2Cl_2)
13
9.18
7.76
7.76
7.75
7.48
7.47
7.46
7.45
7.42
7.41
7.39
6.66
6.65
6.63
6.63
6.62
5.32
5.32
5.32
3.88
3.85
3.50
3.49
3.47
3.46
3.27
3.24
1.63
1.43
1.26
1.25
1.23
0.84
1.98
6.04
1.04
1.02
2.01
1.00
4.03
1.01
6.12
f1 (ppm)

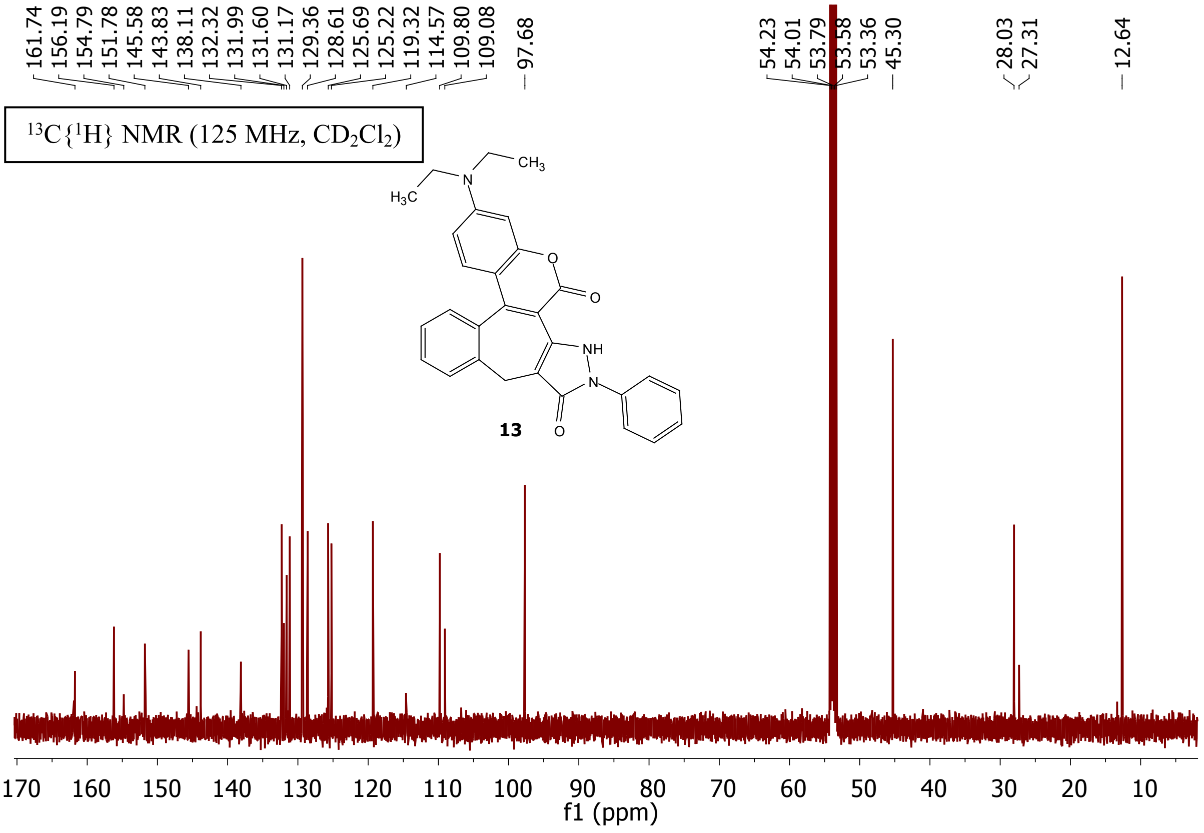

$^{13}C\{^{1}H\}$ NMR (125 MHz, CD_2Cl_2)
13
161.74
156.19
154.79
151.78
145.58
143.83
138.11
132.32
131.99
131.60
131.17
129.36
128.61
125.69
125.22
119.32
114.57
109.80
109.08
97.68
54.23
54.01
53.79
53.58
53.36
45.30
28.03
27.31
12.64
f1 (ppm)

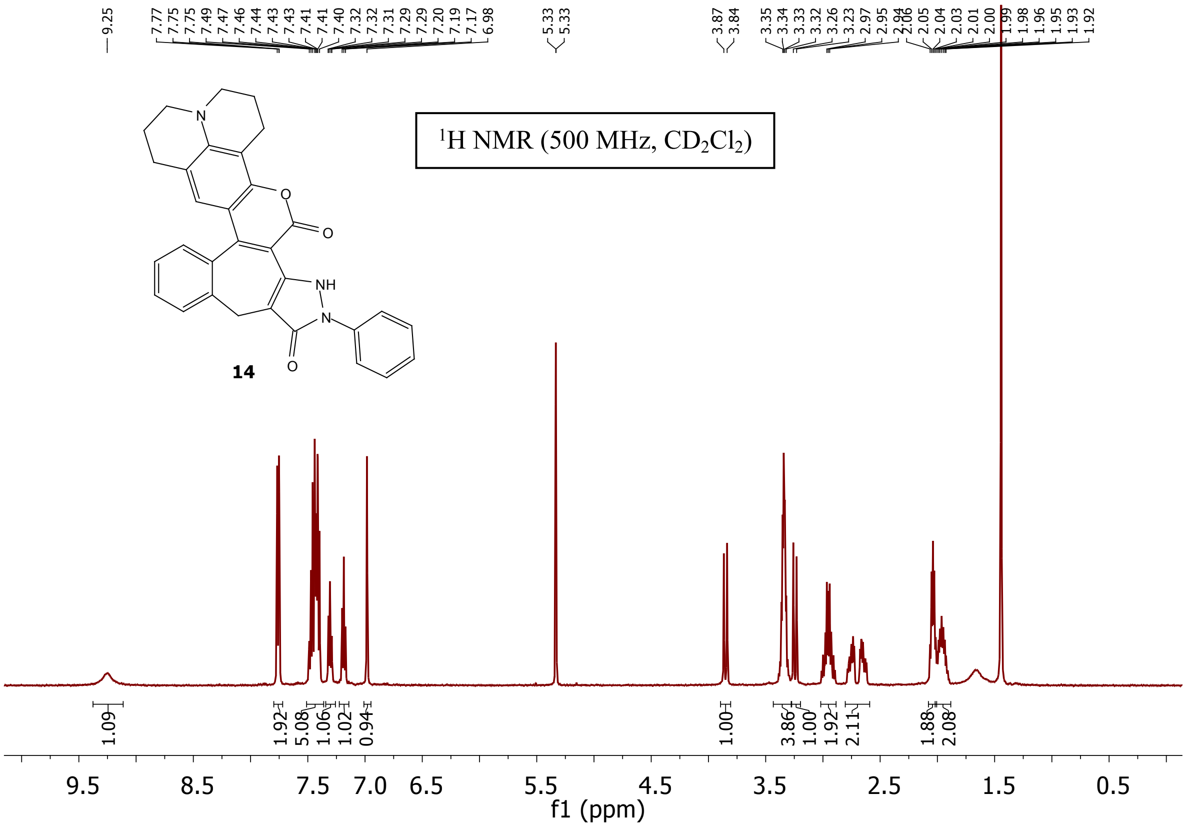

9.25
7.77 7.75 7.75 7.49 7.47 7.46 7.44 7.43 7.43 7.41 7.41 7.40 7.32 7.32 7.31 7.29 7.29 7.20 7.19 7.17 6.98
5.33 5.33
3.87 3.84
3.35 3.34 3.33 3.32 3.26 3.23 2.97 2.95 2.94 2.06 2.05 2.04 2.03 2.01 2.00 1.99 1.98 1.96 1.95 1.93 1.92
^{1}H NMR (500 MHz, CD_2Cl_2)
N
O
O
NH
N
O
14
1.09 1.92 5.08 1.06 1.02 0.94 1.00 3.86 1.00 1.92 2.11 1.88 2.08
9.5 8.5 7.5 7.0 6.5 5.5 4.5 3.5 2.5 1.5 0.5
f1 (ppm)

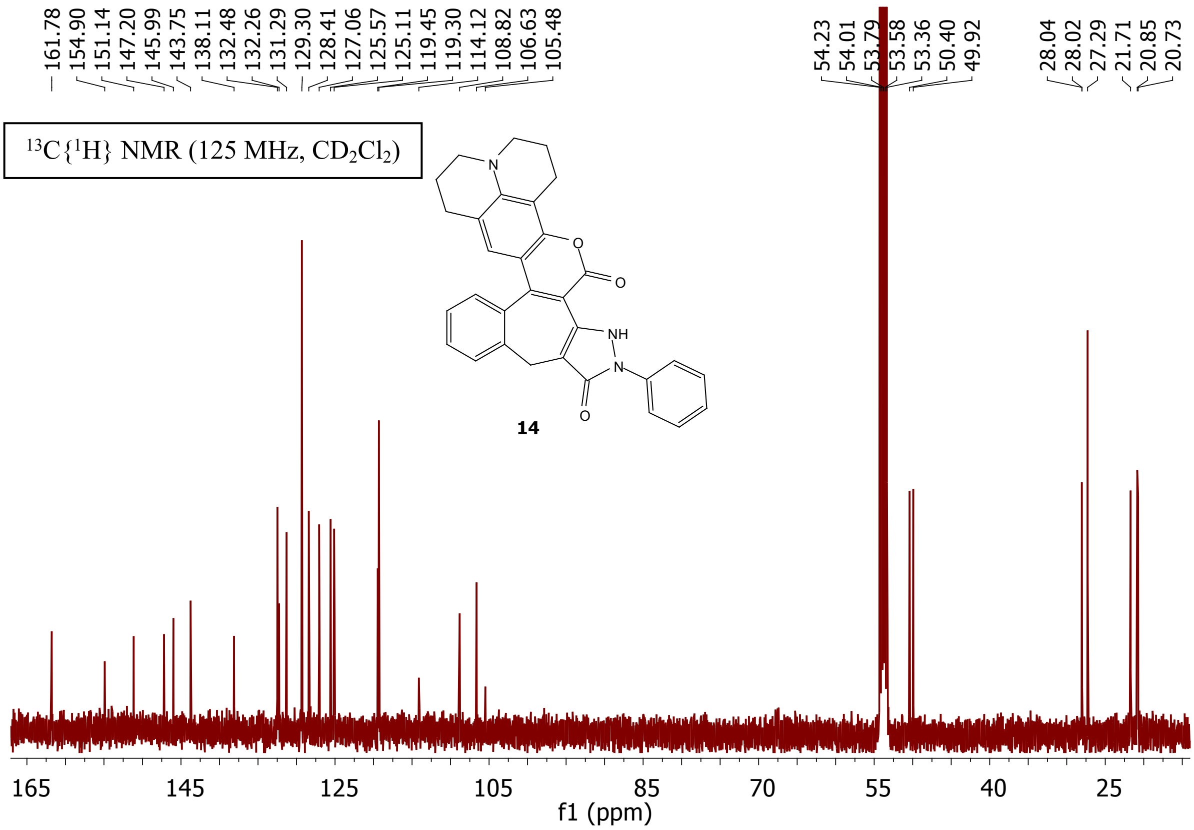

161.78 154.90 151.14 147.20 145.99 143.75 138.11 132.48 132.26 131.29 129.30 128.41 127.06 125.57 125.11 119.45 119.30 114.12 108.82 106.63 105.48
54.23 54.01 53.79 53.58 53.36 50.40 49.92
28.04 28.02 27.29 21.71 20.85 20.73
^{13}C{^{1}H} NMR (125 MHz, CD_2Cl_2)
N
O
O
NH
N
O
14
165 145 125 105 85 70 55 40 25
f1 (ppm)

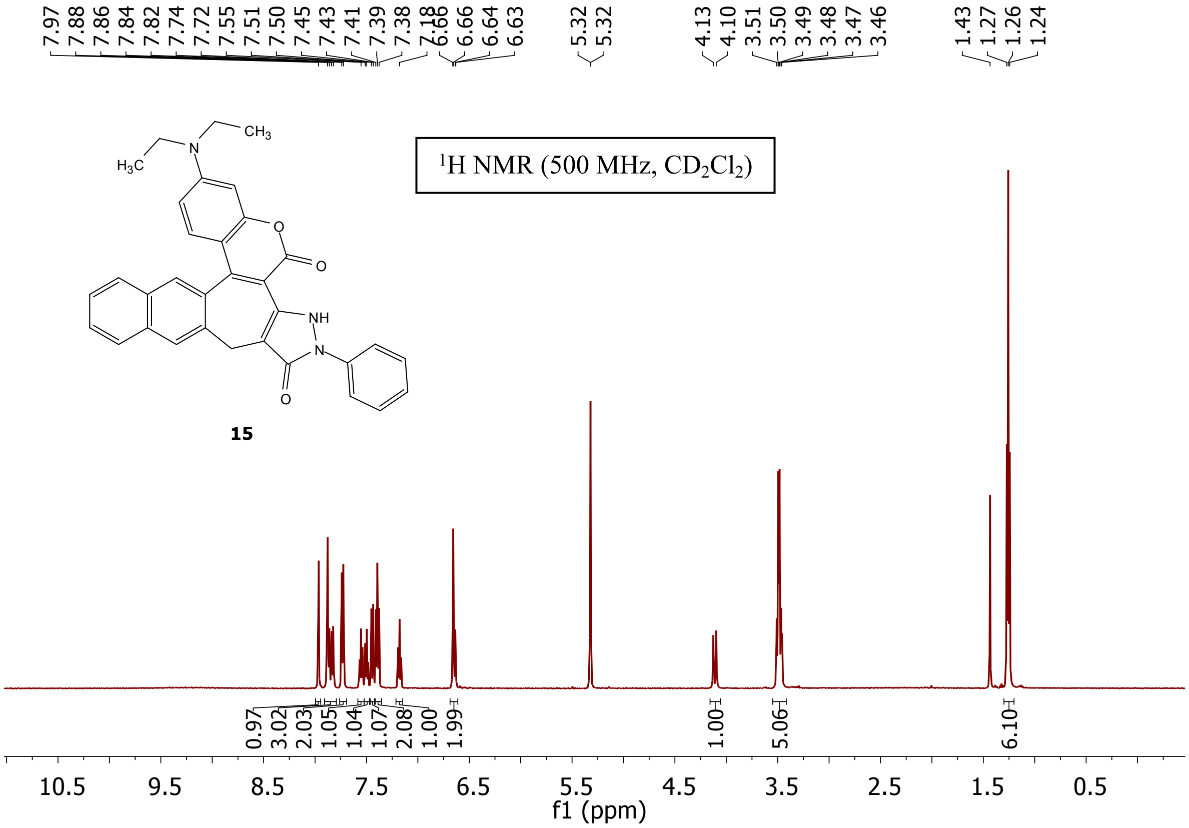
7.97
7.88
7.86
7.84
7.82
7.74
7.72
7.55
7.51
7.50
7.45
7.43
7.41
7.39
7.38
7.18
6.66
6.66
6.64
6.63
5.32
5.32
4.13
4.10
3.51
3.50
3.49
3.48
3.47
3.46
1.43
1.27
1.26
1.24
^{1}H NMR (500 MHz, CD_2Cl_2)
15
0.97
3.02
2.03
1.05
1.04
1.07
2.08
1.00
1.99
1.00
5.06
6.10
10.5
9.5
8.5
7.5
6.5
5.5
4.5
3.5
2.5
1.5
0.5
f1 (ppm)

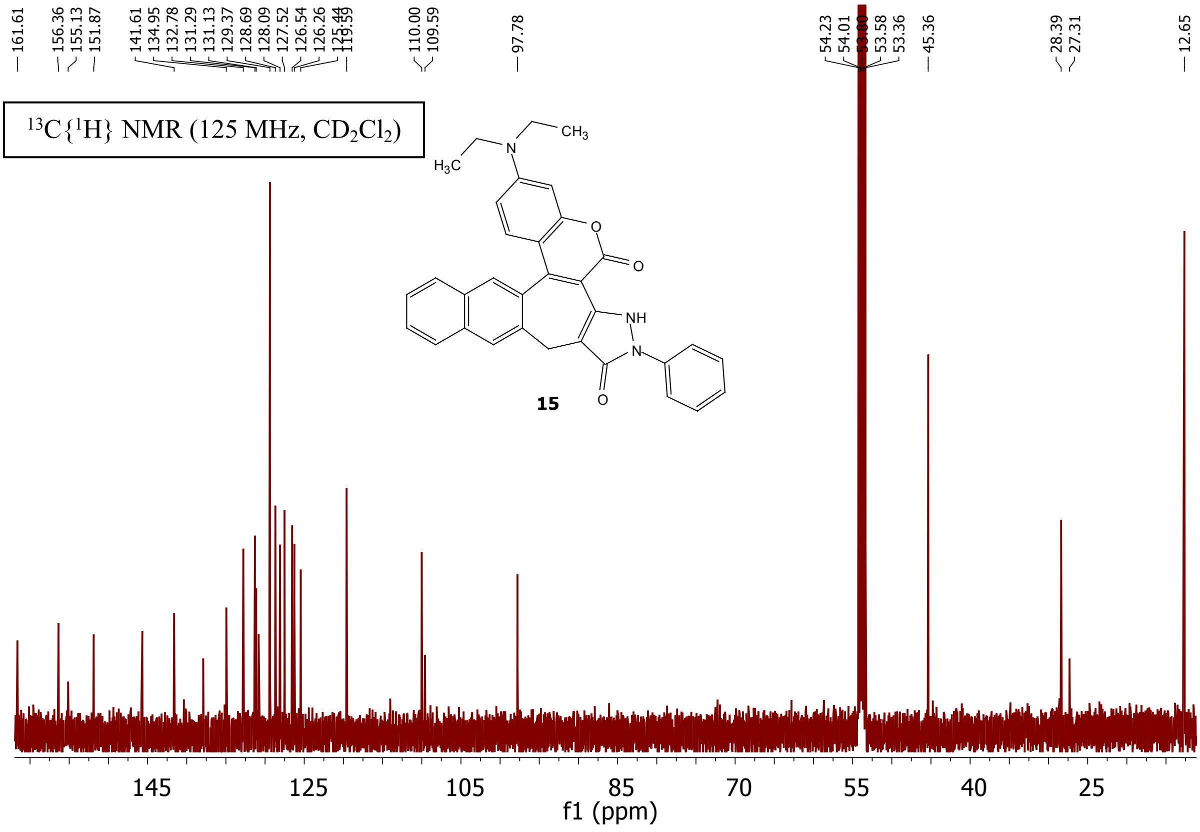
161.61
156.36
155.13
151.87
141.61
134.95
132.78
131.29
131.13
129.37
128.69
128.09
127.52
126.54
126.26
125.44
119.59
110.00
109.59
97.78
54.23
54.01
53.58
53.36
45.36
28.39
27.31
12.65
^{13}C{^{1}H} NMR (125 MHz, CD_2Cl_2)
15
145
125
105
85
70
55
40
25
f1 (ppm)

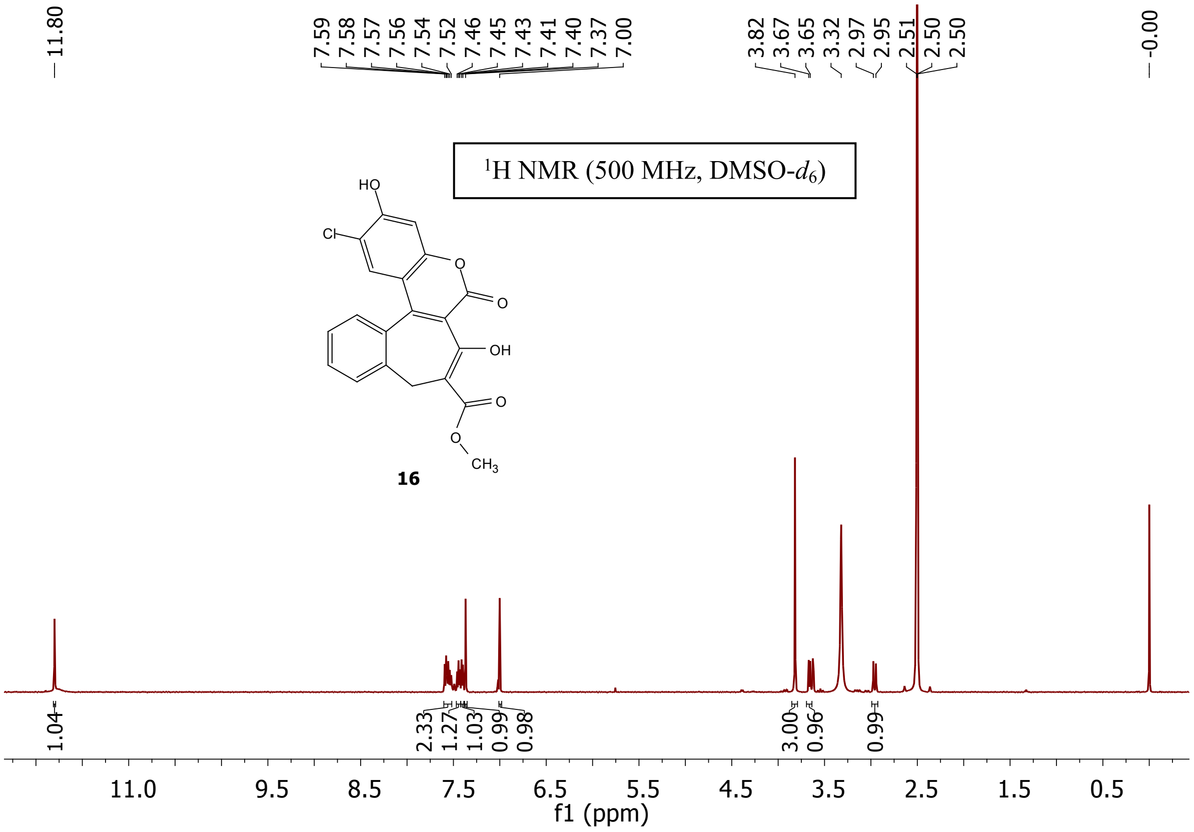

11.80
7.59 7.58 7.57 7.56 7.54 7.52 7.46 7.45 7.43 7.41 7.40 7.37 7.00
3.82 3.67 3.65 3.32 2.97 2.95 2.51 2.50 2.50
-0.00
¹H NMR (500 MHz, DMSO-d6)
HO
Cl
O
O
OH
O
O
CH3
16
1.04
2.33 1.27 1.03 0.99 0.98
3.00 0.96
0.99
11.0 9.5 8.5 7.5 6.5 5.5 4.5 3.5 2.5 1.5 0.5
f1 (ppm)

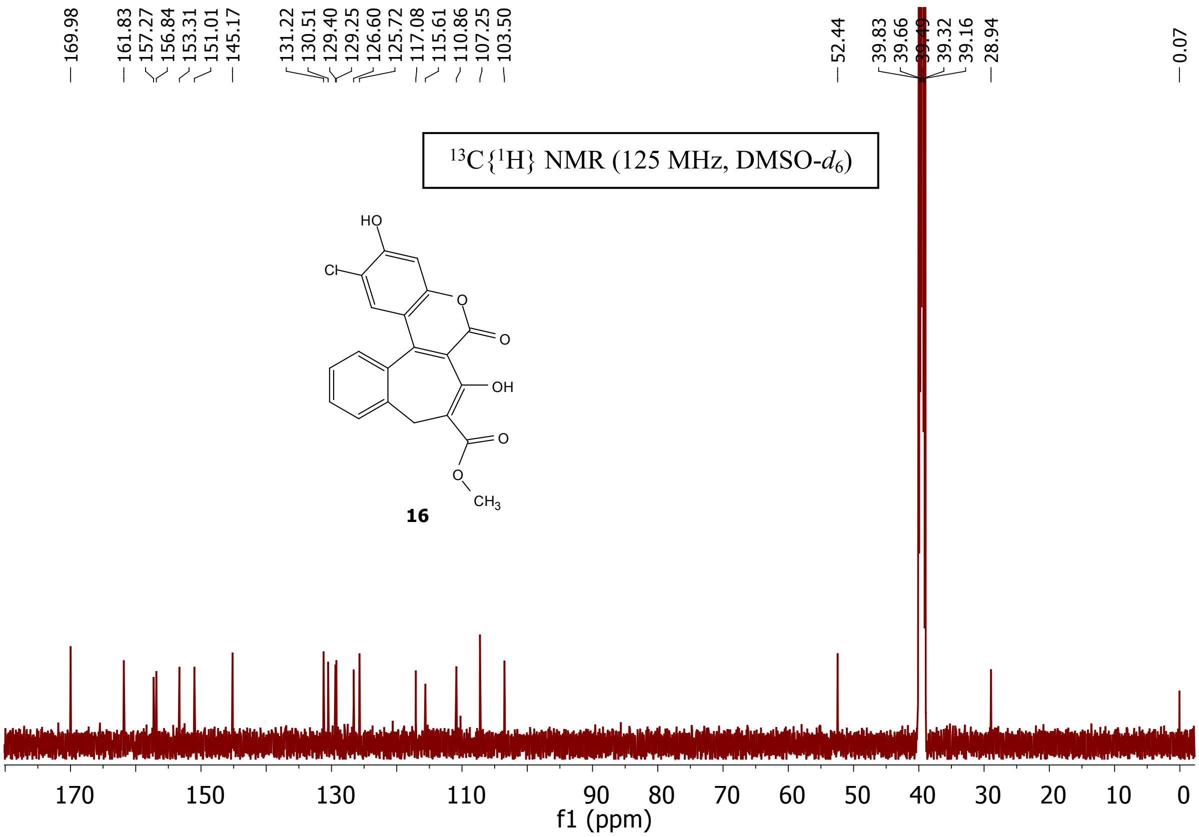

169.98
161.83 157.27 156.84 153.31 151.01 145.17
131.22 130.51 129.40 129.25 126.60 125.72 117.08 115.61 110.86 107.25 103.50
52.44
39.83 39.66 39.49 39.32 39.16 28.94
-0.07
¹³C{¹H} NMR (125 MHz, DMSO-d6)
HO
Cl
O
O
OH
O
O
CH3
16
170 150 130 110 90 80 70 60 50 40 30 20 10 0
f1 (ppm)

5. Cartesian Coordinates

5.1. Molecular structures obtained at MP2/cc-pVDZ level of theory.

3ab				*3ac*			
O	0.98455063	-2.72864027	-0.57931865	O	0.98190562	-2.71662395	0.62782118
C	-0.36797484	-2.64344606	-0.78834413	C	-0.41110896	-2.66470908	0.57187004
C	-2.02634133	1.47712081	0.34401799	C	-1.96675418	1.48196981	-0.50591826
C	-1.05499019	-1.36106321	-0.52294399	C	-1.02474556	-1.31612874	0.50092388
C	-2.53515401	-1.38418992	-0.63470386	C	-2.49135968	-1.27340651	0.66657646
C	-3.31234491	-0.55693806	0.15068581	C	-3.27917496	-0.51684614	-0.18057268
C	-2.64976087	0.36765575	1.14647968	C	-2.63229486	0.34322809	-1.24044535
C	1.72299455	-1.63183028	-0.20140338	C	1.75188876	-1.61498213	0.36162249
C	-0.29036096	-0.21053393	-0.32993119	C	-0.25252474	-0.17993956	0.30765101
C	1.13967565	-0.35375600	-0.10148665	C	1.18744022	-0.33777126	0.15820995
C	3.07637113	-1.87806469	0.04868940	C	3.12851250	-1.85198927	0.27302996
C	2.00006554	0.69576214	0.31351869	C	2.08274658	0.69745526	-0.21228441
C	3.92896426	-0.82460995	0.45091465	C	4.02129078	-0.80189226	-0.04010184
C	3.35039406	0.47411751	0.57296597	C	3.45457151	0.47698720	-0.31747882
H	3.42540954	-2.90405119	-0.07660450	H	3.46225602	-2.87663815	0.44291461
H	1.58782978	1.69878015	0.45195629	H	1.68357402	1.68739295	-0.44925243
H	3.95924656	1.32206656	0.89193681	H	4.08934275	1.31136770	-0.62093624
N	5.26635787	-1.05037760	0.74540455	N	5.39597709	-1.00810065	-0.07574835
C	6.16300979	0.09681665	0.78181452	C	6.21256991	-0.03703378	-0.79033249
H	7.17699305	-0.25948852	1.01265009	H	7.26520494	-0.34685831	-0.72023874
H	6.19113345	0.65310380	-0.17719707	H	5.93957542	0.05427461	-1.86176508
H	5.87045941	0.79592726	1.58113449	H	6.13096946	0.95761400	-0.32463061
C	5.84899912	-2.31529155	0.32235018	C	5.87168786	-2.38331773	-0.10198134
H	6.90069760	-2.34292046	0.64078164	H	6.97111174	-2.37534124	-0.10120143
H	5.33315378	-3.15889849	0.80802734	H	5.54274378	-2.92075598	0.80135130
H	5.80370040	-2.46487688	-0.77592337	H	5.51888471	-2.94669358	-0.99046216
O	-0.91780161	-3.64991627	-1.22310010	O	-1.01240588	-3.71440188	0.59672275
O	-3.09491842	-2.23069244	-1.52630493	O	-2.96381719	-2.00362947	1.68377538
H	-2.41500305	-2.92412504	-1.68139895	H	-3.94959144	-1.88323956	1.63958171
C	-0.86212079	1.15137330	-0.39240734	C	-0.83045596	1.18564818	0.29105654
C	-4.78581041	-0.49194846	-0.03877410	C	-4.71573165	-0.45744250	0.09471616
O	-5.48465952	-1.23177273	-0.70377666	O	-5.29387534	-1.11606317	0.96837869
O	-5.30060099	0.57707400	0.65690875	O	-5.37905253	0.40579799	-0.70667142
C	-6.72110842	0.71011814	0.51007879	C	-6.79295019	0.47124568	-0.44888072
H	-6.99448117	0.86103020	-0.54582750	H	-7.26127613	-0.51327236	-0.59764753
H	-7.24120790	-0.18734335	0.87949683	H	-6.98469438	0.80373724	0.58234211
H	-6.99841348	1.58814602	1.10822210	H	-7.18580119	1.19931839	-1.16953284
C	-2.58916957	2.76167753	0.22727309	C	-2.49889955	2.78481015	-0.49730150
C	-1.99749328	3.72570842	-0.60304937	C	-1.92189985	3.79279209	0.29101227
C	-0.27114495	2.12600486	-1.23301002	C	-0.26101660	2.20348825	1.09279921
C	-0.84216382	3.40204490	-1.34028248	C	-0.80876393	3.49474608	1.09811142
H	0.61001532	1.86380961	-1.82836228	H	0.59245295	1.96540174	1.73620630
H	-0.38959115	4.14417848	-2.00637194	H	-0.36793422	4.26850223	1.73545714
H	-2.43963117	4.72433045	-0.68575419	H	-2.34546020	4.80279341	0.28743531
H	-3.50197704	2.99174542	0.78727183	H	-3.38292893	2.99900956	-1.10867126
H	-1.86601371	-0.17576869	1.70452525	H	-3.38727952	0.71387760	-1.94759856
H	-3.38978389	0.75195209	1.85917191	H	-1.88290898	-0.24438091	-1.79991182

4ab				*4ac*			
O	0.32059443	-2.60263915	-0.66208927	O	0.2752347	-2.6352561	-0.4354900
C	-1.03567189	-2.52814543	-0.85555810	C	-1.1189869	-2.5639737	-0.4622915
C	-2.68668657	1.61212076	0.20428341	C	-2.6482684	1.6598169	0.3022368
C	-1.72165811	-1.24386443	-0.60947376	C	-1.7113579	-1.2066725	-0.4959213
C	-3.20278575	-1.27262116	-0.69965202	C	-3.1676107	-1.1504481	-0.7311260
C	-3.97138198	-0.42811604	0.07623093	C	-3.9790659	-0.3343737	0.0352602
C	-3.29674169	0.52058126	1.04087136	C	-3.3646577	0.5765078	1.0719455
C	1.06439583	-1.49743914	-0.31356961	C	1.0528329	-1.5332257	-0.1839574
C	-0.95505490	-0.08775560	-0.45183813	C	-0.9271837	-0.0729588	-0.3293352
C	0.47657056	-0.21907304	-0.23784964	C	0.4987806	-0.2380987	-0.0938542
C	2.42177177	-1.74797329	-0.06054869	C	2.4217201	-1.7962321	-0.0063979
C	1.32994119	0.84656598	0.14360489	C	1.3850541	0.8128824	0.2472295
C	3.25658679	-0.65354733	0.28695338	C	3.2909161	-0.7146747	0.2930334
C	2.69004753	0.65644563	0.39137880	C	2.7525192	0.6025715	0.4325082
H	0.90681765	1.84866572	0.26503804	H	0.9860767	1.8231174	0.3827424
N	4.60495345	-0.86889811	0.57422319	N	4.6509906	-0.9517555	0.5084056
C	5.48932932	0.29014117	0.59919458	C	5.5497788	0.1952999	0.4757103
C	5.20017538	-2.10755177	0.08502076	C	5.1964863	-2.1965877	-0.0205400
H	6.20372337	-2.19233614	0.53457597	H	6.2263305	-2.2956301	0.3619134
H	5.32750696	-2.08584058	-1.02090465	H	5.2505821	-2.1757271	-1.1326908
O	-1.58521961	-3.54754336	-1.26147437	O	-1.7314154	-3.6079498	-0.4670478

O	-3.77282791	-2.14367186	-1.56055085	O	-3.6086498	-1.9311947	-1.7247702
H	-3.09077191	-2.83627575	-1.71123597	H	-4.5930417	-1.7929202	-1.7314494
C	-1.53226809	1.27082474	-0.54040799	C	-1.4836724	1.2988968	-0.4239144
C	-5.44683042	-0.36964830	-0.09516052	C	-5.4003395	-0.2687226	-0.3078377
O	-6.15291581	-1.12266087	-0.73757981	O	-5.9514848	-0.9676781	-1.1676994
O	-5.95559275	0.71093294	0.58765415	O	-6.0831483	0.6498338	0.4119978
C	-7.37795840	0.83802691	0.45622315	C	-7.4825461	0.7221086	0.0866579
H	-7.66508359	0.96880950	-0.59874104	H	-7.6221928	0.9959009	-0.9698888
H	-7.89138043	-0.05357075	0.84856021	H	-7.9751913	-0.2443555	0.2711958
H	-7.65003730	1.72632345	1.04152678	H	-7.8937753	1.4976235	0.7448998
C	-3.25411633	2.89240324	0.06479707	C	-3.1584473	2.9666744	0.1902166
C	-2.67689005	3.83725423	-0.79721162	C	-2.5321221	3.9156452	-0.6330153
C	-0.95625958	2.22588970	-1.41324954	C	-0.8654212	2.2564069	-1.2628885
C	-1.53179090	3.49784046	-1.54329323	C	-1.3913891	3.5522218	-1.3719245
H	-0.08275250	1.95062046	-2.01393244	H	0.0101726	1.9661530	-1.8529260
H	-1.09081173	4.22435018	-2.23399826	H	-0.9121142	4.2782605	-2.0371351
H	-3.12277591	4.83257894	-0.89792583	H	-2.9388545	4.9296454	-0.7100744
H	-4.15924680	3.13414845	0.63235696	H	-4.0644136	3.2303670	0.7477963
H	-2.50402951	-0.00852557	1.60008380	H	-2.6505555	0.0117411	1.6971838
H	-4.02747638	0.92003012	1.75487550	H	-4.1434551	0.9990486	1.7218887
C	3.55728553	1.84699212	0.75441294	C	3.6539852	1.7795894	0.7548160
C	4.85561089	1.40295782	1.42741620	C	4.9850636	1.3144508	1.3447241
H	3.80057646	2.41069079	-0.16642207	H	3.8491212	2.3515235	-0.1725178
H	2.98764511	2.53326253	1.40459819	H	3.1328938	2.4652106	1.4454450
H	4.64976392	1.01200669	2.43931964	H	4.8364153	0.9216935	2.3659178
H	5.55613238	2.24946279	1.52411593	H	5.7025847	2.1504851	1.4009724
H	6.44492089	-0.03464298	1.04439412	H	6.5284833	-0.1417143	0.8572551
H	5.70807144	0.65886696	-0.42863275	H	5.7056930	0.5642221	-0.5637745
C	4.32325369	-3.28827232	0.48815757	C	4.3307641	-3.3646506	0.4397287
C	2.95146698	-3.16150787	-0.17502677	C	2.9213890	-3.2196013	-0.1349521
H	4.21786515	-3.28033349	1.58706568	H	4.2963515	-3.3527020	1.5431598
H	4.80587903	-4.23722350	0.20021131	H	4.7808617	-4.3210636	0.1246022
H	3.01775793	-3.43669095	-1.24455720	H	2.9135874	-3.5088484	-1.2028508
H	2.22837320	-3.86242478	0.27373963	H	2.2146921	-3.9008827	0.3668282
	5ab				***5ac***		
O	1.98546009	-2.06698527	-1.49467209	O	2.11245776	-1.93664484	-1.57710941
C	0.61489696	-2.12335041	-1.53763498	C	0.72004378	-2.05424328	-1.71298754
C	-1.03716720	1.67184726	-0.37229327	C	-1.00776820	1.64700443	-0.49633509
C	-0.15787986	-1.25402734	-0.65868692	C	-0.10953276	-1.27150269	-0.77153691
C	-1.53637709	-1.33676558	-0.72174008	C	-1.48240555	-1.41596802	-0.77095533
C	-2.40477489	-0.42542910	0.04198124	C	-2.34176325	-0.46489735	-0.03044797
C	-2.17502642	0.92023971	0.15774355	C	-2.12436077	0.89219326	0.05449576
C	2.61157562	-1.42200615	-0.43792898	C	2.66821134	-1.37260990	-0.45221595
C	0.49210126	-0.22715098	0.23209980	C	0.50830826	-0.23161764	0.13152295
C	1.94027328	-0.56172348	0.44431850	C	1.94290810	-0.55179986	0.42428402
C	3.97579682	-1.69989301	-0.29927810	C	4.02224353	-1.65423727	-0.22993546
C	2.68002698	0.02035646	1.48715467	C	2.60518135	-0.00978970	1.53745507
C	4.71567479	-1.10789569	0.73364420	C	4.68894270	-1.10534303	0.87415662
C	4.04399569	-0.24747942	1.63033137	C	3.95832624	-0.28116723	1.75832279
H	2.18595266	0.69896544	2.18867123	H	2.06226571	0.63625849	2.23375587
O	0.12139033	-2.91512279	-2.33968427	O	0.32199324	-2.75372701	-2.61333048
O	-2.19959060	-2.18998796	-1.50433708	O	-2.08716420	-2.39530149	-1.46736327
H	-1.48481972	-2.68845153	-1.98931766	H	-3.02627854	-2.39592206	-1.17583880
C	0.28007267	1.14202067	-0.39516700	C	0.30822763	1.11754858	-0.52689366
C	-3.63636237	-1.03872291	0.64410670	C	-3.66339854	-0.97765891	0.44910157
O	-3.73537133	-2.21190231	0.94314050	O	-4.23672457	-1.95511554	-0.02593194
O	-4.60814971	-0.11799165	0.85322980	O	-4.17641886	-0.24783213	1.44932984
C	-5.77025284	-0.65489256	1.50624340	C	-5.48475175	-0.67684917	1.87820606
H	-6.22182038	-1.45119851	0.89757890	H	-6.19981243	-0.62356624	1.04529600
H	-5.50761420	-1.06820494	2.49093477	H	-5.44443287	-1.70916275	2.25205685
H	-6.46193001	0.18866302	1.61311679	H	-5.76598280	0.01574794	2.67890830
C	-1.26332539	2.98046646	-0.86159704	C	-1.24402270	2.94896468	-1.00277329
C	-0.22109706	3.71858784	-1.43240104	C	-0.20800063	3.67304649	-1.59952133
C	1.32073622	1.89515364	-0.96746347	C	1.34345940	1.85669306	-1.12618284
C	1.07316596	3.17074427	-1.49462711	C	1.08679545	3.12245490	-1.67012928
H	2.33463882	1.48402087	-0.98093135	H	2.35690446	1.44372150	-1.13952078
H	1.89296290	3.74611624	-1.93504160	H	1.89970342	3.69247326	-2.12996826
H	-0.41349550	4.72146799	-1.82456577	H	-0.40304664	4.66946125	-2.00653111
H	-2.27449634	3.39849902	-0.81570422	H	-2.25432494	3.36867720	-0.95202055
H	-2.97288523	1.50646608	0.62740001	H	-2.93347233	1.48224118	0.50030044
O	6.03699570	-1.40099043	0.82673030	O	6.00330590	-1.40186851	1.04438429
H	6.37894859	-0.92307603	1.60036750	H	6.28990012	-0.95306359	1.85675840
Cl	4.95810443	0.47909177	2.93075388	Cl	4.78332684	0.39370044	3.14534271
H	4.46822470	-2.38218881	-0.99567772	H	4.56460996	-2.30355989	-0.92086635
H	-0.02855834	-0.22726981	1.20920265	H	-0.05345284	-0.21673849	1.08471811

9ab				9ac			
O	1.00927423	-3.37084839	-0.76359903	O	0.99097198	-3.43724554	-0.50723520
C	-0.34832681	-3.22622782	-0.89739594	C	-0.39917604	-3.32858845	-0.45514590
C	-1.79830452	0.85835392	0.63214028	C	-1.78470700	0.86402374	0.71010078
C	-0.97179277	-1.94184821	-0.51606022	C	-0.95532940	-1.95762432	-0.36836305
C	-2.45689166	-1.90360576	-0.55932348	C	-2.42025261	-1.84916712	-0.52775394
C	-3.16460824	-1.11712932	0.32531460	C	-3.17131761	-1.07915206	0.33951905
C	-2.42573829	-0.29852041	1.36079554	C	-2.48809271	-0.26911527	1.41683464
C	1.80566698	-2.32771767	-0.35362836	C	1.80539080	-2.37008653	-0.23341848
C	-0.15814345	-0.83637194	-0.27657102	C	-0.13858006	-0.85657593	-0.16266659
C	1.27450256	-1.04230258	-0.12827930	C	1.29404811	-1.07224432	-0.01711640
C	3.15776252	-2.63834914	-0.18058093	C	3.17151650	-2.66342561	-0.15199105
C	2.18979248	-0.05813239	0.32836634	C	2.23250565	-0.07675089	0.35654255
C	4.07077788	-1.64048930	0.23132695	C	4.10746781	-1.65304895	0.16602044
C	3.54084186	-0.34444100	0.50781088	C	3.59430631	-0.35425334	0.45537881
H	1.81744088	0.93908265	0.57784246	H	1.87484015	0.92692637	0.60194604
O	-0.95674477	-4.18151995	-1.36800454	O	-1.04391557	-4.35185694	-0.49612828
O	-3.08430843	-2.65346009	-1.49120757	O	-2.92486974	-2.53403460	-1.56013005
H	-2.44216424	-3.36101567	-1.72319686	H	-3.90443435	-2.36965063	-1.51227922
C	-0.68005085	0.54702714	-0.20494849	C	-0.66271647	0.53151865	-0.12155452
C	-4.64087598	-0.98325544	0.20982012	C	-4.60309071	-0.94618294	0.06648453
O	-5.39541265	-1.63431180	-0.48656507	O	-5.21093367	-1.55695700	-0.82184359
O	-5.08419527	0.03605053	1.02007297	O	-5.22563482	-0.07034890	0.88669782
C	-6.50291664	0.23352837	0.94707639	C	-6.63417001	0.06936465	0.62879534
H	-6.81148819	0.49021002	-0.07827729	H	-6.80703757	0.43470408	-0.39461814
H	-7.04369227	-0.67435952	1.25646729	H	-7.14990593	-0.89452052	0.75400403
H	-6.72083159	1.06277024	1.63303719	H	-6.99301857	0.79868037	1.36576151
C	-2.30449992	2.15404745	0.66033324	C	-2.25510275	2.17313269	0.74293263
C	-1.72136490	3.19155874	-0.11952334	C	-1.64949264	3.20256985	-0.03141719
C	-0.09407029	1.54907843	-0.98809609	C	-0.06389941	1.52443140	-0.90481745
C	-0.59890694	2.87789446	-0.97124778	C	-0.54007719	2.86436363	-0.88873076
H	0.74300989	1.30469196	-1.65339009	H	0.77031232	1.26454688	-1.56741968
H	-3.17610610	2.37507651	1.28828251	H	-3.12243136	2.41530096	1.37002944
H	-1.63791759	-0.91099737	1.83547214	H	-1.75890548	-0.89686269	1.95892030
H	-3.12021588	0.04606208	2.13695855	H	-3.22636564	0.11163343	2.13631316
H	3.45961780	-3.66743423	-0.38065082	H	3.46282900	-3.69913927	-0.33257410
C	-0.02318614	3.91212463	-1.76997649	C	0.05587030	3.88594887	-1.68820133
C	-2.21930129	4.52832518	-0.09980482	C	-2.11685507	4.54978525	-0.00591492
C	-0.53211148	5.20361766	-1.72712861	C	-0.42305323	5.18905278	-1.64078394
C	-1.63753373	5.51532051	-0.88554334	C	-1.51596297	5.52487696	-0.79267017
H	0.82679443	3.66933376	-2.41853421	H	0.89705770	3.62460767	-2.34093258
H	-0.08202219	5.98828082	-2.34463087	H	0.04229571	5.96433786	-2.25881197
H	-2.03153926	6.53689136	-0.86166205	H	-1.88508330	6.55558211	-0.76384207
H	-3.07288442	4.76447203	0.54648843	H	-2.96078577	4.80484188	0.64597479
N	5.42627097	-1.90962907	0.35476669	N	5.47236600	-1.91535459	0.19493800
C	5.84357844	-3.30394167	0.36684531	C	5.89234695	-3.30878627	0.20846921
H	6.93904622	-3.34301291	0.44870796	H	6.99117497	-3.34513441	0.20390043
H	5.40166202	-3.87899962	1.20620800	H	5.51996421	-3.86450509	1.09373647
H	5.56321440	-3.79804551	-0.57700229	H	5.53905576	-3.82531112	-0.69787766
C	6.24463091	-0.98189952	1.12303981	C	6.33141295	-0.98236610	0.91047148
H	5.91493124	-0.88541725	2.17758026	H	6.06772550	-0.88617061	1.98376802
H	7.28389643	-1.34001178	1.11033082	H	7.37018398	-1.33434886	0.83314568
H	6.23520707	0.01838035	0.66217440	H	6.28791621	0.01742642	0.45069007
H	4.19022759	0.45111655	0.87756976	H	4.26363881	0.45115237	0.76286299

11ab				11ac			
O	1.81880052	-2.81278149	-1.11889703	O	1.94482238	-2.67435364	-1.20581776
C	0.44687677	-2.86985123	-1.15584050	C	0.55039633	-2.79077856	-1.33833339
C	-1.20849474	0.95825275	-0.05931664	C	-1.17525724	0.94156564	-0.14544158
C	-0.32456691	-1.98529637	-0.28937666	C	-0.27793186	-1.99569528	-0.40432170
C	-1.70362683	-2.06548419	-0.35609414	C	-1.65133331	-2.13303217	-0.41119257
C	-2.57617631	-1.13801724	0.38942191	C	-2.51475174	-1.17370226	0.32243127
C	-2.34788700	0.20995077	0.48046662	C	-2.29618579	0.18297884	0.40180910
C	2.44886175	-2.14877582	-0.07538708	C	2.50347927	-2.10032328	-0.08666296
C	0.33033796	-0.93865316	0.57902921	C	0.34495176	-0.94634233	0.48951182
C	1.77990987	-1.27260552	0.79484361	C	1.78015333	-1.27051329	0.78488328
C	3.81489473	-2.42423458	0.06319965	C	3.85909685	-2.38140771	0.13524845
C	2.52368210	-0.67156272	1.82594888	C	2.44572546	-0.71912031	1.89333696
C	4.55882195	-1.81345847	1.08379727	C	4.52911157	-1.82315977	1.23407549
C	3.88940671	-0.93702885	1.96869361	C	3.80022600	-0.99029581	2.11365013
H	2.03181477	0.02088628	2.51691870	H	1.90480659	-0.06472882	2.58493311
O	-0.04832953	-3.67515380	-1.94461863	O	0.15083179	-3.49913262	-2.23188345
O	-2.36712482	-2.92989791	-1.12618833	O	-2.26021266	-3.10933675	-1.10919729
H	-1.65110748	-3.43782273	-1.60174044	H	-3.19860029	-3.10971210	-0.81361508
C	0.12494283	0.42299892	-0.07073327	C	0.15608044	0.40738904	-0.16805250
C	-3.80477084	-1.74339949	1.00806259	C	-3.83582082	-1.68549298	0.80845017

O	-3.90138681	-2.91292245	1.32447600	O	-4.40712354	-2.66928353	0.34240338
O	-4.77711271	-0.81982899	1.20898991	O	-4.35114140	-0.94699697	1.80261358
C	-5.93561811	-1.34934262	1.87690065	C	-5.66062356	-1.37486965	2.23339556
H	-6.39041234	-2.15495133	1.28120559	H	-6.37486030	-1.33019080	1.39795441
H	-5.66630112	-1.74915505	2.86645282	H	-5.61889800	-2.40459943	2.61699022
H	-6.62773199	-0.50391576	1.97621834	H	-5.94415538	-0.67464884	3.02800516
C	-1.43622029	2.24851351	-0.55595780	C	-1.41231313	2.23230010	-0.64060525
C	-0.39441819	3.00939380	-1.14885320	C	-0.37490745	2.98941402	-1.24366868
C	1.15986169	1.15855945	-0.64001292	C	1.18780569	1.13916783	-0.74694877
C	0.93266239	2.44589461	-1.20263315	C	0.95317681	2.42518276	-1.30806204
H	2.17862808	0.75278840	-0.63869597	H	2.20636512	0.73209234	-0.74711457
H	-2.44934950	2.66935711	-0.51831448	H	-2.42520533	2.65326634	-0.59590492
H	-3.14627140	0.80412902	0.94158647	H	-3.10478506	0.77585568	0.84740006
O	5.88156804	-2.10470924	1.17714540	O	5.84461735	-2.11958809	1.40413408
H	6.22556833	-1.61427235	1.94261731	H	6.13251584	-1.66521723	2.21353639
Cl	4.80919350	-0.18687282	3.25438595	Cl	4.62975173	-0.30382296	3.49492291
H	4.30544031	-3.11932386	-0.62335718	H	4.39990262	-3.03776627	-0.55181826
H	-0.18723445	-0.91750153	1.55861324	H	-0.21535860	-0.92340319	1.44470065
C	1.98386412	3.20771191	-1.79324163	C	1.99880266	3.18855071	-1.90736699
C	-0.61881908	4.31104772	-1.69121884	C	-0.60377138	4.29132732	-1.78522206
C	0.42567534	5.02563566	-2.26298155	C	0.43574269	5.00559485	-2.36525565
C	1.73608655	4.47086650	-2.31587063	C	1.74627433	4.45110020	-2.42831416
H	2.99167172	2.77784440	-1.83107232	H	3.00624721	2.75892066	-1.95371037
H	2.55095129	5.04443955	-2.77000182	H	2.55709790	5.02450293	-2.88980212
H	0.24188408	6.02246301	-2.67767355	H	0.24838243	6.00208778	-2.77915153
H	-1.62844496	4.73608625	-1.65089596	H	-1.61312731	4.71621764	-1.73692248
	**13**				**14**		
O	1.88243020	-1.95797041	0.16184673	O	1.26984788	-1.89506325	-0.05696617
C	0.51230976	-1.92285439	0.03715493	C	-0.10519508	-1.93006921	-0.12621218
C	-1.22305775	2.33835074	0.68806289	C	-2.01156249	2.24909625	0.55850125
C	-0.15594709	-0.61052513	0.13785330	C	-0.83004581	-0.65197520	-0.01038039
C	-1.61058799	-0.61196411	0.10187609	C	-2.28293041	-0.72203911	0.01628741
C	-2.42493724	0.24205458	0.80617426	C	-3.10684085	0.09970398	0.74774537
C	-1.94281656	1.39385427	1.62553193	C	-2.64543346	1.28242131	1.53457421
C	2.60765857	-0.82023011	0.43690898	C	1.95439450	-0.72205567	0.18093644
C	0.57389297	0.56681086	0.25623446	C	-0.15076223	0.55977980	0.06761283
C	2.00731216	0.45573938	0.48074472	C	1.29244292	0.52343074	0.23431633
C	3.97357453	-1.02527591	0.65662663	C	3.33908486	-0.87079300	0.35981981
C	2.86000601	1.53930517	0.81409102	C	2.09445753	1.65555236	0.52203475
C	4.81879203	0.06215385	0.97561824	C	4.11925018	0.28858807	0.61180866
C	4.22083492	1.35534847	1.04795110	C	3.47550739	1.56326756	0.69974976
H	2.43372842	2.54220443	0.90454453	H	1.61397517	2.63433322	0.62057626
N	6.16853770	-0.12645898	1.24123502	N	5.49341013	0.17153483	0.82572813
O	-0.06460583	-2.97656497	-0.18245361	C	6.29949354	1.38426575	0.75301885
C	-0.02761833	1.91017311	0.05141272	C	6.13932958	-1.04659319	0.35018778
C	-1.74893437	3.60927410	0.38703143	H	7.17156154	-1.04716486	0.73866320
C	-1.11496948	4.46031523	-0.53052033	H	6.19804070	-1.06615677	-0.76158341
C	0.60157034	2.77405171	-0.88053777	O	-0.63537800	-3.01498632	-0.31232720
C	0.06030020	4.03338286	-1.17337763	C	-0.82546953	1.87025073	-0.12511251
H	1.50144595	2.43113377	-1.40176095	C	-2.61015098	3.48999923	0.26805137
H	-2.67409285	3.92636451	0.88148688	C	-2.05804228	4.35889206	-0.68503542
H	-1.25168483	1.03664475	2.41291082	C	-0.27995323	2.75120946	-1.09312794
H	-2.80337980	1.88611149	2.10615937	C	-0.89297291	3.97988295	-1.37481385
N	-2.37307299	-1.52264046	-0.63429748	H	0.61198224	2.44418697	-1.64919347
H	-2.05812232	-2.49460855	-0.51461869	H	-0.46391245	4.63758077	-2.13807778
N	-3.69717451	-1.31730422	-0.22928900	H	-2.53867911	5.32002219	-0.89545143
C	-3.81407752	-0.16735839	0.58783489	H	-3.52724118	3.76885314	0.79927125
O	-4.86183161	0.33499411	0.99751931	H	-1.90523644	0.96688760	2.29475536
C	-4.72799616	-1.96166691	-0.95114899	H	-3.50706614	1.73916262	2.04736730
C	-6.05276893	-1.92695531	-0.46407241	C	4.28154292	2.82069668	0.96476387
C	-4.42432874	-2.66546239	-2.13633619	C	5.64286090	2.48860503	1.57477187
C	-7.06089215	-2.59430585	-1.17656166	H	4.43370392	3.36052119	0.01062029
C	-5.44592341	-3.33447515	-2.82745397	H	3.70628839	3.49559172	1.62191387
C	-6.76813116	-3.30424156	-2.35346081	H	5.52177018	2.12848938	2.61150114
H	-6.27543911	-1.37286885	0.44817155	H	6.29084914	3.38096462	1.59661933
H	-3.40218552	-2.66323664	-2.52320439	H	7.29860845	1.13983146	1.15160060
H	-8.08752803	-2.56152934	-0.79692394	H	6.43378329	1.72299475	-0.29941293
H	-5.20200390	-3.87619818	-3.74733322	C	5.36831016	-2.26119395	0.85567467
H	-7.56189853	-3.82587085	-2.89731055	C	3.95379601	-2.25192713	0.27548577
H	4.82264635	2.22945038	1.30330340	H	5.32880493	-2.21013463	1.95777634
H	4.33750939	-2.05104141	0.58189035	H	5.89441754	-3.18979437	0.57764081
C	6.76220373	-1.39893557	0.85789670	H	3.97371219	-2.57378146	-0.78287776
H	7.82405894	-1.39217274	1.14197665	H	3.30597618	-2.97417494	0.79897802
H	6.27967854	-2.22842503	1.39895437	N	-3.03112029	-1.67764992	-0.67598729
H	6.68417015	-1.60067753	-0.23018824	H	-2.66258183	-2.63133164	-0.56195309

C	7.04394074	1.03654280	1.19400219
H	6.76013848	1.77018383	1.96500378
H	8.07047823	0.71041481	1.41381800
H	7.03530699	1.54383117	0.20772144
H	-1.53979236	5.44542287	-0.74981956
H	0.55327378	4.67770572	-1.90887934

N	-4.34459206	-1.53384971	-0.21324843
C	-4.48202323	-0.37941131	0.59563860
O	-5.53482424	0.07549962	1.04575797
C	-5.37391822	-2.23033023	-0.88719823
C	-5.08962370	-2.92507181	-2.08250225
C	-6.67595900	-2.25669402	-0.34175895
C	-6.10747942	-3.64583023	-2.72541375
C	-7.68152674	-2.97501268	-1.00671743
C	-7.40738925	-3.67629646	-2.19327809
H	-4.08666053	-2.87545046	-2.51385095
H	-6.88459072	-1.70910561	0.57769234
H	-5.87883833	-4.18033423	-3.65342572
H	-8.69073216	-2.98963414	-0.58187006
H	-8.19847001	-4.23819055	-2.69966517

15

O	2.01021647	-2.56143094	0.06805250
C	0.63599510	-2.55945389	-0.01228867
C	-1.20664409	1.57437679	1.09595556
C	-0.06572662	-1.28338603	0.22414938
C	-1.52121151	-1.32724062	0.23457556
C	-2.33431982	-0.57207521	1.04439524
C	-1.86309487	0.51561830	1.95347502
C	2.71044111	-1.43019617	0.42208214
C	0.63233011	-0.09922694	0.42741522
C	2.07483092	-0.18283865	0.59997170
C	4.08848043	-1.60994672	0.57929501
C	2.90709436	0.89391131	1.00150566
C	4.91220650	-0.52875230	0.96810957
C	4.27962408	0.73300223	1.17580732
H	2.45520833	1.87069519	1.19476154
N	6.27502261	-0.69677919	1.17288750
O	0.08343646	-3.60769033	-0.30821518
C	-0.01384593	1.23912494	0.36821922
C	-1.76862438	2.83949988	0.94880995
C	-1.19883643	3.82204761	0.09233779
C	0.55539403	2.19137543	-0.48915326
C	-0.01333857	3.48306501	-0.65334735
H	1.44330734	1.92705109	-1.07583164
H	-2.68242061	3.08424427	1.50476075
H	-1.13449044	0.11100500	2.68229215
H	-2.72351207	0.92764326	2.50497018
N	-2.28111710	-2.18919281	-0.55931541
H	-1.93153522	-3.15664890	-0.54542656
N	-3.59509927	-2.06921539	-0.09267884
C	-3.71675589	-1.00681539	0.83553028
O	-4.76379643	-0.58268426	1.32743460
C	-4.63161633	-2.67284663	-0.84114421
C	-4.35028991	-3.24548468	-2.10016944
C	-5.93827760	-2.73050085	-0.30942964
C	-5.37584033	-3.87655965	-2.82045995
C	-6.95124005	-3.35735028	-1.05125540
C	-6.68030693	-3.93734823	-2.30225635
H	-3.34335672	-3.17066281	-2.51844663
H	-6.14416172	-2.27686084	0.66042283
H	-5.14944411	-4.31619861	-3.79749800
H	-7.96393180	-3.39659335	-0.63640370
H	-7.47737793	-4.42874402	-2.86895446
H	4.86414980	1.59959550	1.49012742
H	4.47964280	-2.61250130	0.40006944
C	6.89234744	-1.91078608	0.65937061
H	7.96231248	-1.89546398	0.91079709
H	6.45199480	-2.79980465	1.13825933
H	6.78449107	-2.01767781	-0.43955962
C	7.11409958	0.49328744	1.20399366
H	6.83575014	1.14510493	2.04713149
H	8.15664111	0.18176213	1.36052627
H	7.05667055	1.08663237	0.26870967
C	0.55675546	4.45357028	-1.53147655
C	-1.76639900	5.12110628	-0.06242562
C	-0.02006102	5.71052562	-1.66126561
C	-1.18883979	6.04799012	-0.92152245
H	1.45592954	4.19084683	-2.10104927
H	0.42529429	6.44802028	-2.33753289
H	-1.63502951	7.04175119	-1.03400881
H	-2.66912488	5.37610424	0.50491385

16 *ab*				16 *ac*			
O	2.10636998	-2.94899207	-0.43362732	O	2.08496202	-2.99525436	-0.29603770
C	0.75546660	-2.87859436	-0.67008676	C	0.68863541	-2.94015867	-0.30035756
C	-0.96271161	1.23931922	0.38778087	C	-0.90622035	1.25794415	0.48175362
C	0.05224781	-1.59717859	-0.43353833	C	0.07666102	-1.58808082	-0.33071026
C	-1.42512332	-1.63533082	-0.57272622	C	-1.38154488	-1.55570265	-0.56514446
C	-2.22226028	-0.80945016	0.19397842	C	-2.20424996	-0.75734267	0.20627854
C	-1.58661104	0.13114865	1.19190407	C	-1.60489022	0.15942371	1.24639384
C	2.82417343	-1.84256242	-0.05809291	C	2.84028857	-1.88498069	-0.04450167
C	0.79894268	-0.43608896	-0.24059829	C	0.83856609	-0.44231681	-0.16366139
C	2.22911099	-0.56612181	0.01977081	C	2.27254893	-0.59530033	0.06615783
C	4.17537156	-2.06118568	0.23194296	C	4.21359452	-2.10429207	0.12219251
C	3.04929181	0.50825377	0.44503621	C	3.13271823	0.47277126	0.41865324
C	4.98636980	-0.99039358	0.62679795	C	5.06514553	-1.03839082	0.43671988
C	4.39534270	0.29426300	0.73375519	C	4.49635333	0.24951693	0.59784742
H	2.62334682	1.50596519	0.57163510	H	2.72854308	1.47591573	0.57072519
O	0.22299949	-3.89312292	-1.10174771	O	0.08662613	-3.98799063	-0.29337116
O	-1.96239555	-2.48949326	-1.47085877	O	-1.80545589	-2.33971805	-1.56293066
H	-1.28063294	-3.18311129	-1.60869868	H	-2.79274897	-2.22116428	-1.57025681
C	0.21752499	0.91918890	-0.32545745	C	0.26451883	0.92153681	-0.24663976
C	-3.69519586	-0.76364038	-0.01760730	C	-3.62947649	-0.72011700	-0.13240186
O	-4.37523617	-1.52851904	-0.67304257	O	-4.16483849	-1.42633307	-0.99554232
O	-4.22757384	0.31669788	0.64301149	O	-4.32796551	0.17826792	0.59440217
C	-5.64809734	0.43399013	0.47483160	C	-5.73095684	0.22355903	0.27488238
H	-5.90807155	0.55570165	-0.58806859	H	-5.87943258	0.50200792	-0.77911930
H	-6.16446804	-0.45852772	0.86066527	H	-6.20132323	-0.75465342	0.45464863
H	-5.93978032	1.32440988	1.04695334	H	-6.15514436	0.98545921	0.94047698
C	-1.53565430	2.51723470	0.24978530	C	-1.43540336	2.55795422	0.37951992
C	-0.93763117	3.47976950	-0.57755415	C	-0.82215031	3.52270602	-0.43498646
C	0.81547899	1.89253279	-1.16324993	C	0.87055686	1.89511841	-1.07651422
C	0.23410686	3.16182984	-1.29110864	C	0.32512415	3.18351857	-1.17517760
H	1.70865182	1.63499017	-1.74272438	H	1.74979346	1.62468463	-1.67075467
H	0.69164159	3.90281533	-1.95485416	H	0.79463076	3.92224265	-1.83303728
H	-1.38715415	4.47353303	-0.67612594	H	-1.24367538	4.53110735	-0.50377559
H	-2.46010354	2.74354099	0.79171753	H	-2.34502475	2.80418788	0.93892414
H	-0.80668289	-0.39741634	1.76918091	H	-0.88282156	-0.39511987	1.87162915
H	-2.34282136	0.51417889	1.88793778	H	-2.39098234	0.56659324	1.89713538
O	6.29091482	-1.23556523	0.89651947	O	6.38760204	-1.29345532	0.59109789
H	6.69132950	-0.39009146	1.16240008	H	6.81382637	-0.45129067	0.82478371
Cl	5.40210991	1.62235187	1.26235941	Cl	5.55429356	1.57150976	1.03892921
H	4.59741314	-3.06632931	0.15180432	H	4.61931654	-3.11436320	0.02176909

5.2. Excited state (S_1) cartesian coordinates for molecular structures obtained at ADC(2)/cc-pVDZ level of theory.

3*ac*				4*ac*			
O	1.06431800	-2.55831615	1.23348920	O	0.39152804	-2.48108733	-1.13678605
C	-0.34927909	-2.53980587	1.34804922	C	-1.03329068	-2.45941825	-1.28195824
C	-1.92816001	1.52462405	-0.37814625	C	-2.58311272	1.76140057	-0.06088980
C	-1.04178933	-1.38102886	0.75891166	C	-1.71678326	-1.26487076	-0.77465731
C	-2.40099088	-1.50017134	0.53621190	C	-3.08766576	-1.35818312	-0.52990610
C	-3.20396954	-0.52035287	-0.28380930	C	-3.85728546	-0.32701404	0.19956842
C	-2.50358281	0.40829798	-1.22706400	C	-3.15482519	0.77724836	0.93555030
C	1.75844829	-1.55119431	0.61888508	C	1.07397599	-1.45611612	-0.54466352
C	-0.24910446	-0.14800721	0.44932387	C	-0.91050284	-0.01104721	-0.60916473
C	1.14730526	-0.31284785	0.26088947	C	0.47488951	-0.17904786	-0.33112511
C	3.11921847	-1.78927633	0.42065195	C	2.41574745	-1.71570728	-0.23851423
C	2.00982047	0.68794042	-0.28408301	C	1.31636697	0.85923626	0.16488499
C	3.96406183	-0.78898592	-0.12419117	C	3.22692771	-0.65933423	0.26946194
C	3.36660864	0.45851552	-0.48425823	C	2.66033504	0.64520314	0.46440078
H	3.49572674	-2.76605000	0.72991155	H	0.87787350	1.84985087	0.33465036
H	1.57262405	1.64454804	-0.58941908	N	4.55198662	-0.90311510	0.60253726
H	3.96788336	1.25412476	-0.92943108	C	5.44019392	0.20978115	0.91784648
N	5.32310617	-1.00902185	-0.28513802	C	5.18982549	-2.13031797	0.13658516
C	6.08332419	-0.09887837	-1.12837583	H	6.11717880	-2.26073391	0.71954515
H	7.13619657	-0.41522457	-1.12632308	H	5.47687473	-2.03851754	-0.93424423
H	5.71987255	-0.07969154	-2.17586850	O	-1.50204251	-3.46345254	-1.77215696
H	6.04431045	0.92710547	-0.72873546	O	-3.76218331	-2.43705492	-0.91854286
C	5.82184351	-2.37039880	-0.16425279	H	-4.71984735	-2.25154212	-0.68834607
H	6.91300008	-2.35988757	-0.29740604	C	-1.46528304	1.33561352	-0.81793798
H	5.61076435	-2.77174645	0.83988668	C	-5.29550768	-0.40375395	0.19405788
H	5.37602323	-3.05653867	-0.91268121	O	-5.96844595	-1.35021872	-0.27155115
O	-0.83665469	-3.50894507	1.88699680	O	-5.90063975	0.66653215	0.77143714
O	-3.09340758	-2.54522002	0.96216000	C	-7.33600744	0.58310487	0.78501685
H	-4.06043710	-2.30462809	0.81543695	H	-7.73275574	0.52285143	-0.23986003

C	-0.83759509	1.20671570	0.46736622	H	-7.67044471	-0.30284108	1.34619232
C	-4.62655975	-0.47365528	-0.10358596	H	-7.67425443	1.50389565	1.27707642
O	-5.29022071	-1.32171032	0.53733823	C	-3.13095772	3.03704252	-0.26261328
O	-5.22332251	0.56511151	-0.73584138	C	-2.55657668	3.91854614	-1.19966897
C	-6.65684144	0.58479900	-0.59701028	C	-0.88181074	2.22994269	-1.74516129
H	-7.10015462	-0.33078332	-1.01717884	C	-1.42937355	3.51251811	-1.93295752
H	-6.94259672	0.66474309	0.46269245	H	-0.01257351	1.90454012	-2.32821643
H	-6.98813559	1.46910140	-1.15545596	H	-0.97599736	4.19356117	-2.66183754
C	-2.49032820	2.80903128	-0.36026676	H	-2.99003536	4.91207722	-1.35681669
C	-1.95554140	3.80992949	0.47696702	H	-4.00649310	3.34389593	0.32202534
C	-0.30442084	2.21346373	1.30202090	H	-2.32803852	0.35376372	1.53612524
C	-0.86453529	3.50655503	1.30421224	H	-3.86357304	1.27506949	1.61163712
H	0.53972310	1.97228775	1.95765624	C	3.51955844	1.78377784	0.97125077
H	-0.44268434	4.27646751	1.95972984	C	4.71215765	1.24942986	1.76256458
H	-2.39720667	4.81205925	0.48631043	H	3.89162333	2.37552123	0.11225106
H	-3.34387102	3.03017749	-1.01167042	H	2.90614733	2.46521027	1.58524058
H	-3.21343796	0.80331762	-1.96745993	H	4.36773477	0.77268795	2.69742102
H	-1.68935165	-0.12654944	-1.74744528	H	5.40862848	2.06207891	2.03002734
				H	6.30631104	-0.20487539	1.46126854
				H	5.82678493	0.68137918	-0.01301011
				C	4.25047276	-3.31631808	0.32651031
				C	2.97744368	-3.09686520	-0.48883312
				H	4.00551450	-3.40234843	1.39997568
				H	4.75882720	-4.24583593	0.01940252
				H	3.19177218	-3.21669661	-1.56801323
				H	2.20962731	-3.84944661	-0.24707901
5ab + 2 dioxane				***5open ab + 2 dioxane***			
O	-0.72791321	3.16332231	0.32695566	O	-0.58255693	2.96843282	0.22466341
C	0.35813322	2.37971505	0.04920161	C	0.50279313	2.16699832	0.00682458
C	-0.96761623	-1.70759967	-1.17211418	C	-0.78203883	-2.00592761	-1.06789941
C	0.22900828	0.96253841	0.04519403	C	0.36210729	0.75212758	0.01883916
C	1.47664029	0.12516161	-0.14805624	C	1.62173819	-0.09032618	-0.05565969
C	1.45175284	-1.27796428	-0.20613037	C	1.61230313	-1.49417927	-0.06104394
C	0.34695234	-2.06586746	-0.75815706	C	0.53359645	-2.31306414	-0.62640721
C	-1.80613764	2.59004602	0.98796365	C	-1.69873498	2.41719269	0.83673036
C	-1.03243680	0.25578235	0.40516748	C	-0.91326117	0.06190570	0.37193118
C	-1.97640628	1.20167748	1.09116673	C	-1.89329793	1.03772776	0.96039832
C	-2.70255485	3.49403255	1.57029018	C	-2.62113257	3.34190767	1.34688368
C	-3.08296626	0.72690721	1.82205987	C	-3.05011789	0.59667880	1.63597305
C	-3.81192716	3.02006829	2.28589449	C	-3.77839295	2.89842338	2.00364008
C	-3.98075257	1.62082787	2.41300205	C	-3.97721106	1.50488626	2.15675773
H	-3.25151999	-0.35148579	1.91205222	H	-3.23529976	-0.47743926	1.74389846
O	1.42909748	2.98442070	-0.19499252	O	1.58310821	2.76280472	-0.22639954
O	2.58772786	0.80036015	-0.15388333	O	2.72987724	0.58800933	-0.02904390
H	2.29309922	1.79840709	-0.20094694	H	2.44261004	1.57939822	-0.14779148
C	-1.66900305	-0.53780840	-0.73014962	C	-1.50242198	-0.81332601	-0.72747827
C	2.72852585	-1.98489559	0.04076289	C	2.90104349	-2.16513650	0.24030290
O	3.85290702	-1.50189868	0.11221724	O	3.88922447	-1.65522640	0.75631399
O	2.50344692	-3.32243071	0.24679712	O	2.87366666	-3.49362941	-0.08868029
C	3.70290497	-4.06718626	0.49702133	C	4.07883008	-4.18912495	0.25478555
H	4.40209479	-3.98980648	-0.35022392	H	4.94346006	-3.76840043	-0.28133581
H	4.20925655	-3.69962766	1.40299088	H	4.27470266	-4.12643139	1.33642766
H	3.37855999	-5.10727608	0.63381107	H	3.91101141	-5.23176159	-0.04588699
C	-1.65963617	-2.58429011	-2.07107727	C	-1.44780495	-2.95711876	-1.91182430
C	-2.93002632	-2.26839487	-2.56372134	C	-2.70424489	-2.68848637	-2.46146441
C	-2.94038239	-0.23683977	-1.22791401	C	-2.75884174	-0.55952296	-1.28806785
C	-3.57121439	-1.07567121	-2.17467647	C	-3.35876348	-1.47089082	-2.18527775
H	-3.45652546	0.66196262	-0.87066149	H	-3.29134475	0.35895183	-1.01568535
H	-4.55479177	-0.81025790	-2.57694647	H	-4.33094509	-1.24145307	-2.63437102
H	-3.42212090	-2.95191914	-3.26504745	H	-3.17509852	-3.42713149	-3.12023474
H	-1.16270088	-3.51184961	-2.38233327	H	-0.93664359	-3.90163022	-2.13697901
H	0.65291859	-3.08326222	-1.03284920	H	0.86322911	-3.33433359	-0.85044083
O	-4.65863297	3.92847098	2.83369783	O	-4.71518253	3.74009858	2.51845974
H	-5.34988956	3.42759970	3.29877176	H	-4.42429939	4.64570906	2.33315350
Cl	-5.35137405	1.02749030	3.32339259	Cl	-5.38693763	0.93031702	2.99446866
H	-2.53496803	4.57044882	1.47847194	H	-2.41010969	4.41092859	1.23265983
H	-0.70904461	-0.52427592	1.13013922	H	-0.61310840	-0.65952576	1.16757681
O	0.90452379	0.98054555	2.55944790	O	0.80798932	1.20275334	2.58509236
C	0.44448043	0.03391465	3.53505937	C	0.22569854	0.40293856	3.62502660
C	1.42822063	-1.12078243	3.65416594	C	1.12786276	-0.78211161	3.93259968
O	2.70156912	-0.63541422	4.06175075	O	2.39364916	-0.32437483	4.38712341
C	3.17804415	0.29985150	3.09496106	C	2.99758205	0.45713692	3.35575480
C	2.20238839	1.45979225	2.94905679	C	2.11184964	1.64411122	3.00069006
H	-0.55259531	-0.30193859	3.20622505	H	-0.76709261	0.08880300	3.26405099
H	0.34311218	0.53895310	4.51754371	H	0.09552963	1.02124793	4.53667121

H	1.09372130	-1.83349447	4.42614404	H	0.69835834	-1.40240190	4.73654106
H	1.49828379	-1.64930559	2.68017746	H	1.23950148	-1.39794039	3.01432128
H	4.15149326	0.66528057	3.46034828	H	3.96403809	0.81078513	3.75033967
H	3.32780753	-0.19221855	2.11620248	H	3.18443466	-0.16265678	2.45895937
H	2.52428178	2.16558976	2.16716379	H	2.52732851	2.22296436	2.16068203
H	2.11175973	2.00419474	3.91090746	H	2.00192590	2.31256383	3.87876311
O	0.81693636	0.90236613	-2.44898799	O	0.88285183	0.73021011	-2.48851099
C	-0.21640148	1.51540575	-3.23144002	C	0.43991968	1.91131569	-3.17407879
C	-0.85884732	0.49531742	-4.15997118	C	-0.82589311	1.62230562	-3.96519001
O	0.11985797	-0.08311332	-5.01957494	O	-0.58691101	0.60797947	-4.93088669
C	1.10604998	-0.72890904	-4.22031506	C	-0.17945252	-0.57682356	-4.24824558
C	1.78644398	0.27974312	-3.30878789	C	1.08646540	-0.32822628	-3.44206294
H	-0.95090473	1.91844398	-2.51342390	H	0.27002955	2.68577414	-2.41031511
H	0.21735392	2.34940731	-3.82006461	H	1.24044080	2.25612942	-3.85976404
H	-1.60660692	0.98216327	-4.80755319	H	-1.15227757	2.52213259	-4.51232490
H	-1.35592500	-0.28647597	-3.55581882	H	-1.62776480	1.30901863	-3.26429146
H	1.83522049	-1.18107701	-4.91248639	H	0.00083075	-1.34489916	-5.01817008
H	0.64432162	-1.52332770	-3.59997637	H	-0.98234611	-0.92505080	-3.57254506
H	2.52988001	-0.19406033	-2.64737027	H	1.37226062	-1.21787881	-2.85912515
H	2.28304562	1.06051643	-3.92052414	H	1.91768002	-0.04423801	-4.11982167
**9*ac***				**11*ab + 2 dioxane***			
O	1.07728483	-3.33911758	-0.97324144	O	-0.31396484	3.17552476	0.58762786
C	-0.33450067	-3.28730730	-1.01814457	C	0.77894999	2.38930321	0.34948592
C	-1.78468448	0.83568454	0.70893025	C	-0.53906095	-1.58760907	-0.89717912
C	-0.97671142	-2.08261672	-0.46306804	C	0.65514070	0.96870502	0.42532239
C	-2.35114768	-2.10873928	-0.31823483	C	1.89211634	0.12424436	0.26634757
C	-3.12041913	-1.07963007	0.46972524	C	1.84508880	-1.27591669	0.19617868
C	-2.41295157	-0.27831747	1.51711548	C	0.75117495	-1.98815914	-0.48206155
C	1.82640422	-2.33521151	-0.41829736	C	-1.37480552	2.63313395	1.30467674
C	-0.14106775	-0.88107220	-0.14650997	C	-0.60972488	0.28342027	0.80879019
C	1.26360317	-1.08483549	-0.03422156	C	-1.53504367	1.25154118	1.48564992
C	3.19084265	-2.60603482	-0.29514009	C	-2.26460896	3.56190290	1.85560392
C	2.17630594	-0.11619455	0.48514815	C	-2.62957442	0.80850946	2.25198239
C	4.08786615	-1.63051438	0.20654597	C	-3.36061902	3.11952216	2.61240107
C	3.53636319	-0.37739174	0.61324623	C	-3.52242105	1.72803682	2.81161813
H	1.77959245	0.84466245	0.82901068	H	-2.79364025	-0.26487204	2.39525987
O	-0.87516805	-4.26431969	-1.48944191	O	1.84763113	2.98080405	0.08914080
O	-3.10156005	-3.09643531	-0.77994019	O	3.01289700	0.78533154	0.26144079
H	-4.04773435	-2.75843444	-0.71918124	H	2.73569284	1.77442258	0.16073678
C	-0.68029293	0.48884187	-0.14196688	C	-1.24135631	-0.42634329	-0.38558390
C	-4.50501703	-0.86445964	0.16097147	C	3.09832345	-2.00462362	0.47859072
O	-5.19024452	-1.62445431	-0.56023004	O	4.10650246	-1.54860140	1.01008517
O	-5.03979761	0.21834939	0.77144327	O	3.01309930	-3.32167695	0.11456140
C	-6.44474941	0.40091163	0.50742719	C	4.18805125	-4.07748382	0.43402112
H	-6.62132187	0.53759844	-0.57005149	H	5.07032944	-3.67661249	-0.08856060
H	-7.02125781	-0.46923874	0.85620752	H	4.38595365	-4.05789345	1.51699634
H	-6.72728921	1.30329972	1.06368893	H	3.97713209	-5.10218824	0.10059663
C	-2.29786030	2.12576971	0.71713273	C	-1.22299190	-2.38806706	-1.86037180
C	-1.71319673	3.16013973	-0.07968532	C	-2.48739774	-2.02055359	-2.39246860
C	-0.09568271	1.48704839	-0.92744439	C	-2.47264160	-0.05861448	-0.88574052
C	-0.59026352	2.82951547	-0.91873078	C	-3.11569114	-0.80424527	-1.93609572
H	0.74662154	1.23331665	-1.58304599	H	-2.99321393	0.81266261	-0.46848998
H	-3.14734198	2.36651013	1.36849582	H	-0.74166194	-3.30988077	-2.21679347
H	-1.64076093	-0.88588607	2.01777413	H	1.06585705	-2.97300960	-0.85048789
H	-3.12240915	0.11695818	2.25790462	O	-4.20046356	4.05176373	3.12933346
H	3.52378842	-3.59306179	-0.62120367	H	-4.88293215	3.57296265	3.62939442
C	-0.00432845	3.85153745	-1.71562940	Cl	-4.87780805	1.17659054	3.76902868
C	-2.21248665	4.48985558	-0.07774992	H	-2.10554056	4.63310541	1.70579444
C	-0.51565889	5.14910681	-1.69386866	H	-0.31480779	-0.52367014	1.51367732
C	-1.62346642	5.47413717	-0.87122752	O	1.27622123	1.27350270	2.90196447
H	0.85104430	3.60218979	-2.35447660	C	0.79112710	0.43195577	3.96000286
H	-0.05608836	5.92374485	-2.31739885	C	1.79684749	-0.67140139	4.25103888
H	-2.01592338	6.49642977	-0.85949300	O	3.03567712	-0.10845900	4.65964949
H	-3.06828092	4.73444408	0.56317408	C	3.53896759	0.70785141	3.60165228
N	5.45088306	-1.88011685	0.28602967	C	2.55139295	1.82182918	3.28244675
C	5.90598225	-3.25463330	0.14274839	H	-0.17903896	0.03055850	3.62536244
H	7.00339189	-3.27150587	0.20772453	H	0.63029789	1.04427293	4.87079323
H	5.49065634	-3.92729736	0.92077865	H	1.44251765	-1.31097453	5.07624709
H	5.62363494	-3.65369559	-0.84445135	H	1.93013793	-1.29023882	3.33899003
C	6.26858130	-1.00491581	1.11250619	H	4.49110240	1.13552048	3.95555888
H	5.96202067	-1.00854156	2.17850071	H	3.72829099	0.09589360	2.69980404
H	7.31490226	-1.33614836	1.04558903	H	2.89082244	2.44003173	2.43645222
H	6.22532191	0.03175889	0.74251136	H	2.41144548	2.47255318	4.16930586
H	4.17457250	0.39831713	1.04174476	O	1.21084152	0.90781514	-2.09665263
				C	0.24699048	1.64688720	-2.85847839

				C	-0.43436308	0.73989079	-3.87259514
				O	0.52834130	0.16152424	-4.75010429
				C	1.44552824	-0.60610043	-3.97737765
				C	2.16090498	0.28553514	-2.97458611
				H	-0.48116915	2.04905354	-2.13364252
				H	0.75308466	2.48822324	-3.37500292
				H	-1.13590846	1.31639313	-4.49776044
				H	-0.99281511	-0.05045343	-3.33648479
				H	2.16626148	-1.05298855	-4.68181232
				H	0.91321245	-1.41126042	-3.43235631
				H	2.84592473	-0.28953797	-2.33019663
				H	2.73294997	1.07029941	-3.51150849
				C	-3.14286973	-2.78725019	-3.40089694
				C	-4.35528311	-0.40605697	-2.49023235
				C	-4.97609050	-1.17172370	-3.48230685
				C	-4.36298612	-2.36808551	-3.93097947
				H	-2.67052185	-3.71031633	-3.75782518
				H	-4.85064747	-2.97045092	-4.70565420
				H	-5.93489095	-0.85262144	-3.90460844
				H	-4.82627378	0.51686217	-2.12871607
**11*open ab + 2 dioxane***				**14 (S_2 state)**			
O	-0.30323983	3.14597752	0.72970331	O	1.25038020	-1.59893963	-0.87079434
C	0.78111898	2.37343095	0.43154849	C	-0.16450848	-1.65278398	-0.85699810
C	-0.53486384	-1.62175442	-0.86734354	C	-2.01407673	2.51982108	0.14165476
C	0.67372995	0.94661421	0.46561171	C	-0.87325167	-0.47538843	-0.44041892
C	1.89163925	0.13861841	0.13083823	C	-2.26749657	-0.58239388	-0.26370633
C	1.88231153	-1.26605324	0.06371305	C	-3.12932460	0.29210957	0.44136985
C	0.74533328	-2.04518986	-0.42917778	C	-2.75925816	1.59214919	1.07519970
C	-1.38283098	2.56543479	1.37915231	C	1.89507677	-0.56331305	-0.24578486
C	-0.57436578	0.23931520	0.86275297	C	-0.16340180	0.80618236	-0.17972191
C	-1.51625346	1.18257767	1.54927821	C	1.24152064	0.66208269	0.07966365
C	-2.31516997	3.46636944	1.91215793	C	3.25582963	-0.78055995	0.01340669
C	-2.63603727	0.71196264	2.26407577	C	2.03728079	1.68262620	0.67398430
C	-3.43191215	2.99168837	2.61565381	C	4.02635523	0.27288565	0.58686598
C	-3.57308301	1.59664739	2.80796863	C	3.39882672	1.51872411	0.92438359
H	-2.78507898	-0.36629321	2.38760493	H	1.54857609	2.62331689	0.95287690
O	1.83754875	2.98402546	0.16037360	N	5.37312264	0.08028249	0.84430176
O	2.99252370	0.81881201	0.02539916	C	6.21016650	1.19273704	1.28577700
H	2.69433977	1.81631813	0.03631308	C	6.05710207	-1.09091199	0.30120168
C	-1.20985322	-0.44712397	-0.35347555	H	6.99900767	-1.20990665	0.86189516
C	3.15660552	-1.96386456	0.30814773	H	6.31873701	-0.91871407	-0.76521637
O	4.28775116	-1.49015061	0.30104860	O	-0.64426614	-2.74241753	-1.18564881
O	2.91454250	-3.26837214	0.65867197	C	-0.75922801	2.12825385	-0.40873536
C	4.10554763	-3.99597611	0.98878765	C	-2.52862947	3.79625569	-0.14896550
H	4.78868096	-4.05405079	0.12698500	C	-1.82624170	4.70646624	-0.95778749
H	4.63314716	-3.51327296	1.82585417	C	-0.05438880	3.06058130	-1.21808825
H	3.76493698	-4.99953268	1.27633441	C	-0.58029817	4.33199547	-1.48975582
C	-1.24190089	-2.42024719	-1.81209132	H	0.89858232	2.75447052	-1.66548591
C	-2.50417736	-2.03468106	-2.33546090	H	-0.02290491	5.02459407	-2.13050948
C	-2.43524619	-0.05281199	-0.85686887	H	-2.25015367	5.69313035	-1.17309204
C	-3.10812895	-0.80466544	-1.88128528	H	-3.49758966	4.08223870	0.27764258
H	-2.94259675	0.82161953	-0.43066611	H	-2.12213323	1.39781251	1.96369904
H	-0.78189800	-3.35468032	-2.16292318	H	-3.68826863	2.06442505	1.43757443
H	1.01514692	-3.07278066	-0.70833938	C	4.20924398	2.64487025	1.52716670
O	-4.37932940	3.80881607	3.15343140	C	5.44105436	2.09523716	2.24411703
Cl	-4.93607613	0.98935002	3.69730182	H	4.53391025	3.33524764	0.72450514
H	-2.15678415	4.54064331	1.76697233	H	3.57436863	3.22995575	2.21372385
H	-0.30402931	-0.59759409	1.55036808	H	5.13815589	1.50543080	3.12738209
O	1.29179925	1.50746059	2.94964881	H	6.09951453	2.91047883	2.58792611
C	0.73125079	0.61436292	3.92588631	H	7.09951979	0.75936591	1.77330004
C	1.45573719	-0.71863211	3.85080888	H	6.56212925	1.77930662	0.40940345
O	2.82364704	-0.54246832	4.19881511	C	5.17282318	-2.32609690	0.43258635
C	3.43295823	0.36934750	3.28830593	C	3.87660581	-2.11090618	-0.34574703
C	2.68603030	1.69858111	3.24599111	H	4.95326068	-2.49153833	1.50221725
H	-0.34112619	0.52385393	3.69587436	H	5.71543746	-3.20991550	0.05762686
H	0.85256287	1.05179205	4.93755854	H	4.07712026	-2.14086910	-1.43383327
H	1.02990766	-1.44444833	4.56356100	H	3.14846256	-2.91373058	-0.14821333
H	1.35003730	-1.11598219	2.81580360	N	-3.01777514	-1.65045203	-0.73936095
H	4.46332872	0.53086690	3.64513576	H	-2.54717966	-2.56425472	-0.69470395
H	3.48797906	-0.07483033	2.28001620	N	-4.27753656	-1.56855779	-0.14261828
H	3.08402254	2.35262479	2.45294967	C	-4.42812462	-0.32030648	0.55192276
H	2.77411063	2.21744351	4.22162247	O	-5.47196397	0.07512498	1.08365490
O	1.30937479	1.16001449	-2.14456285	O	-5.29288168	-2.41179146	-0.61778021
C	0.21439758	1.67397547	-2.91238612	C	-5.03600113	-3.28187858	-1.70438546
C	-0.38246380	0.58195065	-3.78895110	C	-6.55781370	-2.42144049	0.01804828

O	0.60681318	0.04212534	-4.66155965	C	-6.04024600	-4.15456080	-2.14580126
C	1.66464888	-0.49717950	-3.87492398	C	-7.54731519	-3.30024651	-0.44423461
C	2.29578981	0.59276736	-3.02291637	C	-7.29863086	-4.17228627	-1.51915399
H	-0.52802064	2.04745222	-2.18631483	H	-4.07052814	-3.24628835	-2.21599941
H	0.56815405	2.51733298	-3.54050843	H	-6.74164744	-1.73718576	0.84697473
H	-1.18804261	0.98607072	-4.42370463	H	-5.83528853	-4.82086875	-2.99027794
H	-0.79695551	-0.21263165	-3.14184993	H	-8.52516460	-3.30318821	0.04874405
H	2.40248330	-0.92353428	-4.57418515	H	-8.07865325	-4.85646698	-1.86797750
H	1.28293437	-1.29770291	-3.20821229				
H	3.10380160	0.20205115	-2.38171211				
H	2.69934079	1.39114175	-3.67817662				
C	-3.18390991	-2.79828031	-3.32979525				
C	-4.35191678	-0.39484808	-2.41847170				
C	-4.99965228	-1.16231510	-3.39125426				
C	-4.40784892	-2.36898625	-3.84200021				
H	-2.73139519	-3.73278449	-3.68246749				
H	-4.91618373	-2.96942502	-4.60472951				
H	-5.96410387	-0.83845283	-3.79665134				
H	-4.80283769	0.53776613	-2.05631699				
H	-4.12870131	4.72439429	2.95125656				
**16 *ac***							
O	2.193661866	-2.917357136	-0.737867184				
C	0.788539145	-2.920034526	-0.922990193				
C	-0.888452177	1.245905513	0.458113785				
C	0.071373908	-1.704484980	-0.508474819				
C	-1.307416554	-1.786858294	-0.393263014				
C	-2.165648741	-0.754994536	0.319082843				
C	-1.527087234	0.165652538	1.311026688				
C	2.856683818	-1.853529267	-0.193606302				
C	0.835642814	-0.460049707	-0.219905829				
C	2.225463340	-0.593717343	0.041375940				
C	4.214875996	-2.048219846	0.070095804				
C	3.039697719	0.467315497	0.539360328				
C	5.005430746	-1.000516520	0.569745251				
C	4.390485472	0.256263830	0.799862585				
H	2.590759299	1.442255253	0.749152705				
O	0.322852243	-3.942470400	-1.374974910				
O	-1.991143644	-2.835861995	-0.791273218				
H	-2.967375662	-2.542802102	-0.771054161				
C	0.233054631	0.888497691	-0.330124884				
C	-3.549709854	-0.664163969	-0.032022513				
O	-4.153309437	-1.527162020	-0.716993045				
O	-4.184323643	0.405935376	0.490829722				
C	-5.595926241	0.452199094	0.196140133				
H	-5.761462863	0.498609063	-0.890559692				
H	-6.103504000	-0.437216633	0.598777269				
H	-5.960941523	1.364134145	0.684015972				
C	-1.450682262	2.526392837	0.350901807				
C	-0.885383208	3.484314118	-0.516335830				
C	0.789027657	1.847372765	-1.202556152				
C	0.228614920	3.138469714	-1.292776571				
H	1.654605341	1.574579366	-1.816298055				
H	0.672695550	3.872366618	-1.974067395				
H	-1.324630411	4.484665177	-0.590095077				
H	-2.327343362	2.779529021	0.958013335				
H	-0.760630921	-0.372411783	1.895437229				
H	-2.281916028	0.589523989	1.988491339				
O	6.319989255	-1.242415378	0.806895623				
H	6.701080770	-0.414603531	1.146565773				
Cl	5.380905275	1.556421653	1.431733829				
H	4.671448000	-3.021533293	-0.130379114				
